# Supplementary material for: Stabilized epithelial phenotype of cancer cells in primary tumors leads to increased colonization of liver metastasis in pancreatic cancer
Source: Cell Rep. Author manuscript; Available in PMC 2021 Apr 27. (PMC8078733; doi:10.1016/j.celrep.2021.108990)
Supplement: 1 [file NIHMS1693705-supplement-1.pdf]

Cell Reports, Volume *35*

## Supplemental information

# Stabilized epithelial phenotype of cancer cells in primary tumors leads to increased colonization of liver metastasis in pancreatic cancer

**Julienne L. Carstens, Sujuan Yang, Pedro Correa de Sampaio, Xiaofeng Zheng, Souptik Barua, Kathleen M. McAndrews, Arvind Rao, Jared K. Burks, Andrew D. Rhim, and Raghu Kalluri**

Figure S1

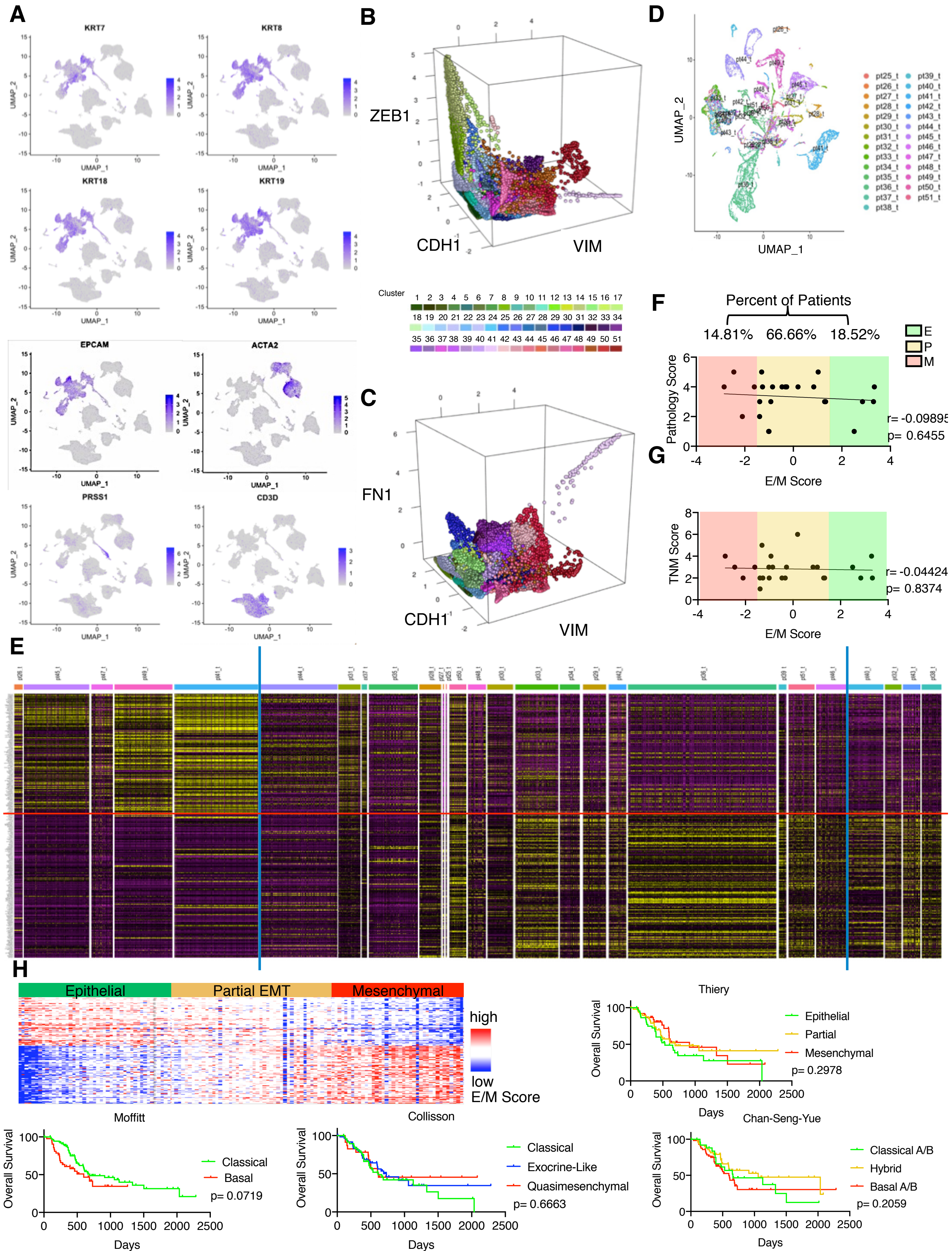
A
B
C
D
E
F
G
H
KRT7
KRT8
KRT18
KRT19
EPCAM
ACTA2
PRSS1
CD3D
UMAP_1
UMAP_2
ZEB1
CDH1
VIM
FN1
Cluster
Percent of Patients
14.81%
66.66%
18.52%
E
P
M
Pathology Score
E/M Score
r= -0.09895
p= 0.6455
TNM Score
r= -0.04424
p= 0.8374
Epithelial
Partial EMT
Mesenchymal
high
low
E/M Score
Thiery
Overall Survival
Days
Epithelial
Partial
Mesenchymal
p= 0.2978
Moffitt
Classical
Basal
p= 0.0719
Collisson
Classical
Exocrine-Like
Quasimesenchymal
p= 0.6663
Chan-Seng-Yue
Classical A/B
Hybrid
Basal A/B
p= 0.2059

**Figure S1. Single-cell RNA-sequencing of human PDAC reveals 51 different cancer cell phenotypes across the EMT continuum, Related to Figure 1.**

**A** Seurat UMAP clustering of whole tissue single-cell populations with indicated gene expression heatmap overlays to demonstrate the selection of the cancer cell population as being high in *KRT7, KRT8, KRT18, KRT19*, and *EPCAM* but low in *ACTA2, PRSS1* and *CD3D*.

**B-C** Same clusters as **Figure 1B** plotted for the expression of (**b**) *ZEB1*, *CDH1* and *VIM* or (**c**) *FN1*, *CDH1* and *VIM*.

**D** UMAP clustering of patient cancer cells post MAGIC using the Thiery EMT signature color coded for each patient.

**E** Expression heatmap of the Thiery EMT signature for each patient. Epithelial genes are above the red line. Blue line indicates the cutoff to separate epithelial, partial, and mesenchymal phenotypes.

**F-G** Correlation plots comparing the tumor pathology score (**F**) and the TNM score (**G**) against the E/M score of each patient. The percentage of patients for each E/M grouped phenotype is listed above.

**H** Gene expression heatmap of the Thiery EMT signature in the TCGA pancreatic cancer cohort and survival plots for each indicated expression classifier.

Figure S2

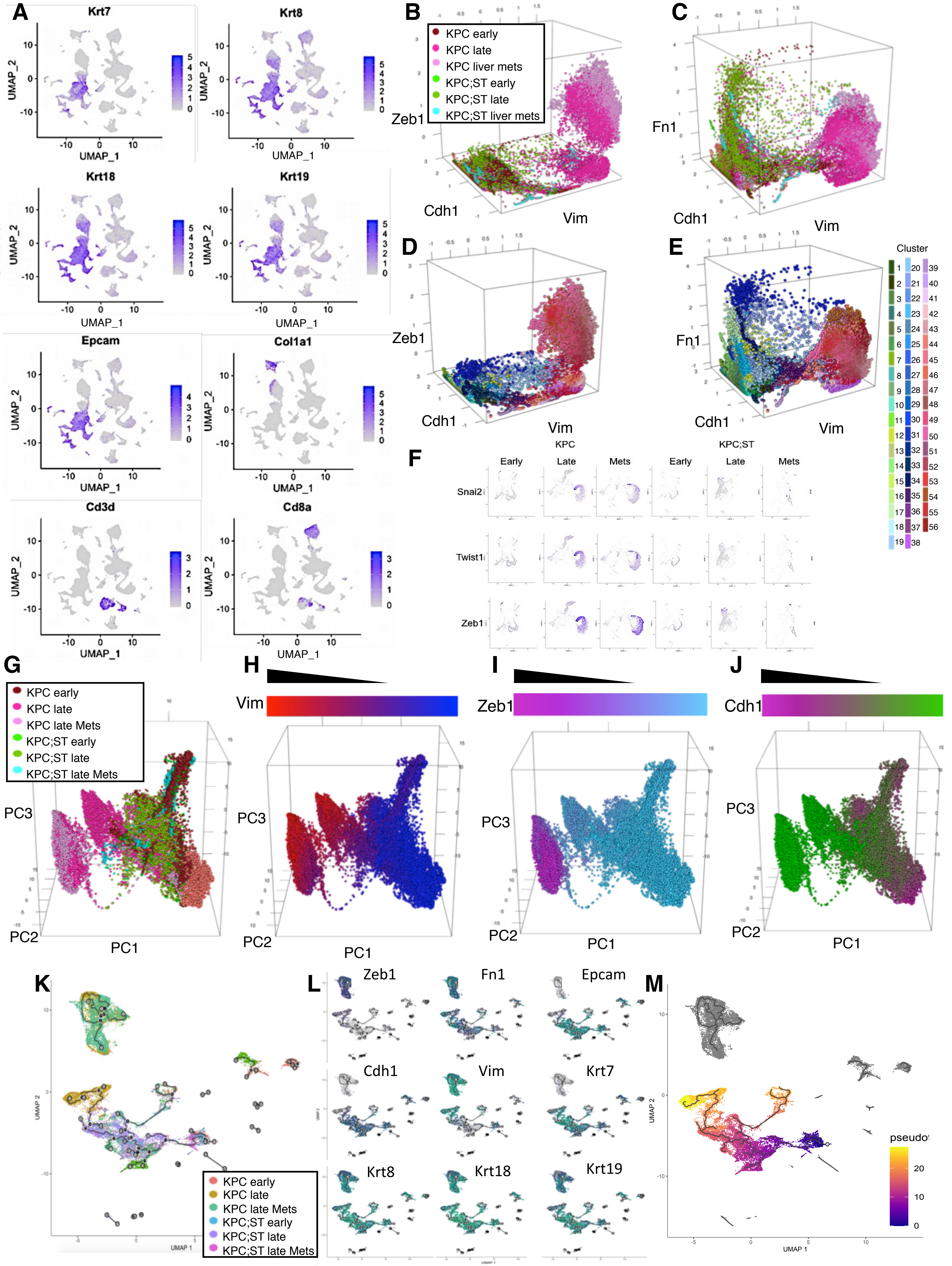

A
Krt7
Krt8
Krt18
Krt19
Epcam
Col1a1
Cd3d
Cd8a
UMAP_1
UMAP_2
B
KPC early
KPC late
KPC liver mets
KPC;ST early
KPC;ST late
KPC;ST liver mets
Zeb1
Cdh1
Vim
C
Fn1
Cdh1
Vim
D
Zeb1
Cdh1
Vim
E
Fn1
Cdh1
Vim
Cluster
F
KPC
KPC;ST
Early
Late
Mets
Snai2
Twist1
Zeb1
G
KPC early
KPC late
KPC late Mets
KPC;ST early
KPC;ST late
KPC;ST late Mets
PC3
PC2
PC1
H
Vim
I
Zeb1
J
Cdh1
K
KPC early
KPC late
KPC late Mets
KPC;ST early
KPC;ST late
KPC;ST late Mets
UMAP 1
UMAP 2
L
Zeb1
Fn1
Epcam
Cdh1
Vim
Krt7
Krt8
Krt18
Krt19
M
pseudot
UMAP 1
UMAP 2

**Figure S2. Single-cell RNA-sequencing of murine PDAC identifies 56 cancer cell phenotypes across the EMT spectrum, Related to Figure 2.**

**A** Seurat UMAP clustering of whole tissue single-cell populations with indicated gene expression heatmap overlays to demonstrate the selection of the cancer cell population as being high in *Krt7, Krt8, Krt18, Krt19*, and *Epcam*, but low in *Col1A1*, *Cd3d* and *Cd8a*.

**B-E** post-MAGIC clusters plotted for the expression of *Zeb1*, *Cdh1* and *Vim* (**B** and **D**) and *Fn1*, *Cdh1* and *Vim* (**C** and **E**) overlaid with (**B-C**) GEMM cohort or (**D-E**) E/M cluster identification.

**F** Magic cancer cell clusters separated by group and overlaid with expression heatmaps of EMT transcription factor genes.

**G-J** Cancer cell PCA analysis overlaid with cohort identifications or heatmaps for *Vim*, *Zeb1* or *Cdh1*, respectively.

**K** Trajectory analysis on Seurat cancer cell clusters color-coded by group.

**L** Expression overlays for indicated genes.

**M** pseudotime analysis.

Figure S3

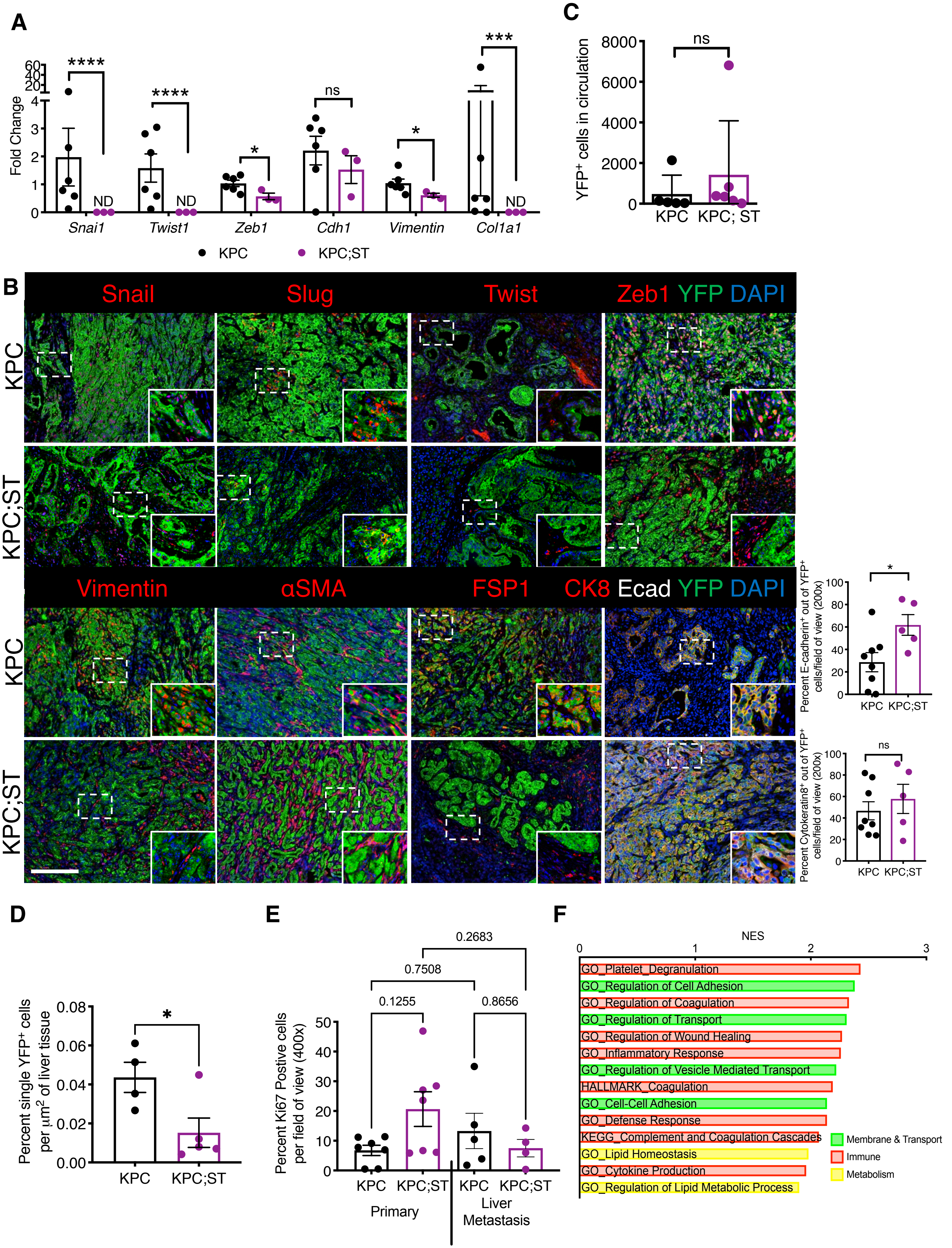
A
Fold Change
Snai1
Twist1
Zeb1
Cdh1
Vimentin
Col1a1
ND
ns
KPC
KPC;ST
B
Snail
Slug
Twist
Zeb1 YFP DAPI
Vimentin
αSMA
FSP1
CK8 Ecad YFP DAPI
Percent E-cadherin+ out of YFP+ cells/field of view (200x)
Percent Cytokeratin8+ out of YFP+ cells/field of view (200x)
C
YFP+ cells in circulation
KPC; ST
D
Percent single YFP+ cells per µm2 of liver tissue
E
Percent Ki67 Postive cells per field of view (400x)
0.2683
0.7508
0.1255
0.8656
Primary
Liver Metastasis
F
NES
GO_Platelet_Degranulation
GO_Regulation of Cell Adhesion
GO_Regulation of Coagulation
GO_Regulation of Transport
GO_Regulation of Wound Healing
GO_Inflammatory Response
GO_Regulation of Vesicle Mediated Transport
HALLMARK_Coagulation
GO_Cell-Cell Adhesion
GO_Defense Response
KEGG_Complement and Coagulation Cascades
GO_Lipid Homeostasis
GO_Cytokine Production
GO_Regulation of Lipid Metabolic Process
Membrane & Transport
Immune
Metabolism

**Figure S3. Stabilized epithelial phenotype of PDAC cells enhances liver metastasis, Related to Figure 3.**

**A** Fold change of qPCR gene expression analysis of *Snai1, Twist1, Zeb1, Cdh1, Vimentin* and *Col1a1*. (n= 6 or 5(*Col1a1*) and 3 YFP sorted individual primary cell lines, respectively).

**B** Representative immunofluorescence histological micrographs of primary pancreatic tumors for YFP (green) and indicated mesenchymal markers (red): Snail/Slug, Slug, Twist, Zeb1, Vimentin, αSMA, or FSP1 and DAPI (blue) or E-cadherin (white) and Cytokeratin 8 (red) (n=8 and 5 mice, respectively). Data presented as the mean ± s.e.m.

**C** Number of $YFP^+$ cells in 1 ml of blood from KPC control (n=5) and KPC; ST (n = 6) mice.

**D** Percent $YFP^+$ single cells per $um^2$ of liver tissue in KPC control (n=4) and KPC; ST (n = 5) mice.

**E** Quantification of percent Ki-$67^+$ cells in immunostained histological micrographs. ANOVA.

**F** Select enriched gene sets comparing Clusters 30, 32 and 34 vs. all other cancer cell clusters, all pathways displayed are significant with a nominal p-value and FDRqvalue <0.05.

Unless otherwise specified, data presented as the means ± SD. and significance was determined by an unpaired two-tailed t-test. Scale bar equals 100 μm. ns = not significant, * p<0.05, *** p<0.001, **** p<0.0001.

Figure S4

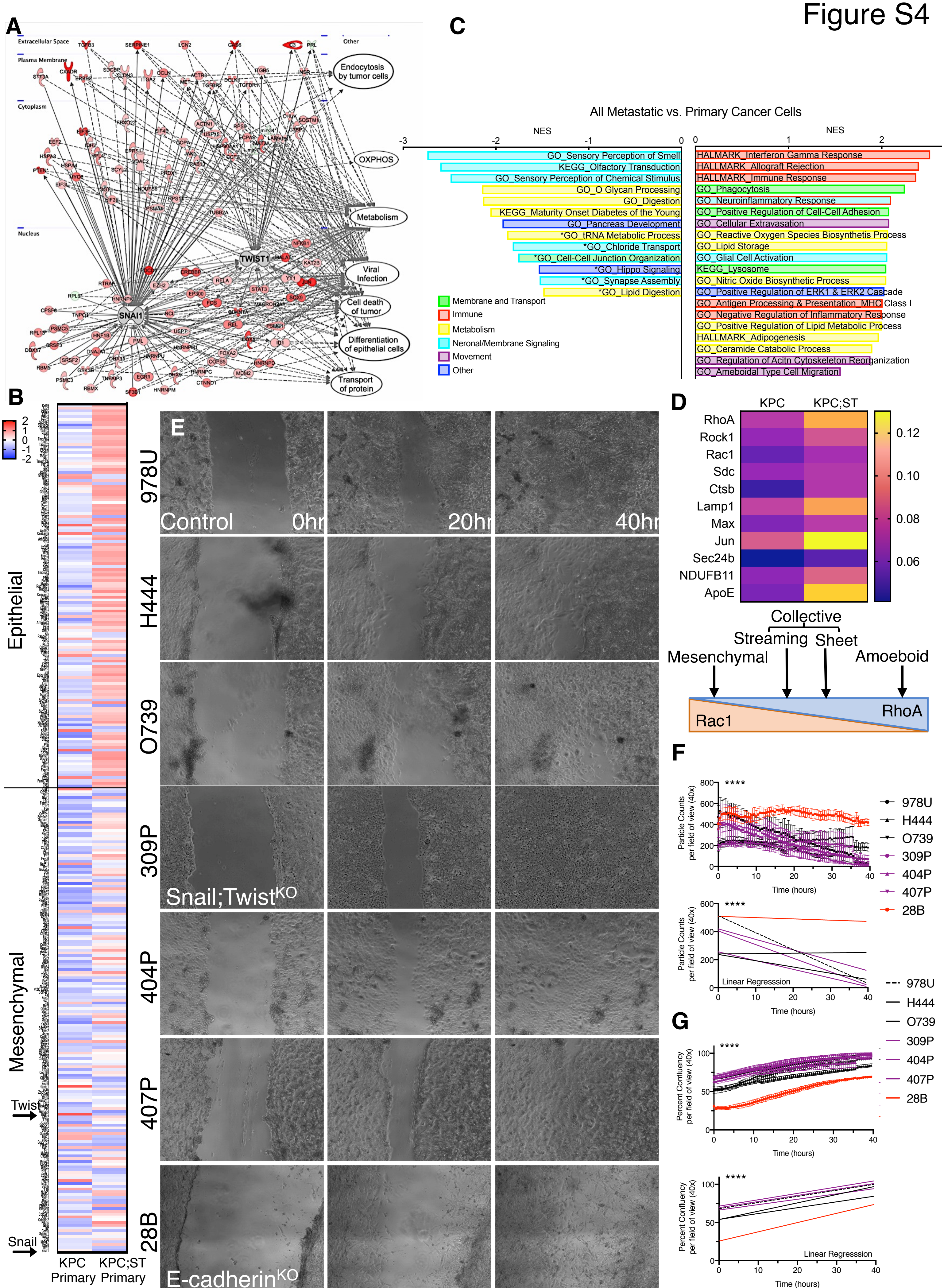

A
Extracellular Space
Plasma Membrane
Cytoplasm
Nucleus
Other
Endocytosis by tumor cells
OXPHOS
Metabolism
Viral Infection
Cell death of tumor
Differentiation of epithelial cells
Transport of protein
SNAI1
TWIST1
C
All Metastatic vs. Primary Cancer Cells
NES
GO_Sensory Perception of Smell
KEGG_Olfactory Transduction
GO_Sensory Perception of Chemical Stimulus
GO_O Glycan Processing
GO_Digestion
KEGG_Maturity Onset Diabetes of the Young
GO_Pancreas Development
*GO_tRNA Metabolic Process
*GO_Chloride Transport
*GO_Cell-Cell Junction Organization
*GO_Hippo Signaling
*GO_Synapse Assembly
*GO_Lipid Digestion
HALLMARK_Interferon Gamma Response
HALLMARK_Allograft Rejection
HALLMARK_Immune Response
GO_Phagocytosis
GO_Neuroinflammatory Response
GO_Positive Regulation of Cell-Cell Adhesion
GO_Cellular Extravasation
GO_Reactive Oxygen Species Biosynthetis Process
GO_Lipid Storage
GO_Glial Cell Activation
KEGG_Lysosome
GO_Nitric Oxide Biosynthetic Process
GO_Positive Regulation of ERK1 & ERK2 Cascade
GO_Antigen Processing & Presentation_MHC Class I
GO_Negative Regulation of Inflammatory Response
GO_Positive Regulation of Lipid Metabolic Process
HALLMARK_Adipogenesis
GO_Ceramide Catabolic Process
GO_Regulation of Acitn Cytoskeleton Reorganization
GO_Ameboidal Type Cell Migration
Membrane and Transport
Immune
Metabolism
Neronal/Membrane Signaling
Movement
Other
B
Epithelial
Mesenchymal
Twist
Snail
KPC Primary
KPC;ST Primary
E
978U
H444
O739
309P
404P
407P
28B
Control
0hr
20hr
40hr
Snail;Twist^KO
E-cadherin^KO
D
KPC
KPC;ST
RhoA
Rock1
Rac1
Sdc
Ctsb
Lamp1
Max
Jun
Sec24b
NDUFB11
ApoE
0.12
0.10
0.08
0.06
Collective
Streaming
Sheet
Mesenchymal
Amoeboid
RhoA
Rac1
F
Particle Counts per field of view (40x)
Time (hours)
****
978U
H444
O739
309P
404P
407P
28B
Linear Regresssion
G
Percent Confluency per field of view (40x)
Time (hours)
Linear Regresssion

**Figure S4. Stabilized epithelial PDAC cells migrate by collective cell migration, Related to Figure 4.**

**A** IPA pathway analysis of primary KPC control versus KPC;ST cancer cells.

**B** Z-score of the Thiery EMT signature demonstrating an overall stabilization of epithelial genes in the KPC;ST primary tumors.

**C** Select enriched gene sets comparing metastatic cancer cells vs. primary cancer cells, all pathways displayed are significant with a nominal p-value, FDRqvalue and FWER p-value <0.05. *only the nominal p-value <0.05.

**D** Mean qPCR 1/deltaCT values of YFP+ lineage traced primary cancer cells; schematic of RhoA and Rac1 expression in migration types.

**E** Representative micrographs (40x) of each primary cell line.

**F** and **G** Quantifications of the particle counts (**F**) and percent cellular confluency (**G**) with linear regressions (**** p< 0.001) of the grouped values for each cell line represented.

# Figure S5

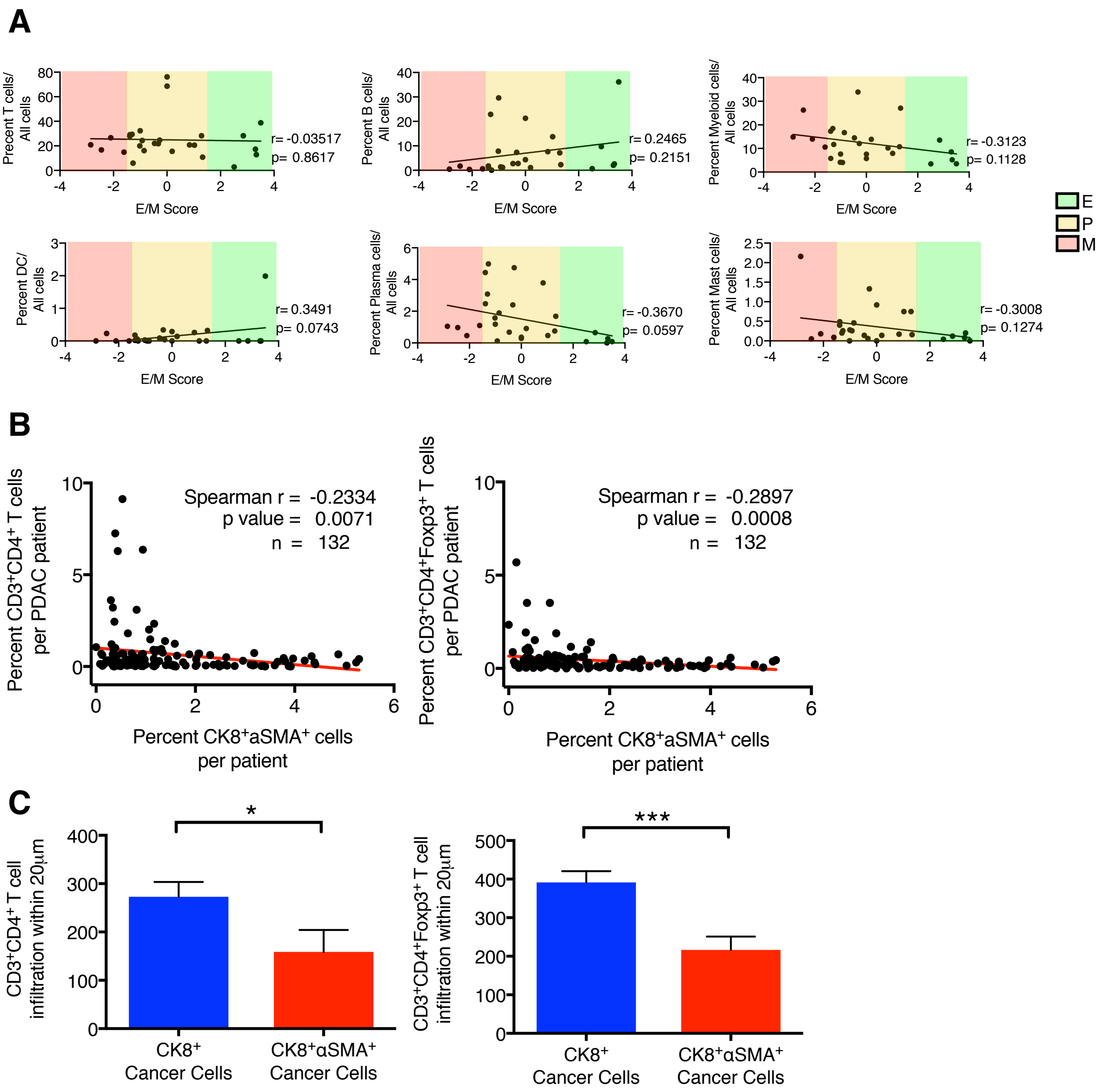

A
Percent T cells/ All cells
r= -0.03517
p= 0.8617
Percent B cells/ All cells
r= 0.2465
p= 0.2151
Percent Myeloid cells/ All cells
r= -0.3123
p= 0.1128
E
P
M
Percent DC/ All cells
r= 0.3491
p= 0.0743
Percent Plasma cells/ All cells
r= -0.3670
p= 0.0597
Percent Mast cells/ All cells
r= -0.3008
p= 0.1274
E/M Score
B
Percent CD3+CD4+ T cells per PDAC patient
Spearman r = -0.2334
p value = 0.0071
n = 132
Percent CK8+aSMA+ cells per patient
Percent CD3+CD4+Foxp3+ T cells per PDAC patient
Spearman r = -0.2897
p value = 0.0008
n = 132
Percent CK8+aSMA+ cells per patient
C
CD3+CD4+ T cell infiltration within 20µm
*
CK8+ Cancer Cells
CK8+αSMA+ Cancer Cells
CD3+CD4+Foxp3+ T cell infiltration within 20µm
***
CK8+ Cancer Cells
CK8+αSMA+ Cancer Cells

**Figure S5. Epithelial PDAC cells associate with more T cells, Related to Figure 5.**

**A** Correlation plots comparing the percent of indicated immune cells to the E/M score of each scRNA-seq patient tumor.

**B** Inverse correlation of between $CD4^{+}$ or $CD4^{+}FOXP3^{+}$ T cells and $CK8^{+}\alpha SMA^{+}$ cancer cells from multiplexed immunohistochemistry stained tissues.

**C** L-function area under the curve values (reflecting the number of cells) for $CD4^{+}$ or $CD4^{+}FOXP3^{+}$ T cells within 20 um of $CK8^{+}$ or $CK8^{+}\alpha SMA^{+}$ cancer cells.

**D** Quantification of percent Ki-$67^{+}$ cells in immunostained histological micrographs. ANOVA. * $p< 0.05$, *** $p<0.001$.

# Figure S6

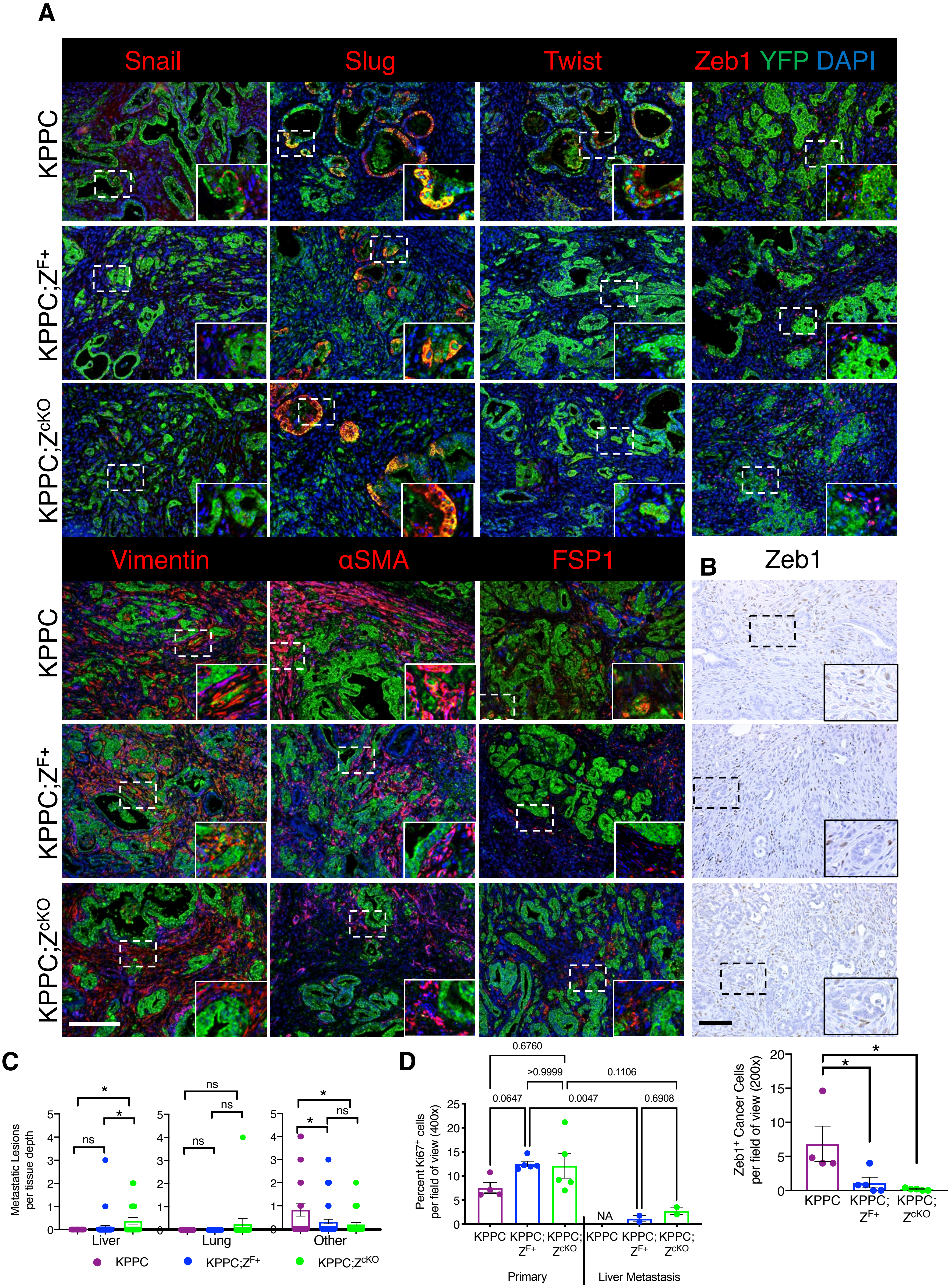
A
Snail
Slug
Twist
Zeb1 YFP DAPI
KPPC
KPPC;ZF+
KPPC;ZcKO
Vimentin
αSMA
FSP1
B
Zeb1
C
Metastatic Lesions per tissue depth
Liver
Lung
Other
ns
*
KPPC
KPPC;ZF+
KPPC;ZcKO
D
Percent Ki67+ cells per field of view (400x)
0.6760
>0.9999
0.1106
0.0647
0.0047
0.6908
NA
KPPC
KPPC; ZF+
KPPC; ZcKO
Primary
Liver Metastasis
Zeb1+ Cancer Cells per field of view (200x)

**Figure S6. Stabilization of epithelial PDAC cells via Zeb1 ablation also enhances liver metastasis, Related to Figure 6.**

**A** Representative histological micrographs of primary pancreatic tumors for YFP (green) and indicated mesenchymal markers (red): Snail/Slug, Slug, Twist, Zeb1, Vimentin, αSMA, or FSP1 and DAPI (blue).

**B** Immunohistochemical labeling for Zeb1, counterstained with hematoxylin and quantifications of Zeb1 positive cancer cells per image per mouse (n=4, 5, and 5 mice, respectively).

**C** Quantification of the metastatic leisions per tissue depth for liver (KPPC (n = 16 depths, 4 mice), KPPC;$Z^{F/+}$ (n = 48 depths, 12 mice) and KPPC; $Z^{cKO}$ (n = 20 depths, 5 mice)), lung (KPPC (n = 12 depths, 4 mice), KPPC;$Z^{F/+}$ (n = 36 depths, 12 mice) and KPPC; $Z^{cKO}$ (n = 15 depths, 5 mice)), and other tissues (KPPC (n = 50 depths, 4 mice), KPPC;$Z^{F/+}$ (n = 72 depths, 12 mice) and KPPC; $Z^{cKO}$ (n = 30 depths, 5 mice)).

Unless otherwise specified, data presented as the mean ± s.e.m., scale bar equals 100 μm, and significance determined by a one-way ANOVA or the Kruskal-Wallis test when the data were not normally distributed. ns = not significant, * $p<0.05$.

**Table S1. Clinical correlations between PDAC classification subtypes, Related to Figure 1.**

| Group | Parameter | Pearson Chi-Square |
|---|---|---|
| **Thiery EMT – sc-seq** | | |
| EvPvM | Sex | 0.350 |
| EvPvM | Diabetes | 0.842 |
| EvPvM | Procedure | 0.599 |
| EvPvM | Location | 0.773 |
| EvPvM | T (TNM Classification) | 0.787 |
| EvPvM | N (TNM Classification) | 0.583 |
| EvPvM | M (TNM Classification) | NA - all M0 |
| EvPvM | Staging | 0.625 |
| EvPvM | Perineural Invasion | 0.435 |
| EvPvM | Vascular Invasion | 0.535 |
| EvPvM | Peripancreatic Invasion | 0.580 |
| **Thiery EMT -TCGA** | | |
| EvPvM | Sex | 0.933 |
| EvPvM | Histologic Grade | 0.216 |
| EvPvM | Tumor Stage | 0.078 |
| EvPvM | T (TNM Classification) | 0.461 |
| EvPvM | N (TNM Classification) | 0.186 |
| EvPvM | M (TNM Classification) | 0.096 |
| EvPvM | Radiation | 0.646 |
| **Moffitt -TCGA** | | |
| Classical v Basal | Sex | 0.706 |
| Classical v Basal | Histologic Grade | 0.096 |
| Classical v Basal | Tumor Stage | 0.772 |
| Classical v Basal | T (TNM Classification) | 0.810 |
| Classical v Basal | N (TNM Classification) | 0.588 |
| Classical v Basal | M (TNM Classification) | 0.095 |
| Classical v Basal | Radiation | 0.791 |
| **Collisson -TCGA** | | |
| Classical v Exocrine v QM | Sex | 0.345 |
| Classical v Exocrine v QM | Histologic Grade | 0.225 |
| Classical v Exocrine v QM | Tumor Stage | **0.013** *StageIIb with QM |
| Classical v Exocrine v QM | T (TNM Classification) | **0.022** *T2 with QM |
| Classical v Exocrine v QM | N (TNM Classification) | 0.406 |
| Classical v Exocrine v QM | M (TNM Classification) | 0.153 |
| Classical v Exocrine v QM | Radiation | 0.247 |
| **Chang-Seng-Yue-TCGA** | | |
| Classical v Hybrid v Basal | Sex | 0.930 |
| Classical v Hybrid v Basal | Histologic Grade | 0.061 |
| Classical v Hybrid v Basal | Tumor Stage | 0.078 |
| Classical v Hybrid v Basal | T (TNM Classification) | 0.783 |
| Classical v Hybrid v Basal | N (TNM Classification) | 0.121 |

| | | |
|---|---|---|
| Classical v Hybrid v Basal | M (TNM Classification) | 0.059 |
| Classical v Hybrid v Basal | Radiation | **0.0022** *less in Classical |
| **Cross Groups** | **Significant Groups** | |
| Thiery v Moffitt | | 0.572 |
| Thiery v Collisson | E with Classical, M with QM | **0.000000007** |
| Thiery v Chang-Seng-Yue | E with Classical, P&M with Hybrid and Basal A/B | **0.00000008** |

**P< 0.05**

**Table S2. Survival and Pathology of murine cohorts, Related to Figure 3 and 6.**

**Survival and pathology of murine cohorts**

| | | | | | | **Presence of Metastasis** | | | | |
|---|---|---|---|---|---|---|---|---|---|---|
| **ID** | **Sex** | **Age at Palpable Tumor** | **Age at Death** | **PDA** | **Primary Score** | **Liver** | **Lung** | **Other** | **Any** | **Moribund** |
| **KPC** | | **(75.5)** | **(135)** | | | | | | | |
| 1 | F | - | 150 | Y | P | Y | Y | Y | Y | FD |
| 2 | M | 47 | 92 | Y | P | Y | N | Y | Y | Y |
| 3 | F | 98 | 140 | Y | M | N | N | N | N | N |
| 4 | M | 91 | 106 | Y | P | N | N | Y | Y | Y |
| 5 | F | - | 108 | Y | - | - | - | - | - | FD |
| 6 | F | - | 234 | Y | P | N | N | Y | Y | Y |
| 7 | F | 105 | 154 | Y | P | N | N | Y | Y | N |
| 8 | F | 73 | 80 | Y | P | Y | N | Y | Y | Y |
| 9 | F | 78 | 115 | Y | P | N | Y | Y | Y | FD |
| 10 | F | 98 | 155 | Y | - | - | - | - | - | FD |
| 11 | F | 91 | 135 | Y | P | N | Y | N | Y | N |
| 12 | M | 69 | 157 | Y | P | N | N | N | N | Y |
| 13 | M | 73 | 97 | N | PanIN | N | N | N | N | N |
| 14 | M | 54 | 203 | Y | W | Y | Y | Y | Y | Y |
| 15 | F | 69 | 88 | Y | - | Y | N | N | Y | FD |
| **KPC; Snail$^{F/+}$;Twist$^{F/+}$** | | **(88)** | **(153)** | | | | | | | |
| 16 | F | 142 | 153 | Y | W | N | N | N | N | N |
| 17 | M | 88 | 243 | Y | P | N | N | Y | Y | FD |
| 18 | F | 95 | 101 | Y | W | Y | N | N | Y | FD |
| 19 | M | 82 | 111 | Y | P | Y | Y | Y | Y | Y |
| 20 | F | 69 | 228 | Y | M | Y | N | Y | Y | Y |
| 21 | M | - | 163 | Y | - | - | - | - | - | FD |
| 22 | F | 77 | 127 | Y | - | - | - | - | - | FD |
| Totals | | | (Median) | 14/15 | | 5/13 | 4/13 | 8/13 | 10/13 | |
| % | | | | 93.3% | | 38.5% | 30.8% | 61.5% | 76.9% | |
| Totals with S$^{F/+}$T$^{F/+}$ | | | (Median) | 21/22 | | 8/18 | 6/18 | 11/18 | 14/18 | |
| % | | | | 95.5% | | 44.4% | 33.3% | 61.1% | 77.7% | |
| **KPC;ST** | | **(90)** | **(151)** | | | | | | | |
| 1 | M | - | 111 | Y | P | Y | Y | Y | Y | FD |
| 2 | F | 102 | 241 | Y | P | Y | Y | Y | Y | Y |
| 3 | F | 106 | 155 | Y | P | Y | N | Y | Y | N |
| 4 | F | 95 | 144 | Y | W | N | N | N | N | N |
| 5 | M | 85 | 134 | Y | W | N | N | N | N | N |
| 6 | F | 106 | 157 | Y | P | Y | N | Y | Y | Y |
| 7 | F | 120 | 157 | Y | P | N | N | N | N | N |
| 8 | F | - | 91 | Y | P | N | N | N | N | N |
| 9 | M | 86 | 113 | Y | P | N | N | N | N | FD |
| 10 | M | - | 130 | N | PanIN | N | N | N | N | N |

| | | | | | | | | | | |
|---|---|---|---|---|---|---|---|---|---|---|
| 11 | F | 57 | 85 | Y | W | N | N | N | N | N |
| 12 | M | 112 | 230 | Y | - | Y | - | - | - | N |
| 13 | M | 76 | 146 | Y | W | N | N | N | N | N |
| 14 | M | 69 | 146 | Y | P | Y | Y | Y | Y | Y |
| 15 | M | 67 | 198 | Y | M | Y | N | Y | Y | Y |
| 16 | M | 69 | 180 | Y | P | Y | Y | N | Y | FD |
| 17 | M | 85 | 104 | Y | P | N | N | N | N | FD |
| 18 | F | 124 | 230 | Y | P | Y | N | N | Y | Y |
| 19 | F | 116 | 185 | Y | P | Y | Y | Y | Y | Y |
| 20 | M | 82 | 132 | Y | - | - | - | - | - | N |
| 21 | F | 85 | 149 | Y | - | - | - | - | - | FD |
| 22 | M | 74 | 176 | Y | W | N | N | N | N | N |
| 23 | M | 60 | 157 | Y | P | N | N | N | N | Y |
| 24 | F | 138 | 195 | Y | M | Y | N | N | Y | N |
| 25 | M | 146 | 195 | Y | W | N | N | N | N | N |
| 26 | F | 54 | 146 | Y | P | N | N | Y | Y | Y |
| 27 | F | 109 | 132 | Y | W | N | N | N | N | N |
| 28 | F | 102 | 147 | Y | M | N | Y | N | Y | Y |
| 29 | F | 110 | 159 | Y | - | - | - | - | - | Y |
| 30 | M | 67 | 152 | Y | W | Y | Y | Y | Y | Y |
| 31 | F | 97 | 104 | Y | W | N | N | N | N | N |
| 32 | M | 90 | 212 | Y | M | Y | Y | N | Y | N |
| Totals | | | (Median) | 31/32 | | 13/29 | 8/29 | 8/29 | 14/29 | |
| % | | | | 96.9% | | 44.8% | 27.6% | 27.6% | 48.3% | |
| **KPPC** | | **(39)** | **(47)** | | | | | | | |
| 1 | F | - | 43 | Y | P | N | N | Y | Y | Y |
| 2 | M | 39 | 47 | Y | M | N | N | Y | Y | FD |
| 3 | M | 39 | 47 | Y | W | N | N | N | N | FD |
| 4 | M | 41 | 51 | Y | M | N | N | Y | Y | Y |
| Totals | | | (Median) | 4/4 | | 0/4 | 0/4 | 3/4 | 9/13 | |
| % | | | | 100.0% | | 0.0% | 0.0% | 75.0% | 69.2% | |
| **KPPC;Z $^{F/+}$** | | **(38)** | **(53.5)** | | | | | | | |
| 1 | M | 39 | 54 | Y | W | N | N | N | N | N |
| 2 | M | 27 | 53 | Y | P | N | N | N | N | N |
| 3 | M | 27 | 53 | Y | P | N | N | Y | Y | N |
| 4 | M | 27 | 53 | Y | P | N | N | Y | Y | Y |
| 5 | F | 27 | 53 | Y | W | N | N | Y | Y | Y |
| 6 | F | 35 | 60 | Y | W | N | N | Y | Y | N |
| 7 | M | 46 | 64 | Y | P | Y | N | Y | Y | Y |
| 8 | F | 37 | 73 | Y | W | N | N | Y | Y | Y |
| 9 | F | 41 | 41 | Y | M | N | N | N | N | Y |
| 10 | F | 41 | 51 | Y | W | Y | N | N | Y | Y |
| 11 | F | 41 | 66 | Y | P | N | N | N | N | Y |

| | | | | | | | | | | |
|---|---|---|---|---|---|---|---|---|---|---|
| 12 | M | 41 | 77 | Y | P | N | N | Y | Y | N |
| Totals | | | (Median) | 12/12 | | 2/12 | 0/12 | 7/12 | 8/12 | |
| % | | | | 100.0% | | 16.7% | 0.0% | 58.3% | 66.7% | |
| | | | | | | | | | | |
| **KPPC;Z$^{cKO}$** | | **(39)** | **(65.5)** | | | | | | | |
| 1 | F | 37 | 47 | Y | M | N | N | Y | Y | Y |
| 2 | M | 30 | 66 | Y | P | Y | N | Y | Y | Y |
| 3 | F | - | 35 | N | Normal/ PanIn | N | N | N | N | N |
| 4 | M | 55 | 65 | Y | P | N | N | Y | Y | Y |
| 5 | M | 39 | 90 | Y | W | Y | Y | Y | Y | Y |
| 6 | M | 55 | 80 | Y | W | N | N | N | N | Y |
| Totals | | (Median) | | 5/6 | | 2/5 | 1/5 | 4/5 | 4/5 | |
| % | | | | 83.3% | | 40.0% | 20.0% | 80.0% | 80.0% | |

Key: (Y) yes. (N) no, (W) well, (M) moderate, (P) poor, (FD) found dead, (-) no tissue

**Table S3.**
**Disease correlations between murine cohorts, Related to Figure 3 and 6.**

| Group | Parameter | *P* value or ^Chi-square | |
|---|---|---|---|
| **Microscopic Metastasis** | | **all** | **Endpoint** |
| Control vs. $Snail^{cKO}$;$Twist^{cKO}$ | Sex | 0.581 | 1.000 |
| KPPC vs. KPPC;$Z^{F/+}$ vs. KPPC;$Z^{cKO}$ | | ^0.615 | ^0.143 |
| Control vs. $Snail^{cKO}$;$Twist^{cKO}$ | Liver Metastasis | 1.000 | 1.000 |
| KPPC vs. KPPC;$Z^{F/+}$ vs. KPPC;$Z^{cKO}$ | | ^0.400 | ^0.371 |
| Control vs. $Snail^{cKO}$;$Twist^{cKO}$ | Lung Metastasis | 1.000 | 0.440 |
| KPPC vs. KPPC;$Z^{F/+}$ vs. KPPC;$Z^{cKO}$ | | ^0.247 | ^0.309 |
| Control vs. $Snail^{cKO}$;$Twist^{cKO}$ | Other Metastasis | 0.072 | 0.420 |
| KPPC vs. KPPC;$Z^{F/+}$ vs. KPPC;$Z^{cKO}$ | | ^0.822 | ^0.668 |
| Control vs. $Snail^{cKO}$;$Twist^{cKO}$ | Any Metastasis | 0.072 | 0.596 |
| KPPC vs. KPPC;$Z^{F/+}$ vs. KPPC;$Z^{cKO}$ | | ^0.949 | ^0944 |
| **Macroscopic Metastasis** | | **all** | **Endpoint** |
| Control vs. $Snail^{cKO}$;$Twist^{cKO}$ | Liver Metastasis | 0.762 | 0.710 |
| KPPC vs. KPPC;$Z^{F/+}$ vs. KPPC;$Z^{cKO}$ | | **^0.010** | **^0.017** |
| Control vs. $Snail^{cKO}$;$Twist^{cKO}$ | Lung Metastasis | 0.267 | 0.483 |
| KPPC vs. KPPC;$Z^{F/+}$ vs. KPPC;$Z^{cKO}$ | | na (all neg) | na (all neg) |
| Control vs. $Snail^{cKO}$;$Twist^{cKO}$ | Any Metastasis | 0.316 | 0.169 |
| KPPC vs. KPPC;$Z^{F/+}$ vs. KPPC;$Z^{cKO}$ | | ^0.615 | ^0.355 |
| Control vs. $Snail^{cKO}$;$Twist^{cKO}$ | Ascites | 1.000 | 0.700 |
| KPPC vs. KPPC;$Z^{F/+}$ vs. KPPC;$Z^{cKO}$ | | ^0.095 | ^0.202 |

***P*< 0.05**

Table S4: Antibodies, Related to Figures 3,5 and 6.

| Antigen | Antigen Retrieval | Blocking | Primary antibody Concentration | Primary antibody Catalog Number | Secondary antibody | Secondary antibody Concentration | Secondary antibody Company | Opal fluorophore Dye | Opal fluorophore Concentration |
|---|---|---|---|---|---|---|---|---|---|
| Slug | Citrate Buffer 15min Microwave | 4% CWFG | 1:200 | cs9585 | Rabbit on Rodent Polymer | stock | BioCare | Opal 540 | 1:100 |
| FSP1 | Citrate Buffer 15min Microwave | 1% BSA | 1:6,00 | A5114 | Rabbit on Rodent Polymer | stock | BioCare | Opal 570 | 1:2,000 |
| Zeb1 | Citrate Buffer 30min Microwave | 1% BSA | 1:2,000 | NBP1-05987 | Rabbit on Rodent Polymer | stock | BioCare | Opal 520 | 1:50 |
| Cytokeratin 8 | Citrate Buffer 45min Microwave | 1% BSA | 1:50 | TROMA-1 | Po-link Rat Polymer | stock | GBI Labs | Opal 690 | 1:200 |
| YFP | Citrate Buffer 60min Microwave | 4% CWFG | 1:30,000 | GFP-1020 | Po-link Chicken Polymer | stock | GBI Labs | Opal 650 | 1:1,000 |
| Twist1 | Citrate Buffer 75min Microwave | MOM Kit blocking - Vector Labs | 1:200 | NBP2-37364 | Mouse on Mouse Polymer | stock | BioCare | Opal 620 | 1:100 |
| Vimentin | Citrate Buffer 75min Microwave | 1% BSA | 1:1,000 | cs5741 | Rabbit on Rodent Polymer | stock | BioCare | Opal 520 | 1:50 |
| Snail/Slug | TrisEDTA 1hour pressure cooker | 4% CWFG | 1:20,000 | ab180714 | Rabbit on Rodent Polymer | stock | BioCare | Opal 570 | 1:1,000 |
| aSMA | Citrate Buffer 90min Microwave | 1% BSA | 1:500 | M0851 | Mouse on Mouse Polymer | stock | BioCare | Coumarin | 1:50 |
| E-cadherin | | | 1:100 | CS3195S | Goat-antiRabbit AF-488 | 1:500 | Invitrogen | | |
| YFP | Citrate Buffer 15min Microwave | 1% BSA | 1:500 | GFP1020 | Goat-antiChicken AF-594 | 1:1,000 | Invitrogen | NA | NA |
| Cytokeratin 8 | | | 1:50 | TROMA-1 | Goat-antiRat AF-647 | 1:1,000 | Invitrogen | | |
| E-cadherin | Citrate Buffer 15min Microwave | 1% BSA | 1:100 | CS3195S | Goat-antiRabbit Biotin | 1:250 | Vector Labs | Vector Labs ABC kit | |
| Ki67 | Citrate Buffer 1hour Microwave | 4% CWFG | 1:400 | Thermo RM9106S1 | Goat-antiRabbit Biotin | 1:250 | Vector Labs | Vector Labs ABC kit | |
| Zeb1 | Citrate Buffer 15min Microwave | 5% Goat Serum | 1:500 | NBP1-05987 | Goat-antiRabbit Biotin | 1:250 | Vector Labs | Vector Labs ABC kit | |
| CD8 | Citrate Buffer 15min Microwave | 10% Goat Serum | 1:500 | Biorbyt orb10325 | Rabbit on Rodent Polymer | stock | BioCare | Opal 540 | 1:100 |
| CD4 | Citrate Buffer 30min Microwave | 0.1% Triton, 1% goat serum, 1% BSA | 1:10,000 | SinoBiolo 50134-R001 | Rabbit on Rodent Polymer | stock | BioCare | Opal 650 | 1:1000 |
| Collagen I | Citrate Buffer 45min Microwave | 1% BSA | 1:500 | AbDSerotec 131001 | Po-link Goat Polymer | stock | GBI Labs | Coumarin | 1:50 |
| Cytokeratin 8 | Citrate Buffer 60min Microwave | 1% BSA | 1:50 | TROMA-1 | Po-link Rat Polymer | stock | GBI Labs | Opal 520 | 1:500 |
| aSMA | Citrate Buffer 75min Microwave | 1% BSA | 1:2,000 | DAKO M0851 | Mouse on Mouse Polymer | stock | BioCare | Opal 690 | 1:50 |
| CD3 | Citrate Buffer 90min Microwave | 1% BSA | 1:1,000 | DAKO A0452 | Rabbit on Rodent Polymer | stock | BioCare | Opal 620 | 1:500 |
| Foxp3 | Citrate Buffer 105min Microwave | 1% BSA | 1:2,000 | eBioscience 14-4771-80 | Po-link Rat Polymer | stock | GBI Labs | Opal 570 | 1:100 |
