# Supplementary material for: Stabilized epithelial phenotype of cancer cells in primary tumors leads to increased colonization of liver metastasis in pancreatic cancer
Source: Cell Rep. Author manuscript; Available in PMC 2021 Apr 27. (PMC8078733; doi:10.1016/j.celrep.2021.108990)
Supplement: 2 [file NIHMS1693705-supplement-2.pdf]

Cell Reports

Article

Stabilized epithelial phenotype of cancer cells in primary tumors leads to increased colonization of liver metastasis in pancreatic cancer

Graphical abstract

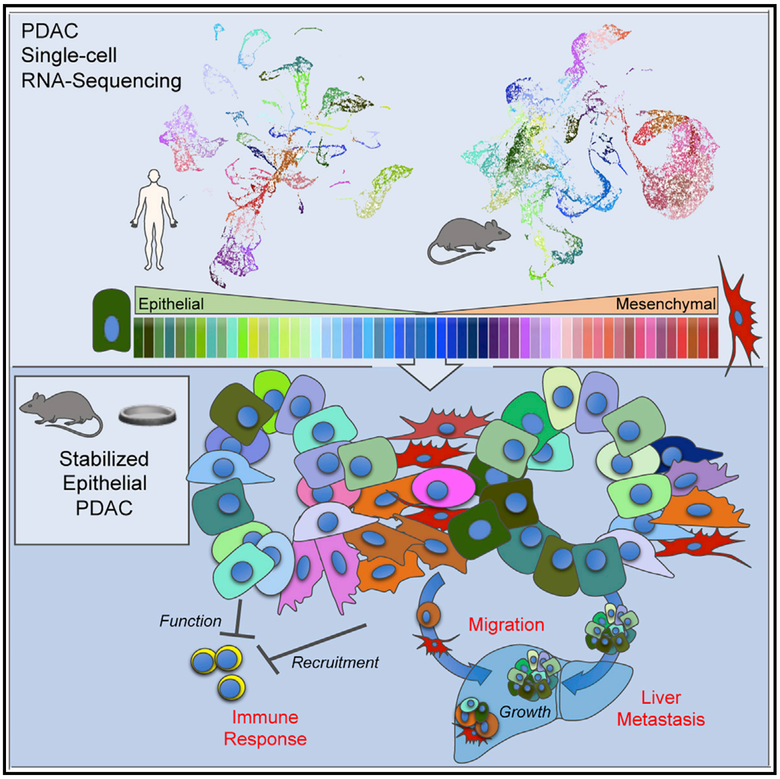

Highlights

- Single-cell RNA-seq reveals a continuum of cancer epithelial-mesenchymal transition
- Genetic deletion of both *Snai1* and *Twist1* or *Zeb1* stabilizes epithelial phenotypes
- Epithelial cancer cells have increased liver metastasis and collectively migrate
- Mesenchymal-like cancer cells recruit fewer T cells

Authors

Julienne L. Carstens, Sujuan Yang, Pedro Correa de Sampaio, ..., Jared K. Burks, Andrew D. Rhim, Raghu Kalluri

Correspondence

rkalluri@mdanderson.org

In brief

The necessity of partial epithelial to mesenchymal transition for cancer metastasis is unclear. Applying single-cell RNA sequencing and genetic models to stabilize the epithelial phenotype, Carstens et al. show a continuum of epithelial and mesenchymal pancreatic cancer cells that use different mechanisms to migrate, escape the immune system, and metastasize.

Carstens et al., 2021, Cell Reports *35*, 108990
April 13, 2021 © 2021 The Author(s).
https://doi.org/10.1016/j.celrep.2021.108990

CellPress

Cell Reports

CellPress
OPEN ACCESS

Article

Stabilized epithelial phenotype of cancer cells in primary tumors leads to increased colonization of liver metastasis in pancreatic cancer

Julienne L. Carstens,[1,8] Sujuan Yang,[1,8] Pedro Correa de Sampaio,[1] Xiaofeng Zheng,[1] Souptik Barua,[5] Kathleen M. McAndrews,[1] Arvind Rao,[4] Jared K. Burks,[2] Andrew D. Rhim,[3] and Raghu Kalluri[1,6,7,9,10,*]

[1]Department of Cancer Biology, Metastasis Research Center, University of Texas MD Anderson Cancer Center, Houston, TX 77054, USA
[2]Department of Leukemia, Division of Cancer Medicine, University of Texas MD Anderson Cancer Center, Houston, TX 77054, USA
[3]Department of Gastroenterology, Hepatology, and Nutrition, Division of Internal Medicine, University of Texas MD Anderson Cancer Center, Houston, TX 77054, USA
[4]Department of Computational Medicine and Bioinformatics, Biostatistics, Radiation Oncology, University of Michigan, Ann Arbor, MI 48105, USA
[5]Department of Electrical and Computer Engineering, Rice University, Houston, TX 77030, USA
[6]Department of Bioengineering, Rice University, Houston, TX 77030, USA
[7]Department of Molecular and Cellular Biology, Baylor College of Medicine, Houston, TX 77030, USA
[8]These authors contributed equally
[9]Senior author
[10]Lead contact
*Correspondence: rkalluri@mdanderson.org
https://doi.org/10.1016/j.celrep.2021.108990

SUMMARY

Pancreatic ductal adenocarcinoma (PDAC) is therapeutically recalcitrant and metastatic. Partial epithelial to mesenchymal transition (EMT) is associated with metastasis; however, a causal connection needs further unraveling. Here, we use single-cell RNA sequencing and genetic mouse models to identify the functional roles of partial EMT and epithelial stabilization in PDAC growth and metastasis. A global EMT expression signature identifies ~50 cancer cell clusters spanning the epithelial-mesenchymal continuum in both human and murine PDACs. The combined genetic suppression of Snail and Twist results in PDAC epithelial stabilization and increased liver metastasis. Genetic deletion of Zeb1 in PDAC cells also leads to liver metastasis associated with cancer cell epithelial stabilization. We demonstrate that epithelial stabilization leads to the enhanced collective migration of cancer cells and modulation of the immune microenvironment, which likely contribute to efficient liver colonization. Our study provides insights into the diverse mechanisms of metastasis in pancreatic cancer and potential therapeutic targets.

INTRODUCTION

Pancreatic ductal adenocarcinoma (PDAC) is a treatment-resistant, deadly disease with a 5-year survival rate of 9% and metastatic disease emerging in nearly 98% of patients (Siegel et al., 2019; Hidalgo, 2010). Such sobering statistics strongly urge an in-depth mechanistic analysis of PDAC metastatic disease. The acquisition of mesenchymal features by epithelial cells, traditionally called an epithelial-to-mesenchymal transition (EMT), has been studied in multiple cancers and correlates with metastasis and therapy resistance (Gao et al., 2019; Williams et al., 2019). EMT is not a simple binary process, either epithelial or mesenchymal, but instead a plastic continuum of partial EMT (pEMT) states between the epithelial and mesenchymal poles (Jolly et al., 2018; Kalluri and Weinberg, 2009; Lovisa et al., 2015, 2016; Brabletz et al., 2018; Aiello et al., 2018; Williams et al., 2019; da Silva-Diz et al., 2018; Yang et al., 2020). Such pEMT cells have been suggested as being important in cancer metastasis and drug resistance (Yang et al., 2020). The role of EMT in therapy resistance has been validated by multiple studies, but evidence for its requirement for metastasis is still evolving (Brabletz et al., 2018; Dongre and Weinberg, 2019; Williams et al., 2019; Liu et al., 2014).

It is well documented that EMT occurs in PDAC and is associated with cancer cell migration. The evidence includes gene expression analysis, live imaging, and cytology using *in vitro* systems, transplant models, genetically engineered mouse models (GEMMs), and analysis of human samples (Arumugam et al., 2009; Rhim et al., 2012; Von Burstin et al., 2009; Özdemir et al., 2014; Zheng et al., 2015; Chen et al., 2018; Krebs et al., 2017; Beerling et al., 2016; Reichert et al., 2018; Wang et al., 2019). However, histopathological and gene expression analyses of human PDAC samples show that the majority of metastatic PDACs are epithelial, with minimal evidence of intermediate morphologies (McDonald et al., 2012; Liu et al., 2014; Hayashi et al., 2020). Such observations with human PDAC

This is an open access article under the CC BY license (http://creativecommons.org/licenses/by/4.0/).

samples were also recapitulated in studies with PDAC model systems (Liu et al., 2014; Zheng et al., 2015; Chen et al., 2018). Such studies suggest pEMT may not be critical for all forms of PDAC metastasis, which requires careful unraveling.

Previous studies demonstrated that the depletion of an EMT driver, either Snail or Twist, had no effect on PDAC metastasis in mice (Zheng et al., 2015). In mice lacking Zeb1, metastases were reduced in number, and the primary tumor and remaining metastatic nodules displayed a well-differentiated epithelial phenotype (Krebs et al., 2017). Furthermore, transplantation studies have shown that epithelial PDAC cells can migrate and metastasize, and the re-expression of the epithelial adherens junction protein, E-cadherin, in mesenchymal PDAC cells enhances their migratory ability (Liu et al., 2014). An alternative to EMT-mediated motility is collective epithelial cell migration (Berton et al., 2009; Friedl et al., 2012; Friedl and Wolf, 2010). This form of migration has been a hypothesized mechanism for the circulation of cancer cell clusters, which is associated with poor clinical outcomes (Aceto et al., 2014; Fabisiewicz and Grzybowska, 2017; Giuliano et al., 2018). These data support the notion that migration, invasion, and metastasis of cancer cells may adapt different mechanisms outside the classical pEMT paradigm. However, the quantitation of cancer cells along the pEMT spectrum and the impact on the metastasis of stabilized EMT states remains to be elucidated.

Here, we use single-cell RNA sequencing (scRNA-seq) analysis of epithelial, pEMT, and mesenchymal cancer cells in human and mouse PDACs to identify the entire spectrum of the EMT continuum. We further explored the impact of stabilized pEMT states on PDAC metastasis via the use of multiple GEMMs that exhibit a stabilized epithelial phenotype through the deletion of EMT-driving transcription factors (deletion of Snail and Twist together or deletion of Zeb1). We demonstrate that the stabilization of an epithelial phenotype in PDAC cells enhances liver metastasis. In addition, we provide evidence for collective migration and immune microenvironment modifications as possible processes for epithelial PDAC metastasis. Collectively, we demonstrate that metastasis in PDACs may involve diverse mechanisms.

RESULTS

Single-cell RNA-seq of human PDAC reveals 51 different cancer cell phenotypes across the EMT continuum

To examine the prevalence of different EMT states in human PDAC, we evaluated the single-cell RNA-seq (scRNA-seq) dataset of >57,000 individual cells from 27 PDAC patient primary tumors (Peng et al., 2019). The cancer cell clusters were identified from the total population based on an epithelial gene expression signature (Figures 1A and S1A). RNA expression values of all cancer cells were subjected to Markov affinity-based graph imputation of cells (MAGIC) smoothing and clustering (van Dijk et al., 2018) based on the 315 genes identified by Thiery and colleagues as representing a universal EMT gene signature (referred to here as the Thiery EMT signature) (Figure 1B) (Tan et al., 2014). The clustering clearly demonstrated 51 different clusters of EMT in this dataset (Figure 1B). The RNA expression values for epithelial genes, divided by mesenchymal genes, were used to generate an E/M score for each cluster to organize them across the EMT spectrum from epithelial to mesenchymal (Figures 1C, 1D, S1B, and S1C). The 51 clusters were further sub-grouped into epithelial, pEMT, or mesenchymal phenotypes based on mathematical cutoffs (see Method details; Figure 1D). Eighteen clusters (34.09%) were predominantly of an epithelial phenotype (clusters 1–18, referred to here as E1–E18), and 10 clusters (24.48%) were predominantly of a mesenchymal phenotype (clusters 42–51, referred to here as M1–M10) (Figure 1D). The majority of the cancer cells (41.44%) clustered into 23 different pEMT states (clusters 19–41, referred to here as P1–P23) (Figure 1D). Next, the scRNA-seq analysis was computed for each individual PDAC patient using the above-described E/M score. This analysis revealed that 18.52% of the patients exhibited a predominately epithelial phenotype, 66.67% of the patients showed mainly pEMT phenotypes, and 14.81% revealed a mesenchymal phenotype (Figures S1D–S1G; Table S1).

To evaluate the correlation between the E/M score and the survival of PDAC patients, we next queried The Cancer Genome Atlas (TCGA) pancreatic cancer database with the Thiery EMT signature. PDAC tumors were classified as either epithelial, pEMT, or mesenchymal, and compared to previously reported classifiers by Moffitt et al. (2015) (basal versus classical), Collisson et al. (2011) (classical versus quasimesenchymal), and Chan-Seng-Yue et al. (2020) (basal A/B versus classical A/B), using the University of California, Santa Cruz (UCSC) Xena browser. In the TCGA cohort, which comprises early-stage/resectable tumors, none of the classifiers correlated with a survival advantage (Figure S1H). Despite this outcome in the TCGA cohort, we did observe a significant correlation between the Thiery classifier and the Collisson et al. (2011) (classical versus quasimesenchymal) and Chan-Seng-Yue et al. (2020) (basal A/B versus classical A/B) classifiers, which have been previously shown to predict survival (Table S1). These data show that the EMT program, demonstrated by the Thiery EMT signature, correlates with previously reported classifiers that suggest poorer patient outcome in the advanced tumor setting.

scRNA-seq of murine PDAC identifies 56 cancer cell phenotypes across the EMT spectrum

To further evaluate the EMT spectrum in murine PDAC, we conducted scRNA-seq of Pdx1-Cre; Kras$^{LSL-G12D}$; p53$^{R172H/+}$ (KPC) mice that develop clinically relevant primary and spontaneously metastasizing PDAC. Previous studies demonstrated a significant reduction in EMT upon the complete deletion of EMT-driving transcription factors Snail or Twist (Zheng et al., 2015). However, it remained unclear whether such manipulation affected the full spectrum of EMT states, or led to an incomplete EMT inhibition, or led to a compensation via the upregulation of other pEMT states not governed by either Snail or Twist (Brabletz et al., 2018).

To mechanistically address this issue, we generated a cancer cell-specific, dual deletion of both *Snai1* and *Twist1* by crossing Snail^{cKO}; YFP^{LSL} mice with KPC; Twist^{cKO}; YFP^{LSL} mice (KPC;ST) (Zheng et al., 2015). PDAC tumors from KPC and KPC;ST mice were subjected to scRNA-seq. Similar to the scRNA-seq analysis of human PDAC, murine cancer cells were clustered based on the expression of epithelial gene transcripts,

CellPress
OPEN ACCESS

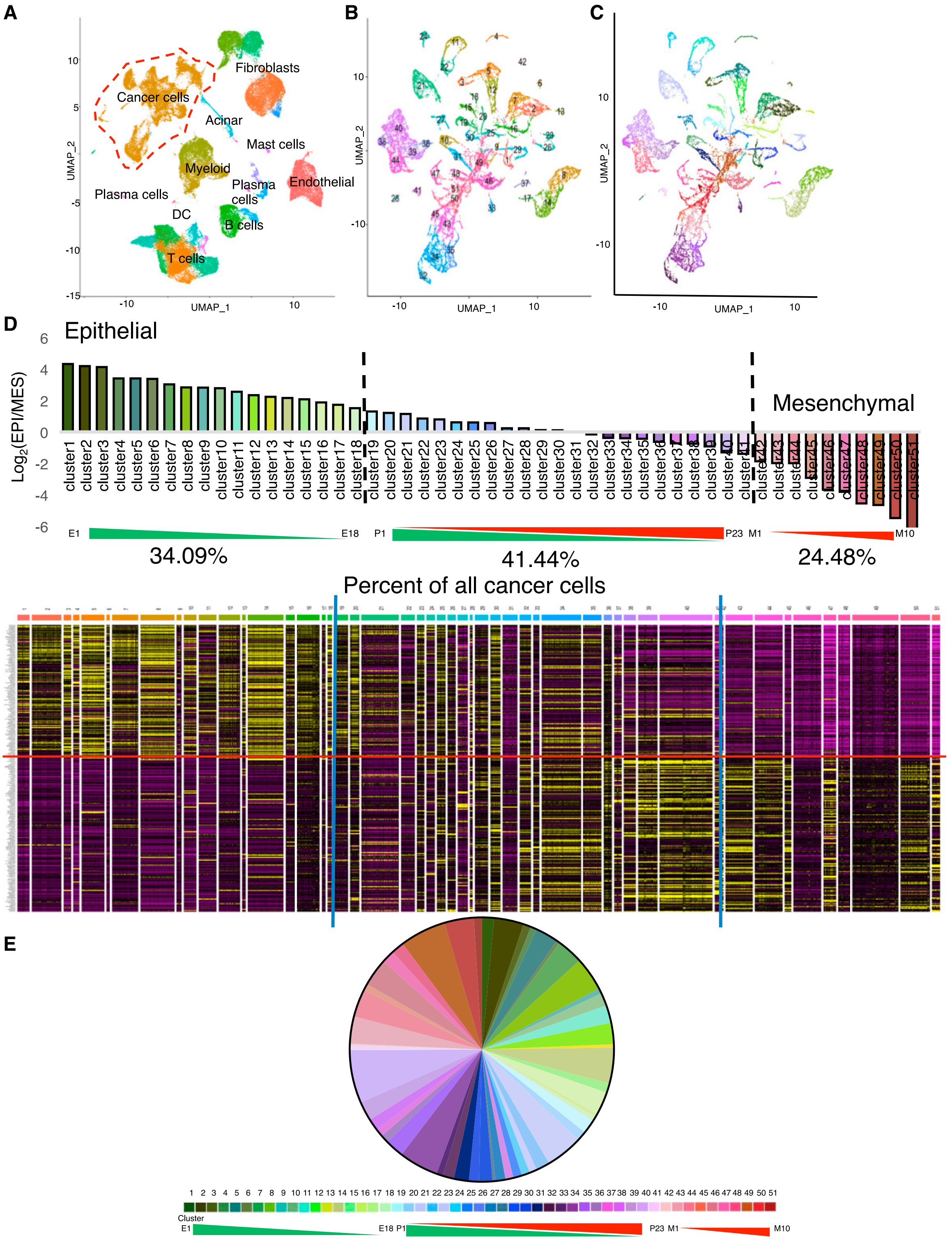

Figure 1. Single-cell RNA sequencing (RNA-seq) of human PDAC reveals 51 different cancer cell phenotypes across the EMT continuum

(A) Seurat uniform manifold approximation and projection (UMAP) clustering of whole-tissue single-cell populations. The red dashed line highlights the cancer cells isolated for further analysis.

(B) UMAP clustering of patient cancer cells post-MAGIC using the Thiery EMT signature.

(C) Same as (B), recolored to reflect the E/M score.

(legend continued on next page)

coupled with the enrichment of captured cancer cells via the engineered YFP (yellow fluorescent protein) signal (Figures 2A and S2A). Using MAGIC smoothing and clustering algorithms (van Dijk et al., 2018) of cancer cells based on the mouse correlates of the 315 genes of the Thiery EMT signature (294 genes) (Tan et al., 2014) followed by the generation of E/M scores, we identified 56 clusters encompassing the EMT spectrum (Figures 2B–2D and S2B–S2E). The mesenchymal clusters were largely positive for the canonical EMT transcription factors with overlapping and unique populations (Figure S2F). We also confirmed our Theiry EMT signature analyses with a principal-component analysis (PCA) of the MAGIC algorithms (Figures S2G–S2J). Overall, the spectrum of phenotypes exhibited in the aggregated mouse tumors are similar to those observed in the human PDAC analysis (Figures 2D and 2E). As in the human analyses, the 56 clusters were further sub-grouped into epithelial, pEMT, or mesenchymal phenotypes based on mathematical cutoffs (see Method details; Figure 2D). Eighteen clusters (34.45%) were predominantly of an epithelial phenotype (E1–E18), 18 clusters (23.92%) were predominantly pEMT states (clusters 19–36, referred to here as P1–P18), and 20 clusters (41.62%) were predominantly of a mesenchymal phenotype (clusters 37–56, referred to here as M1–M20) (Figure 2D). The KPC PDAC cells predominately exhibited a mesenchymal phenotype (Figures 2E and S2B–S2J). The loss of both Snail and Twist in the KPC;ST mice significantly stabilized the epithelial phenotype of cancer cells with more representation of clusters on the epithelial end of the EMT spectrum (Figures 2E and S2B–S2J), proving that Snail and Twist can affect the entire spectrum of EMT in the cancer cells.

To further appreciate the emergence of cancer cells within the EMT spectrum in relation to disease progression in the KPC and KPC;ST mice, we next performed scRNA-seq of PDAC tumors from early- and late-stage PDAC. Early-stage KPC PDAC cells presented with predominantly epithelial cluster phenotypes (clusters 1–26) and progressed to overtly mesenchymal cluster phenotypes in the late-stage KPC PDAC tumors (clusters 38–56) (Figure 2E). While the early KPC;ST PDAC cells were comparable to the early KPC PDAC cells in showing predominantly epithelial cluster phenotypes (clusters 1–26), the late-stage KPC;ST PDAC cells significantly maintained/stabilized epithelial cluster phenotypes (clusters 1–26) due to the loss of EMT-promoting Snail and Twist in the cancer cells (Figures 2E and S2B–S2F). Trajectory and pseudotime analyses indicate that the early-stage cancer cells are similar between the models and then progress through late-stage epithelial phenotypes with branches that later evolve into pEMT and mesenchymal types (Figures S2K–S2M). This pattern of cancer cell evolution is concordant with recent studies of whole exon sequencing in patients (Hayashi et al., 2020). The scRNA-seq data demonstrate that the KPC PDAC tumors exhibit multiple states within the EMT spectrum as observed in human PDAC. A significant impact on the EMT spectrum and the suppression of pEMT and mesenchymal phenotypes can be observed, with a combined deletion of Snail and Twist, using the Thiery EMT signature of 294 murine gene transcripts. This clearly suggests that Snail and Twist can regulate multiple signaling pathways to influence epithelial plasticity and the EMT program, and compensatory mechanisms are not realized when they are concurrently deleted, highlighting their rate-limiting role in this regard.

Stabilized epithelial phenotype of PDAC cells enhances liver metastasis

We next evaluated the impact of a stabilized epithelial phenotype in the KPC;ST mice on PDAC progression and metastasis. The combined loss of Snail and Twist had no impact on overall survival (Figure 3A; Table S2), disease-free survival (Figure 3B), and primary tumor burden (Figure 3C). Primary tumor histology was unchanged between the groups (Figures 3D and 3E). The gene expression analysis of EMT markers *Zeb1*, *Vimentin*, and *Col1a1* were significantly decreased in YFP sorted primary pancreatic cancer cell lines from KPC;ST mice, along with complete loss of *Snai1* and *Twist1* (Figure S3A). There was no significant difference in the gene expression of E-cadherin (*Cdh1*) but immunolabeling for YFP, E-cadherin (E-cad), and Cytokeratin 8 (CK8) protein expression showed a significant increase in the percentage of $YFP^{+}E\text{-}cad^{+}$ cells (Figures S3A and S3B). These data confirm previous reports that the protein expression of E-cadherin may not correlate with gene expression (Aiello et al., 2018). Furthermore, a significant decrease in the percentage of cells co-expressing YFP and Snail/Slug, Twist, Zeb1, vimentin, α smooth muscle actin (αSMA), or fibroblast-specific protein 1 (FSP1) was observed (Figures 3F and S3B). The Snail antibody used in this study binds both Snail and Slug (Snail2). Staining with an antibody specific for Slug revealed that Slug is non-nuclear, limited to the PanIN lesions, and did not decrease in the expression in KPC;ST mice (Figures 3F and S3B). These data show that a stabilized epithelial phenotype does not affect primary PDAC progression.

We next assessed the impact of the dual loss of Snail and Twist on PDAC metastasis. There was no change in the vascular dissemination of cancer cells, as measured by quantitating circulating YFP^{+} tumor cells (Figure S3C). Gross pathological assessment showed no difference in ascites or macroscopic metastatic nodules on the lung, liver, or other peritoneal organs (diaphragm, kidney/adrenal gland, gut, peritoneum, spleen, or reproductive organs) (Table S3). Microscopic analyses revealed insignificant differences in the incidence of metastatic disease (the number of mice per cohort with at least one lesion per specified organ; Table S3) or the number of metastatic lesions per organ (Figure 3G). However, the cumulative metastatic area in the liver was significantly larger in the KPC;ST mice (Figure 3H). Similarly, there were significantly more disseminated single YFP^{+} in the livers of KPC mice (Figure S3D). This was not due to an increased proliferation within the metastases, suggesting

(D) E/M score for each cluster, with the percentage of cancer cells in each E/M phenotype and corresponding expression heatmap of the Thiery EMT signature. Epithelial genes are above the red line. The blue line indicates the chosen cutoff between epithelial, partial, and mesenchymal groups.
(E) Percentage of cancer cells in each E/M phenotype.
See also Figure S1.

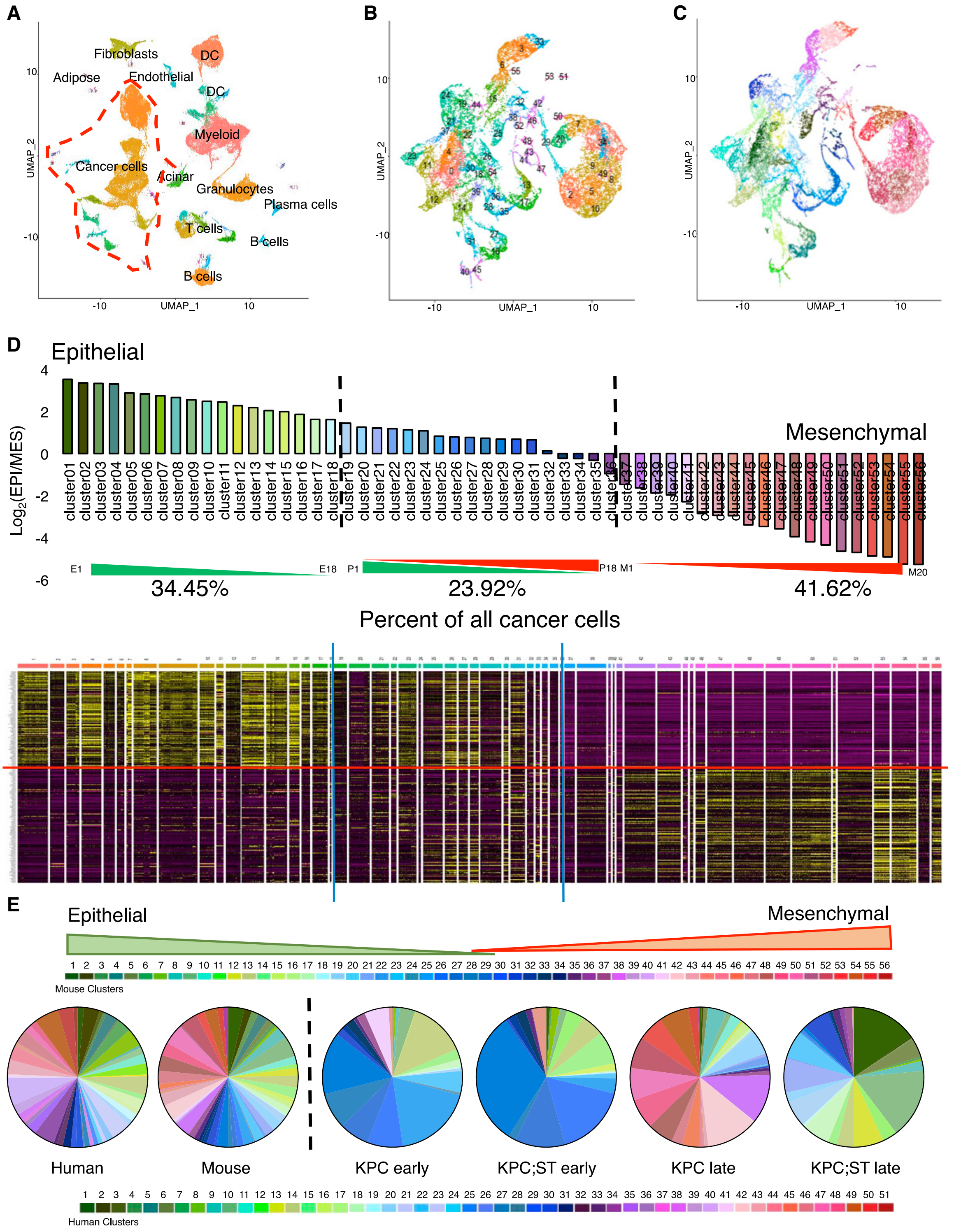

Figure 2. Single-cell RNA seq of murine PDAC identifies 56 cancer cell phenotypes across the EMT spectrum

(A) Seurat UMAP clustering of whole-tissue single-cell populations. The red dashed line highlights the cancer cells isolated for further analysis.

(B) UMAP clustering of murine pancreatic cancer cells post-MAGIC using the Thiery EMT signature.

(C) Same as (B), recolored to reflect the E/M score.

(D) E/M score for each cluster and corresponding expression heatmap of the Thiery EMT signature. Epithelial genes are above the red line. The blue line indicates the chosen cutoff between epithelial, partial, and mesenchymal groups, with the aggregated percentage of cancer cells indicated.

(E) Pie charts for the percentage of cancer cells in each E/M phenotype for aggregated human and mouse and each experimental mouse cohort.

See also Figure S2.

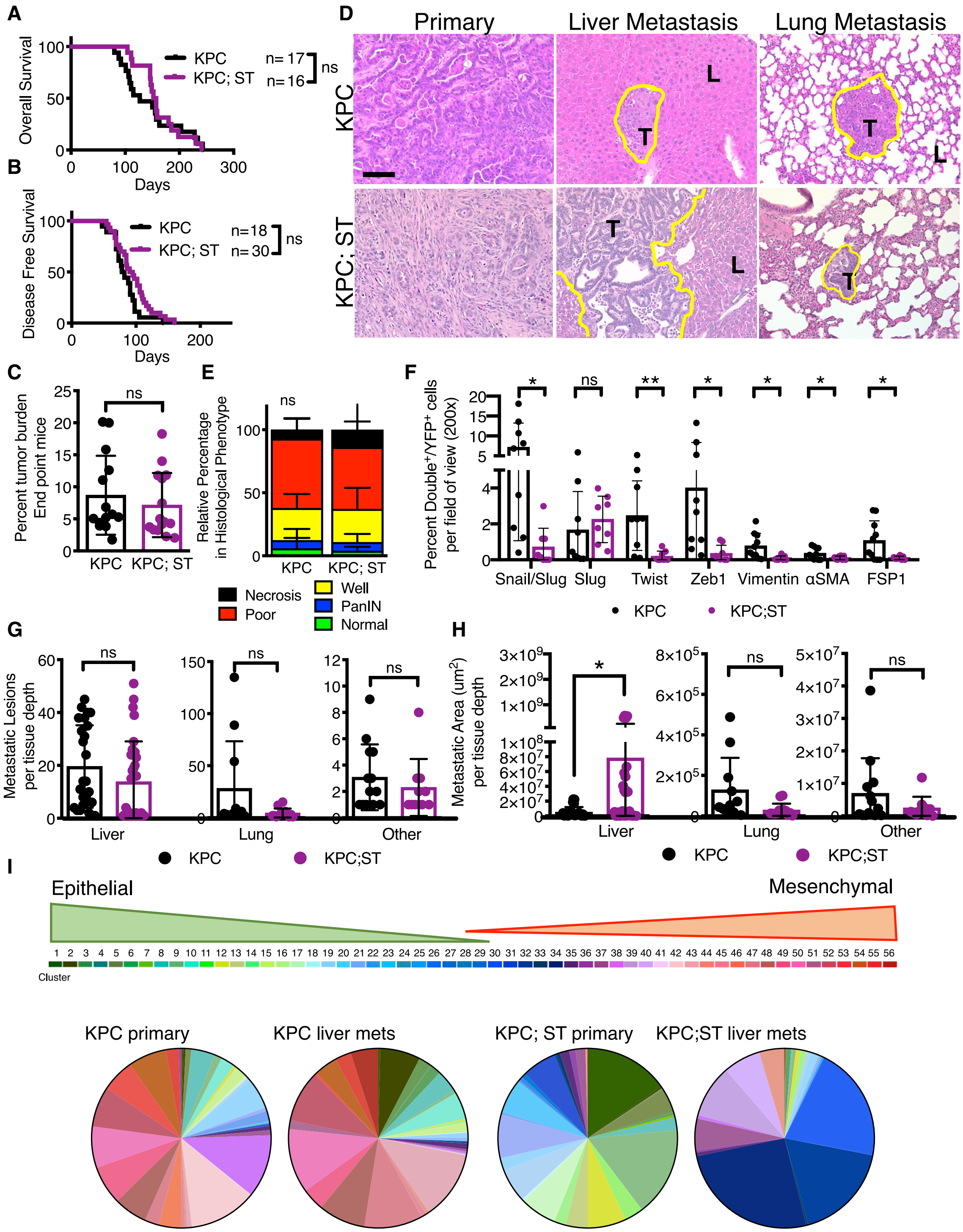

Figure 3. Stabilized epithelial phenotype of PDAC cells enhances liver metastasis

(A) Overall survival curve of KPC control (n = 17) and KPC;ST (n = 16) mice.

(B) Disease-free survival curve of KPC control mice (n = 18) and KPC;ST (n = 30) mice as determined by first palpable pancreatic nodule.

(C) Percentage of tumor burden (tumor-bearing pancreas weight/body weight) in grams of endpoint KPC control (n = 14) and KPC;ST (n = 15) mice.

(D) Representative histological micrographs (200×) stained with H&E of primary pancreatic tumors and liver and lung metastatic lesions; 100 μm scale bar. The yellow line outlines the border of metastatic lesions. T marks tumor area and L marks liver or lung, respectively.

(E) Relative percentage area of indicated epithelial phenotypes in primary pancreatic tumors of endpoint KPC control (n = 12) and KPC;ST (n = 14) mice. Two-way ANOVA.

(legend continued on next page)

CellPress
OPEN ACCESS

mechanisms outside of growth rate for the increase in metastatic liver colonization (Figure S3E).

Next, we performed scRNA-seq on cancer cells isolated from the livers of late-stage KPC and KPC;ST mice and compared it to the scRNA-seq of the corresponding primary tumors. The KPC PDAC primary and their corresponding liver metastatic cancer cells were indistinguishable in regard to the spectrum of E/M phenotypes that were identified, suggesting a potential capacity of each cluster from the primary tumor to metastasize or exhibit potential plasticity of a given cluster to expand into all primary tumor-associated clusters at the metastatic site (Figures 3I, S2B, and S2C). This is consistent with previous studies reporting similar mutation and expression profiles of primary and metastatic cancer cells in PDAC patients (Hayashi et al., 2020). However, the metastatic KPC;ST cancer cells isolated from the liver displayed an enrichment for unique E/M phenotypes not represented in KPC primary or metastatic tumors or the KPC;ST primary tumor (clusters 30, 32, and 34). Gene set enrichment analysis (GSEA) (Subramanian et al., 2005; Mootha et al., 2003) of the top expressing genes in these clusters showed an increase in membrane dynamics, lipid metabolism, and immune responses, notably interactions with platelets, coagulation, and complement (Figure S3F). This suggests that the stabilization of an epithelial phenotype in KPC;ST primary tumors may lead to unique metastatic adaptations, including modulation of the immune response. These data demonstrate that pancreatic cancer cells with a stabilized epithelial phenotype can efficiently metastasize, with an increased growth advantage in the liver.

Stabilized epithelial PDAC cells migrate by collective cell migration

We further examined the cancer cell-intrinsic pathways associated with stabilized epithelial PDAC cells. YFP^+ cancer cells were freshly sorted from the primary pancreatic tumors and the most common metastatic sites, liver and lung, of the KPC and KPC;ST mice for global expression profiling. GSEA of the KPC;ST primary tumors compared to the KPC control tumors showed 3,569 and 647 pathways up- and downregulated, respectively. The most highly upregulated pathways were associated with membrane dynamics, immune regulation, metabolism, and, notably, ameboid and epithelial migration (Figure 4A). Using Ingenuity Pathway Analysis (Krämer et al., 2014), we confirmed that the loss of Snail and Twist are critical nodes for regulating these pathways (Figures S4A and S4B). Similar pathway expression patterns were observed when examining metastatic cancer cells (3,616 and 600 pathways, respectively) (Figure 4B). Also, a common metastatic signature (3,152 and 1,064 pathways, respectively) between KPC;ST and KPC PDAC cells was noted (Figure S4C). String analysis of the core enriched genes in the ameboid migration gene set show RhoA as a central signaling hub (Szklarczyk et al., 2019). Using qPCR, we further confirmed the elevated expression of several genes in the epithelial/ameboid gene set (Figure S4D). These data suggest that the stabilization of epithelial PDAC in the primary tumor results in adaptive mechanisms that promote liver metastatic outgrowth.

To functionally validate the potential role of collective epithelial migration as a mechanism for metastasis in KPC;ST mice, we performed a scratch assay of YFP-sorted primary cancer cell lines. We used several primary cell lines isolated from KPC and KPC;ST tumors as well as a primary cell line isolated from an adenoviral-Cre-induced KPC; E-cadherin-KO (knockout) tumor to represent a stabilized mesenchymal phenotype. The proliferation rates, as determined by the frequency of passaging of the cell lines, were comparable between the groups. In general, both the KPC and KPC;ST lines maintained cell-cell contact in a uniform sheet with the absence of mesenchymal single cells (Figures 4C and 4D). However, unlike the ubiquitous epithelial morphology of the KPC;ST lines, the KPC lines were heterogeneous with some lines displaying leading cells with mesenchymal morphology (Figure S4E). The KPC; E-cadherin-KO cell line demonstrated pronounced single-cell mesenchymal morphology. Over the course of the scratch closure, both the KPC and the KPC;ST lines exhibited a steady decrease in single cells (particle counts) reflective of collective sheet migration (Figure 4E) (Friedl et al., 2012; Friedl and Wolf, 2010; Friedl et al., 2014). Alternatively, the KPC; E-cadherin-KO cell line maintained as single cells with clearly distinguishable boundaries (Figures 4C and 4D). The epithelial migration of the KPC and KPC;ST cells resulted in a more complete scratch closure than the single-cell mesenchymal migration of the KPC; E-cadherin-KO cell line (Figure 4F). Of note, the heterogeneity of the KPC lines also followed this pattern, with the line displaying the most collective phenotype (978U) clustering with the KPC;ST lines (Figures S4F and S4G). This suggests that the epithelial mode of migration is not due solely to the absence of Snail and Twist. These data confirm that epithelial PDAC cells migrate *in vitro* by collective sheet migration and that the loss of Snail and Twist does not prevent migration.

Epithelial PDAC cells associate with more T cells

To investigate the possibility of a distinct immune tumor microenvironment, we evaluated the number of infiltrating immune cells determined by scRNA-seq within KPC or KPC;ST tumors.

(F) Quantification of the percentage double positive of the indicated mesenchymal marker: Snail/Slug, Slug, Twist, Zeb1, Vimentin, αSMA, and FSP1 with YFP lineage tracing out the total number of YFP^+ cells per 200× image (5–10 images) per mouse: KPC (n = 9 or 10) and KPC;ST (n = 8) mice. Means ± SEMs.
(G) Quantification of the number of metastatic lesions per tissue depth in the liver (KPC control, n = 25 depths, 8 mice, and KPC;ST, n = 32 depths, 12 mice), lung (KPC control, n = 11 depths, 5 mice, and KPC;ST, n = 13 depths, 8 mice), and other tissues (KPC control, n = 13 depths, 11 mice, and KPC;ST, n = 10 depths, 9 mice).
(H) Quantification of the metastatic area per tissue depth for the liver (KPC control, n = 25 depths, 8 mice, and KPC;ST, n = 32 depths, 12 mice), lung (KPC control, n = 11 depths, 5 mice, and KPC;ST, n = 13 depths, 6 mice), and other tissues (KPC control, n = 13 depths, 11 mice, and KPC;ST mice, n = 10 depths, 9 mice).
(I) Pie charts for the percentage of cancer cells in each E/M phenotype across indicated experimental cohort. Unless otherwise specified, data are presented as means ± SDs and significance determined by an unpaired two-tailed t test. ns, not significant, *$p < 0.05$, **$p < 0.01$.
See also Figure S3.

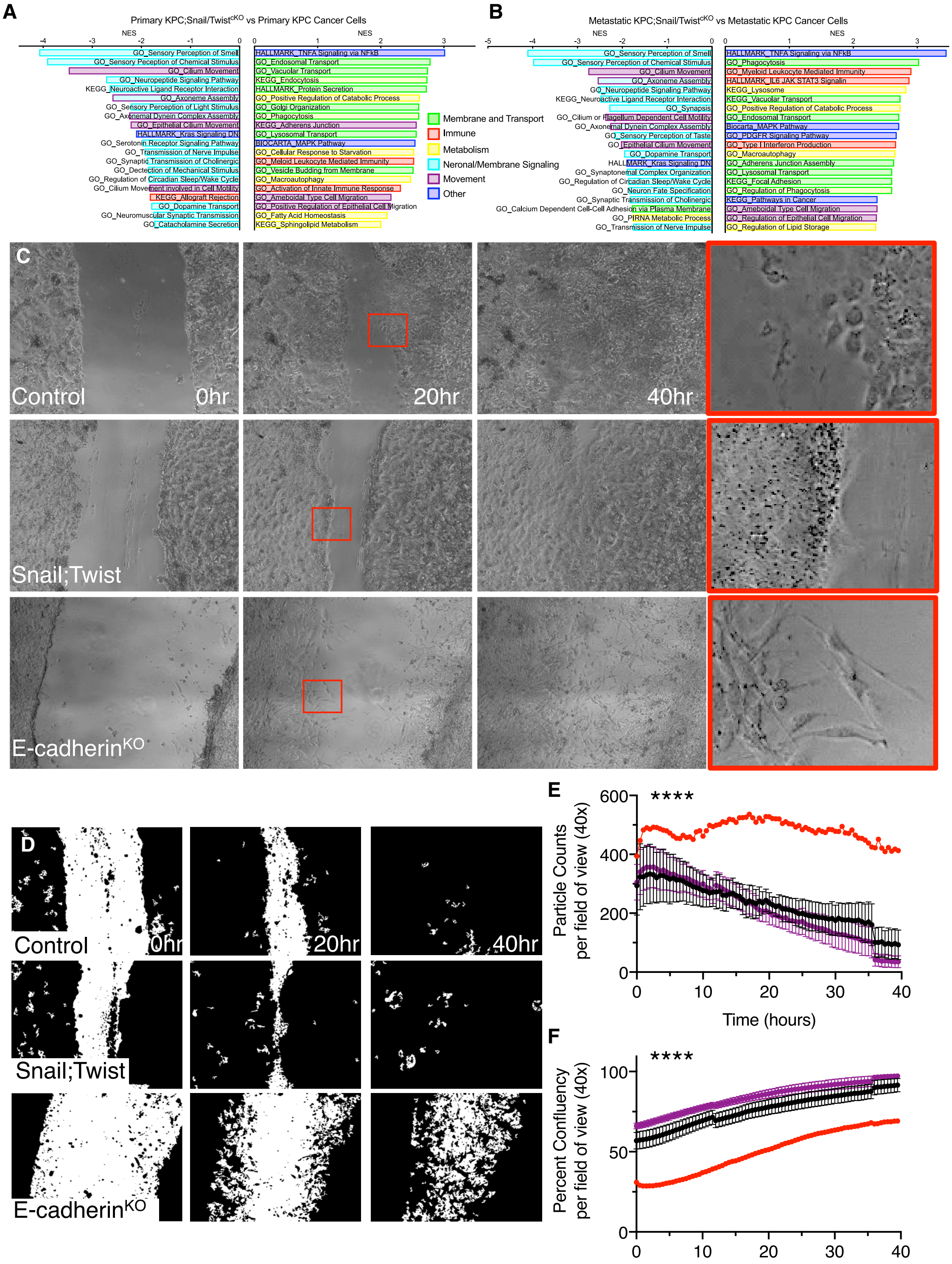

Figure 4. Stabilized epithelial PDAC cells migrate by collective cell migration

(A and B) Select enriched gene sets comparing primary tumors (A) and metastatic tumors (B). All pathways displayed are significant with a nominal p value, false discovery rate (FDR) q value, and family-wise error rate (FWER) p value < 0.05.

(legend continued on next page)

Both the early KPC and KPC;ST tumors had comparable levels of immune cells (Figure 5A). However, the late-stage KPC tumors displayed a dramatic reduction in the percentage of immune cells, while the KPC;ST tumors maintained a higher level of immune populations, notably T cells (Figure 5A). These data were confirmed using multiplex immunohistochemistry (Figure 5B), which showed a significant inverse correlation between $CD8^+$ T cells and EMT^+ cells ($CK8^+$ αSMA^+ and YFP^+ and any mesenchymal $marker^+$; see Figure 3) (Figures 5C and 5D). PDAC patient tumors' scRNA-seq showed no significant correlations in the percentages of infiltrating immune cells across the E/M spectrum tumors (Figures 5E and S5A). As tumor tissues are heterogeneous, we next investigated whether differences in immune infiltration could be observed in areas that contain more epithelial or mesenchymal cancer cells as opposed to bulk tumor counts. We have previously published the importance of the spatial localization of T cells in predicting clinical outcomes (Carstens et al., 2017). We re-examined our published tissue array for EMT ($CK8^+$ αSMA^+). As suggested by examining patient survival using the Thiery EMT signature, the presence of EMT alone did not predict survival (Figure 5F and 5G). However, the presence of EMT inversely correlated with T cells, which by themselves were a predictor of improved survival (Figures 5G, 5H, and S5B) (Carstens et al., 2017). We further determined that EMT^+ cancer cells had significantly fewer T cells within cell-cell contact distances (Figures 5I and S5C). These data demonstrate that epithelial and mesenchymal PDAC cells elicit differential effects on the infiltrating immune microenvironment.

Epithelial stablization via Zeb1 ablation also enhances liver metastasis

We show that the deletion of Snail and Twist is sufficient to suppress Zeb1 (Figure 3F). To specifically address the role of Zeb1-induced EMT in PDAC progression, we deleted Zeb1 in cancer cells of a second version of the KPC model with homozygous loss rather than a dominant negative mutation of p53 ($Kras^{G12D}$; $p53^{F/F}$; p48-Cre; YFP^{LSL}, hereafter referred to as KPPC). In comparison to the p53mut KPC model, the KPPC model displays a rapidly progressing primary tumor but with low distant metastases (Timpson et al., 2011). There was no delay in tumor onset in KPPC mice compared to KPPC with heterozygous Zeb1 deletion (KPPC;Z^{F+}) or homozygous Zeb1 deletion (KPPC;Z^{cKO}) as measured by the emergence of a first palpable pancreatic nodule (Figure 6A). The tumor onset kinetics agree with previously published KPC; $Zeb1^{cKO}$ data (Krebs et al., 2017). However, there was a significant delay in tumor progression as reflected in improved survival and a slower increase in tumor burden and histology over time, but with equivalent tumor burdens and histologies at endpoint (Figures 6B–6G). The reduction of cancer cell-specific Zeb1 protein expression was confirmed through immunolabeling of the primary tumor in both the KPPC;Z^{F+} and the KPPC; $Zeb1^{cKO}$ (Figures S6A and S6B) as well as quantified with the co-expression of mesenchymal markers and the lineage tracer YFP (Figure 6H). Similar to the dual loss of Snail and Twist, which reduce the cancer cell expression of Zeb1, we also observed the stabilization of an epithelial phenotype (Figure 6H). Next, we assessed the rate of metastasis. The tumors in the KPPC model are locally invasive to the surrounding gut, resulting in bowel obstruction and/or a blocked bile duct and jaundice, and were associated with a lower incidence of liver or lung metastases, when compared to the KPC mice (Table S2). We observed a significant increase in the rate of macroscopic liver metastasis in the KPPC; $Zeb1^{cKO}$ compared to KPPC (Tables S2 and S3). Microscopically, we observed a significant increase in the number and size of liver metastasis in both the KPPC; $Zeb1^{F+}$ and KPPC; $Zeb1^{cKO}$ (Figures 6G, 6I, and S6C; Tables S2 and S3). The increased liver metastasis trended with an increase proliferation, although not striking, again suggesting other mechanisms beyond proliferation as seen in the KPC;ST model (Figure S6D). These data confirm that the stabilization of the PDAC epithelial phenotype enhances liver metastasis even in a low metastatic KPPC PDAC model.

DISCUSSION

Recently, the importance of the EMT program in various biological processes, including cancer, and challenges in studying EMT were summarized with guidance for more rigorous *in vivo* genetic modeling studies (Yang et al., 2020). In this regard, advances in next-generation and single-cell sequencing technology provide an opportunity for a more nuanced analysis and appreciation of the full spectrum of the EMT continuum and addressing its role in metastasis.

Here, we provide single-cell quantitation of the EMT spectrum in human and KPC murine PDAC primary and metastatic tumors. Human and KPC PDAC displayed over 50 different phenotypic clusters of cancer cells across the EMT continuum based on the Thiery EMT signature using 315 human and 294 mouse gene transcripts. Our study clearly identifies that the EMT program of cancer cells is highly heterogeneous, with many cells existing in pEMT and mesenchymal states, along with different epithelial states. We discover that the EMT program is highly plastic and exists in an interchangeable continuum influenced substantially by transcription factors such as Snail, Twist, and Zeb1, which both overlap and represent unique subpopulations. We also identify that the genetic loss of Snail and Twist (associated with suppression of Zeb1) substantially affects the plastic continuum of the EMT program and stabilizes the epithelial phenotype with a shift toward epithelial cancer cells, away from the pEMT and mesenchymal states. These results suggest that a compensation for the loss of Snail and Twist was not encountered in this model system and that the EMT program, as represented by hundreds of genes, was affected at a broad

(C–F) Representative micrographs (40×) (C), matching inverted PHANTAST cell segmentation masks (D) and quantifications (E and F) of 3–4 replicate experiments with increasing passage numbers of cancer cell lines isolated from the primary tumors of KPC control, KPC;ST, and KP-E^{FF}-AdCre (n = 3, 3, and 1 line isolated from individual mice, respectively) plated to confluency. The red boxes indicate digital zoom of areas magnified in far-right panel. Quantifications of the particle counts (E) and percentage of cellular confluency (F) with linear regressions (****$p < 0.001$) of the grouped values post-scratch for the indicated time points. See also Figure S4.

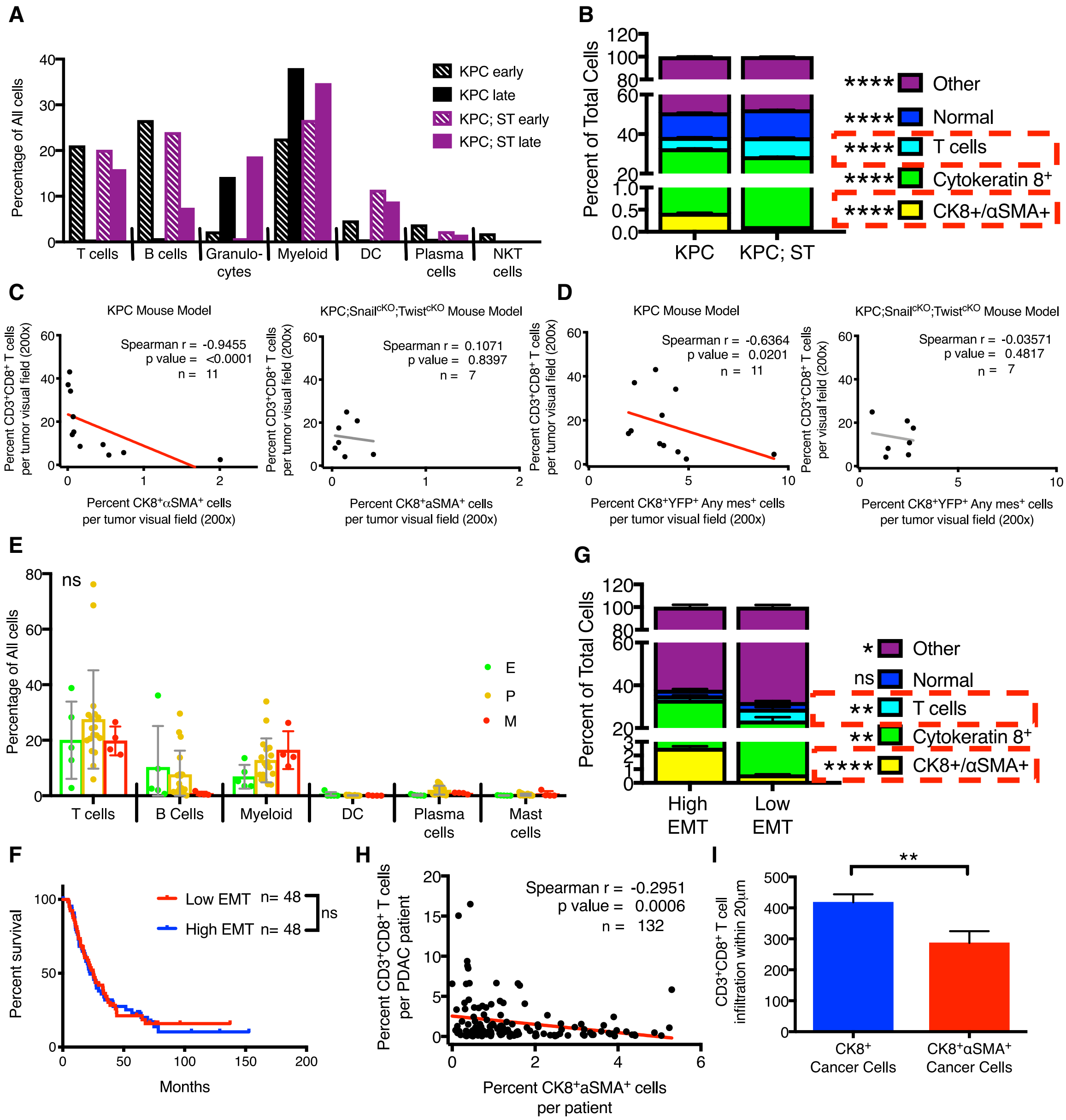

Figure 5. Epithelial PDAC cells associate with more T cells

(A) Immune cell percentages from scRNA-seq for each GEMM cohort.
(B) Cell population percentages from multiplexed immunohistochemistry for indicated genotype.
(C) Correlation between $CD8^+$ T cells and $CK8^+$ αSMA^+ cancer cells in KPC and KPC;ST tumors.
(D) Correlation between $CD8^+$ T cells and YFP^+CK8^+AnyMesenchymal $marker^+$ (Vimentin, αSMA, Zeb1, Snail, Twist, Slug, and FSP1) cancer cells in KPC and KPC;ST tumors.
(E) Immune cell percentages from scRNA-seq for each patient grouped by E/M classifications.
(F) Survival of patients high or low for EMT determined by multiplexed immunohistochemistry (split on the median).
(G) Cell population percentages from multiplexed immunohistochemistry for patients high or low for EMT ($CK8^+$ αSMA^+).
(H) Correlation between $CD8^+$ T cells and $CK8^+$ αSMA^+ cancer cells.
(I) L-function area under the curve values (reflecting the number of cells) for $CD8^+$ T cell within 20 μm of $CK8^+$ or $CK8^+$ αSMA^+ cancer cells; t test. Unless otherwise specified, data are presented as means ± SDs and significance determined by one-way ANOVA. ns, not significant, *$p < 0.05$, **$p < 0.01$, ****$p < 0.0001$.
See also Figure S5.

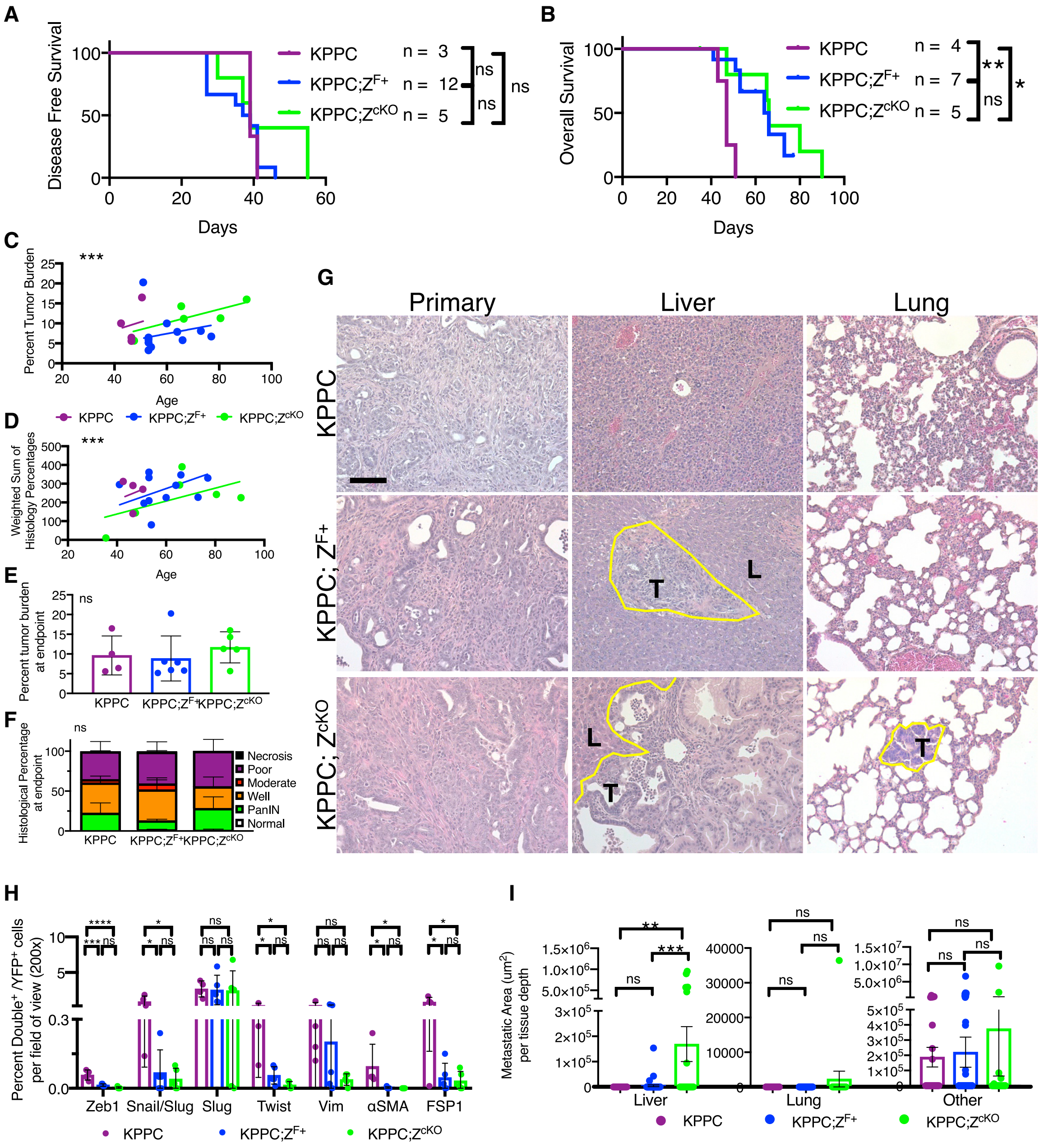

Figure 6. Epithelial stablization via Zeb1 ablation also enhances liver metastasis

(A) Disease-free survival curve of KPPC (n = 3), KPPC;$Z^{F/+}$ (n = 12), and KPPC; $Zeb1^{cKO}$(n = 5) mice as determined by first palpable pancreatic nodule.

(B) Overall survival of KPPC (n = 4), KPPC;$Z^{F/+}$ (n = 7), and KPPC; Z^{cKO}(n = 5) mice. Log-rank tests.

(C) Percentage of tumor burden (tumor-bearing pancreas weight/body weight) in grams plotted by age of necropsy (n = 4, 11, 5 mice, respectively); lines represent linear regression.

(D) Weighted sum of pathology scores for each mouse plotted by age of necropsy (n = 4, 7, and 5 mice); linear regression line.

(E) Percentage tumor burden of endpoint mice (n = 4, 6, 5 mice, respectively).

(F) Relative percentages of epithelial tumor histology of endpoint mice (n = 4, 7, 5 mice, respectively), two-way ANOVA.

(G) Representative histological micrographs (200×) stained with H&E of primary pancreatic tumors and liver and lung tissues. The yellow line outlines the border of metastatic lesions. T marks tumor area, and L marks liver or lung, respectively.

(H) Quantifications of the percentage of double positive out of YFP^+ cells per field of view (200× magnification) of primary pancreatic tumors (n = 4, 5, and 5 mice, respectively) and indicated mesenchymal markers: Snail/Slug, Slug, Twist, Zeb1, Vimentin, αSMA, or FSP1. SEM, ANOVA.

(legend continued on next page)

level, favoring an epithelial phenotype at the expense of pEMT and mesenchymal states. We further demonstrate that a large-scale inhibition of the EMT program does not impair metastasis, but rather is associated with increased liver metastasis, likely due to the fitness of the cancer cells with an epithelial phenotype. Importantly, the number of metastatic lesions does not change, suggesting that the extravasation and intravasation steps of cancer cell dissemination from the primary tumor are not impaired due to epithelial stabilization via inhibition of the EMT program, which is supported by previous studies (Zheng et al., 2015). These data suggest that inhibition of partial or hybrid EMT states does not impact metastasis; it appears that the system can compensate for the absence of mesenchymal features and accomplish metastasis with similar efficiency.

A previous study showed that the loss of Zeb1 in the cancer cells of KPC mice leads to decreased metastasis associated with a trend toward decreased primary tumor growth, and an insignificant difference in disease-free survival of the mice (Krebs et al., 2017). These results suggested that decreased metastasis could be a direct reflection of the rate of primary tumor progression (Krebs et al., 2017). To further validate that epithelial stability can lead to increased liver metastasis, we used a genetic mouse model of pancreatic cancer that is recalcitrant to metastasis (Timpson et al., 2011). This genetic mouse model substitutes the mutant p53 allele in the KPC mice with an allele leading to the loss of p53 ($p53^{F/F}$), and the resulting KPPC mice develop primary PDAC, but rarely metastasize. Deletion of Zeb1 in KPPC mice led to a decreased rate of primary tumor growth but an insignificant difference in disease-free survival of these mice when compared to the KPPC mice. The loss of Zeb1 enabled the emergence of significant liver metastasis associated with the epithelial stabilization of the EMT program. These results further support the idea (Reichert et al., 2018) that suppression of an EMT program leads to epithelial stabilization, resulting in the fitness of cancer cells to form efficient liver metastatic colonies.

The concept of epithelial versus mesenchymal cancer cells and its implications on clinical prognosis is well established in the PDAC field. Basal-like or quasi-mesenchymal gene expression subtypes have been associated with poor patient outcomes (Porter et al., 2019). In addition, model studies have provided a causative link between mesenchymal traits and therapeutic resistance (Shah et al., 2007; Arumugam et al., 2009; Wellner et al., 2009; Krebs et al., 2017; Gaianigo et al., 2017). However, patients with epithelial/classical phenotypes represent approximately two-thirds of the clinical population and still have poor prognosis with metastatic disease (Porter et al., 2019). Furthermore, 76%–80% of PDAC metastasizes to the liver, regardless of expression subtypes (Houg and Bijlsma, 2018; Hayashi et al., 2020). The data presented here are in agreement with these clinical observations and suggest a link between the presentation of classical/epithelial subtypes and the preponderance of liver metastasis.

In summary, our data suggest that the EMT program on a single-cell level within the primary tumor is heterogeneous, and cancer cells can exist within multiple states within the full spectrum of the EMT continuum. Suppression of this plastic EMT spectrum leads to epithelial stabilization and contributes to the fitness of the cancer cells for liver metastatic colonization, without any impact on the dissemination of the cancer cells. Our data suggest that the dissemination of epithelial cancer cells is facilitated by collective migration and collaboration with the immune system. Successful colonization and growth are considered to be the most challenging steps of metastatic disease, and the present study highlights the importance of epithelial stabilization in this process and offers insights into potential therapeutic interventions.

STAR★METHODS

Detailed methods are provided in the online version of this paper and include the following:

- KEY RESOURCES TABLE
- RESOURCE AVAILABILITY
  - ○ Lead contact
  - ○ Materials availability
  - ○ Data and code availability
- EXPERIMENTAL MODEL AND SUBJECT DETAILS
  - ○ Mouse studies
  - ○ Primary tissue sorting and cell line generation
- METHOD DETAILS
  - ○ Single-cell RNA-sequencing
  - ○ Histology and histopathology
  - ○ Immunohistochemical staining and analyses
  - ○ CTC assays
  - ○ Quantitative reverse-transcriptase PCR analyses (qPCR)
  - ○ Microarray analyses
  - ○ Migration assays
- QUANTIFICATION AND STATISTICAL ANALYSIS

SUPPLEMENTAL INFORMATION

Supplemental information can be found online at https://doi.org/10.1016/j.celrep.2021.108990.

ACKNOWLEDGMENTS

Special thanks to David Dwyer, Sarah Schneider, Ryan Jewell, Karen Ramirez, and Veena Papanna at the South Campus Flow Cytometry Core for their expertise in sorting endogenously labeled YFP^{+} cancer cells (NCI grant P30CA16672). Special thanks to Drs. Chang-gong Liu and Xiuping Liu of the MDACC Sequencing and ncRNA Program for their expertise in performing the microarrays. Special thanks to Erika J. Thompson, David P. Pollock, and Yunxin Chen for their expertise in performing the single-cell RNA sequencing (NIH P30CA016672). This study was primarily supported by the Cancer

(I) Quantification of the metastatic area per tissue depth for liver (KPPC, n = 16 depths, 4 mice, KPPC;$Z^{F/+}$, n = 48 depths, 12 mice, and KPPC; Z^{cKO}, n = 20 depths, 5 mice), lung (KPPC, n = 12 depths, 4 mice, KPPC;$Z^{F/+}$, n = 36 depths, 12 mice, and KPPC; Z^{cKO}, n = 15 depths, 5 mice), and other tissues (KPPC, n = 50 depths, 4 mice, KPPC;$Z^{F/+}$, n = 72 depths, 12 mice, and KPPC; Z^{cKO}, n = 30 depths, 5 mice). Unless otherwise specified, data are presented as means ± SDs, scale bar: 100 μm, and significance determined by a one-way ANOVA. ns, not significant, *$p < 0.05$, **$p < 0.01$, ***$p < 0.001$, ****$p < 0.0001$.
See also Figure S6.

Prevention and Research Institute of Texas and the Metastasis Research Center at the MD Anderson Cancer Center. The pancreatic cancer research in the R.K. laboratory is also supported by the NIH grant P01CA117969. This research was performed in the Flow Cytometry & Cellular Imaging Core Facility, which is supported in part by the NIH CA016672. J.K.B. is further supported by the NCI's Research Specialist 1 R50 CA243707-01A1. S.B. and A.R. were supported by CCSG P30 CA046592, NCI R37CA214955-01A1, a gift from Agilent Technologies, and a Research Scholar Grant from the American Cancer Society (RSG-16-005-01).

AUTHOR CONTRIBUTIONS

J.L.C. helped design the experimental strategy, performed experiments to generate the genetically engineered mouse models, analyzed the data related to the genetically engineered mouse models, performed culture experiments, collected data, helped with scRNA-seq sample collection and discussion, generated the figures, and helped write the manuscript. S.Y. helped generate the genetically engineered mouse models and helped conceptually design and perform the scRNA-seq experiments and analysis. P.C.d.S. helped with assay optimization, multiplex data analysis, sample collection, and manuscript editing. X.Z. helped generate the KPC;ST. S.B. generated the spatial analysis algorithms and data, developed the computer code for analyses, analyzed the data, and participated in discussions related to the spatial and survival analyses. K.M.M. helped with immunohistochemical staining and manuscript editing. A.R. conceived and designed the analytic strategy for spatial infiltration analysis, supervised S.B. toward the implementation of the analysis algorithms, provided intellectual input, and participated in discussions related to the spatial and survival analysis. J.K.B. helped conceive and design the analytic strategy for the Visiopharm analysis of the mouse immune multiplex, provided intellectual input, and participated in discussions related to the spatial analysis. A.D.R. provided the KPPC-Zeb1-flox mouse line. R.K. conceptually designed the strategy for this study, participated in discussions, provided intellectual input, supervised experimental discussion, and helped write the manuscript.

DECLARATION OF INTERESTS

The authors declare no competing interests.

Received: August 26, 2020
Revised: January 25, 2021
Accepted: March 23, 2021
Published: April 13, 2021

REFERENCES

Aceto, N., Bardia, A., Miyamoto, D.T., Donaldson, M.C., Wittner, B.S., Spencer, J.A., Yu, M., Pely, A., Engstrom, A., Zhu, H., et al. (2014). Circulating tumor cell clusters are oligoclonal precursors of breast cancer metastasis. Cell *158*, 1110–1122.

Aiello, N.M., Maddipati, R., Norgard, R.J., Balli, D., Li, J., Yuan, S., Yamazoe, T., Black, T., Sahmoud, A., Furth, E.E., et al. (2018). Emt subtype influences epithelial plasticity and mode of cell migration. Dev. Cell *45*, 681–695.e4.

Arumugam, T., Ramachandran, V., Fournier, K.F., Wang, H., Marquis, L., Abbruzzese, J.L., Gallick, G.E., Logsdon, C.D., McConkey, D.J., and Choi, W. (2009). Epithelial to mesenchymal transition contributes to drug resistance in pancreatic cancer. Cancer Res. *69*, 5820–5828.

Bankhead, P., Loughrey, M.B., Fernández, J.A., Dombrowski, Y., McArt, D.G., Dunne, P.D., McQuaid, S., Gray, R.T., Murray, L.J., Coleman, H.G., et al. (2017). QuPath: open source software for digital pathology image analysis. Sci. Rep. *7*, 16878.

Beerling, E., Oosterom, I., Voest, E., Lolkema, M., and van Rheenen, J. (2016). Intravital characterization of tumor cell migration in pancreatic cancer. Intravital *5*, e1261773.

Berton, S., Belletti, B., Wolf, K., Canzonieri, V., Lovat, F., Vecchione, A., Colombatti, A., Friedl, P., and Baldassarre, G. (2009). The tumor suppressor functions of p27(kip1) include control of the mesenchymal/amoeboid transition. Mol. Cell. Biol. *29*, 5031–5045.

Brabletz, T., Kalluri, R., Nieto, M.A., and Weinberg, R.A. (2018). EMT in cancer. Nat. Rev. Cancer *18*, 128–134.

Butler, A., Hoffman, P., Smibert, P., Papalexi, E., and Satija, R. (2018). Integrating single-cell transcriptomic data across different conditions, technologies, and species. Nat. Biotechnol. *36*, 411–420.

Carstens, J.L., Correa de Sampaio, P., Yang, D., Barua, S., Wang, H., Rao, A., Allison, J.P., LeBleu, V.S., and Kalluri, R. (2017). Spatial computation of intratumoral T cells correlates with survival of patients with pancreatic cancer. Nat. Commun. *8*, 15095.

Chan-Seng-Yue, M., Kim, J.C., Wilson, G.W., Ng, K., Figueroa, E.F., O'Kane, G.M., Connor, A.A., Denroche, R.E., Grant, R.C., McLeod, J., et al. (2020). Transcription phenotypes of pancreatic cancer are driven by genomic events during tumor evolution. Nat. Genet. *52*, 231–240.

Chen, Y., LeBleu, V.S., Carstens, J.L., Sugimoto, H., Zheng, X., Malasi, S., Saur, D., and Kalluri, R. (2018). Dual reporter genetic mouse models of pancreatic cancer identify an epithelial-to-mesenchymal transition-independent metastasis program. EMBO Mol. Med. *10*, e9085.

Collisson, E.A., Sadanandam, A., Olson, P., Gibb, W.J., Truitt, M., Gu, S., Cooc, J., Weinkle, J., Kim, G.E., Jakkula, L., et al. (2011). Subtypes of pancreatic ductal adenocarcinoma and their differing responses to therapy. Nat. Med. *17*, 500–503.

da Silva-Diz, V., Lorenzo-Sanz, L., Bernat-Peguera, A., Lopez-Cerda, M., and Muñoz, P. (2018). Cancer cell plasticity: impact on tumor progression and therapy response. Semin. Cancer Biol. *53*, 48–58.

Derksen, P.W., Liu, X., Saridin, F., van der Gulden, H., Zevenhoven, J., Evers, B., van Beijnum, J.R., Griffioen, A.W., Vink, J., Krimpenfort, P., et al. (2006). Somatic inactivation of E-cadherin and p53 in mice leads to metastatic lobular mammary carcinoma through induction of anoikis resistance and angiogenesis. Cancer Cell *10*, 437–449.

Dongre, A., and Weinberg, R.A. (2019). New insights into the mechanisms of epithelial-mesenchymal transition and implications for cancer. Nat. Rev. Mol. Cell Biol. *20*, 69–84.

Fabisiewicz, A., and Grzybowska, E. (2017). CTC clusters in cancer progression and metastasis. Med. Oncol. *34*, 12.

Friedl, P., and Wolf, K. (2010). Plasticity of cell migration: a multiscale tuning model. J. Cell Biol. *188*, 11–19.

Friedl, P., Locker, J., Sahai, E., and Segall, J.E. (2012). Classifying collective cancer cell invasion. Nat. Cell Biol. *14*, 777–783.

Friedl, P., Wolf, K., and Zegers, M.M. (2014). Rho-directed forces in collective migration. Nat. Cell Biol. *16*, 208–210.

Gaianigo, N., Melisi, D., and Carbone, C. (2017). Emt and treatment resistance in pancreatic cancer. Cancers (Basel) *9*, 122.

Gao, Y., Bado, I., Wang, H., Zhang, W., Rosen, J.M., and Zhang, X.H.F. (2019). Metastasis organotropism: redefining the congenial soil. Dev. Cell *49*, 375–391.

Gierut, J.J., Jacks, T.E., and Haigis, K.M. (2014). In vivo delivery of lenti-Cre or adeno-Cre into mice using intranasal instillation. Cold Spring Harb. Protoc. *2014*, 307–309.

Giuliano, M., Shaikh, A., Lo, H.C., Arpino, G., De Placido, S., Zhang, X.H., Cristofanilli, M., Schiff, R., and Trivedi, M.V. (2018). Perspective on circulating tumor cell clusters: why it takes a village to metastasize. Cancer Res. *78*, 845–852.

Goldman, M.J., Craft, B., Hastie, M., Repečka, K., McDade, F., Kamath, A., Banerjee, A., Luo, Y., Rogers, D., Brooks, A.N., et al. (2020). Visualizing and interpreting cancer genomics data via the Xena platform. Nat. Biotechnol. *38*, 675–678.

Guerra, C., and Barbacid, M. (2013). Genetically engineered mouse models of pancreatic adenocarcinoma. Mol. Oncol. *7*, 232–247.

Haghverdi, L., Lun, A.T.L., Morgan, M.D., and Marioni, J.C. (2018). Batch effects in single-cell RNA-sequencing data are corrected by matching mutual nearest neighbors. Nat. Biotechnol. *36*, 421–427.

Hayashi, A., Fan, J., Chen, R., Ho, Y.-J., Makohon-Moore, A.P., Lecomte, N., Zhong, Y., Hong, J., Huang, J., Sakamoto, H., et al. (2020). A unifying paradigm for transcriptional heterogeneity and squamous features in pancreatic ductal adenocarcinoma. Nat. Cancer *1*, 59–74.

Hidalgo, M. (2010). Pancreatic cancer. N. Engl. J. Med. *362*, 1605–1617.

Houg, D.S., and Bijlsma, M.F. (2018). The hepatic pre-metastatic niche in pancreatic ductal adenocarcinoma. Mol. Cancer *17*, 95.

Jaccard, N., Griffin, L.D., Keser, A., Macown, R.J., Super, A., Veraitch, F.S., and Szita, N. (2014). Automated method for the rapid and precise estimation of adherent cell culture characteristics from phase contrast microscopy images. Biotechnol. Bioeng. *111*, 504–517.

Jolly, M.K., Mani, S.A., and Levine, H. (2018). Hybrid epithelial/mesenchymal phenotype(s): The 'fittest' for metastasis? Biochim. Biophys. Acta Rev. Cancer *1870*, 151–157.

Kalluri, R., and Weinberg, R.A. (2009). The basics of epithelial-mesenchymal transition. J. Clin. Invest. *119*, 1420–1428.

Krämer, A., Green, J., Pollard, J., Jr., and Tugendreich, S. (2014). Causal analysis approaches in ingenuity pathway analysis. Bioinformatics *30*, 523–530.

Krebs, A.M., Mitschke, J., Lasierra Losada, M., Schmalhofer, O., Boerries, M., Busch, H., Boettcher, M., Mougiakakos, D., Reichardt, W., Bronsert, P., et al. (2017). The EMT-activator Zeb1 is a key factor for cell plasticity and promotes metastasis in pancreatic cancer. Nat. Cell Biol. *19*, 518–529.

Liu, X., Huang, H., Remmers, N., and Hollingsworth, M.A. (2014). Loss of E-cadherin and epithelial to mesenchymal transition is not required for cell motility in tissues or for metastasis. Tissue Barriers *2*, e969112.

Lovisa, S., LeBleu, V.S., Tampe, B., Sugimoto, H., Vadnagara, K., Carstens, J.L., Wu, C.C., Hagos, Y., Burckhardt, B.C., Pentcheva-Hoang, T., et al. (2015). Epithelial-to-mesenchymal transition induces cell cycle arrest and parenchymal damage in renal fibrosis. Nat. Med. *21*, 998–1009.

Lovisa, S., Zeisberg, M., and Kalluri, R. (2016). Partial epithelial-to-mesenchymal transition and other new mechanisms of kidney fibrosis. Trends Endocrinol. Metab. *27*, 681–695.

McDonald, O.G., Maitra, A., and Hruban, R.H. (2012). Human correlates of provocative questions in pancreatic pathology. Adv. Anat. Pathol. *19*, 351–362.

Moffitt, R.A., Marayati, R., Flate, E.L., Volmar, K.E., Loeza, S.G., Hoadley, K.A., Rashid, N.U., Williams, L.A., Eaton, S.C., Chung, A.H., et al. (2015). Virtual microdissection identifies distinct tumor- and stroma-specific subtypes of pancreatic ductal adenocarcinoma. Nat. Genet. *47*, 1168–1178.

Mootha, V.K., Lindgren, C.M., Eriksson, K.-F., Subramanian, A., Sihag, S., Lehar, J., Puigserver, P., Carlsson, E., Ridderstråle, M., Laurila, E., et al. (2003). PGC-1α-responsive genes involved in oxidative phosphorylation are coordinately downregulated in human diabetes. Nat. Genet. *34*, 267–273.

Özdemir, B.C., Pentcheva-Hoang, T., Carstens, J.L., Zheng, X., Wu, C.C., Simpson, T.R., Laklai, H., Sugimoto, H., Kahlert, C., Novitskiy, S.V., et al. (2014). Depletion of carcinoma-associated fibroblasts and fibrosis induces immunosuppression and accelerates pancreas cancer with reduced survival. Cancer Cell *25*, 719–734.

Peng, J., Sun, B.F., Chen, C.Y., Zhou, J.Y., Chen, Y.S., Chen, H., Liu, L., Huang, D., Jiang, J., Cui, G.S., et al. (2019). Single-cell RNA-seq highlights intra-tumoral heterogeneity and malignant progression in pancreatic ductal adenocarcinoma. Cell Res. *29*, 725–738.

Porter, R.L., Magnus, N.K.C., Thapar, V., Morris, R., Szabolcs, A., Neyaz, A., Kulkarni, A.S., Tai, E., Chougule, A., Hillis, A., et al. (2019). Epithelial to mesenchymal plasticity and differential response to therapies in pancreatic ductal adenocarcinoma. Proc. Natl. Acad. Sci. USA *116*, 26835–26845.

Reichert, M., Bakir, B., Moreira, L., Pitarresi, J.R., Feldmann, K., Simon, L., Suzuki, K., Maddipati, R., Rhim, A.D., Schlitter, A.M., et al. (2018). Regulation of epithelial plasticity determines metastatic organotropism in pancreatic cancer. Dev. Cell *45*, 696–711.e8.

Rhim, A.D., Mirek, E.T., Aiello, N.M., Maitra, A., Bailey, J.M., McAllister, F., Reichert, M., Beatty, G.L., Rustgi, A.K., Vonderheide, R.H., et al. (2012). EMT and dissemination precede pancreatic tumor formation. Cell *148*, 349–361.

Shah, A.N., Summy, J.M., Zhang, J., Park, S.I., Parikh, N.U., and Gallick, G.E. (2007). Development and characterization of gemcitabine-resistant pancreatic tumor cells. Ann. Surg. Oncol. *14*, 3629–3637.

Siegel, R.L., Miller, K.D., and Jemal, A. (2019). Cancer statistics, 2019. CA Cancer J. Clin. *69*, 7–34.

Stuart, T., Butler, A., Hoffman, P., Hafemeister, C., Papalexi, E., Mauck, W.M., 3rd, Hao, Y., Stoeckius, M., Smibert, P., and Satija, R. (2019). Comprehensive integration of single-cell data. Cell *177*, 1888–1902.e21.

Subramanian, A., Tamayo, P., Mootha, V.K., Mukherjee, S., Ebert, B.L., Gillette, M.A., Paulovich, A., Pomeroy, S.L., Golub, T.R., Lander, E.S., and Mesirov, J.P. (2005). Gene set enrichment analysis: a knowledge-based approach for interpreting genome-wide expression profiles. Proc. Natl. Acad. Sci. USA *102*, 15545–15550.

Szklarczyk, D., Gable, A.L., Lyon,D., Junge, A., Wyder, S., Huerta-Cepas, J., Simonovic, M., Doncheva, N.T., Morris, J.H., Bork, P., et al. 2019. STRING v11: protein–protein association networks with increased coverage, supporting functional discovery in genome-wide experimental datasets. *Nucleic acids research* 47.D1, D607-D613.2.

Tan, T.Z., Miow, Q.H., Miki, Y., Noda, T., Mori, S., Huang, R.Y., and Thiery, J.P. (2014). Epithelial-mesenchymal transition spectrum quantification and its efficacy in deciphering survival and drug responses of cancer patients. EMBO Mol. Med. *6*, 1279–1293.

Timpson, P., McGhee, E.J., Morton, J.P., von Kriegsheim, A., Schwarz, J.P., Karim, S.A., Doyle, B., Quinn, J.A., Carragher, N.O., Edward, M., et al. (2011). Spatial regulation of RhoA activity during pancreatic cancer cell invasion driven by mutant p53. Cancer Res. *71*, 747–757.

van Dijk, D., Sharma, R., Nainys, J., Yim, K., Kathail, P., Carr, A.J., Burdziak, C., Moon, K.R., Chaffer, C.L., Pattabiraman, D., et al. (2018). Recovering gene interactions from single-cell data using data diffusion. Cell *174*, 716–729.e27.

Von Burstin, J., Eser, S., Paul, M.C., Seidler, B., Brandl, M., Messer, M., Von Werder, A., Schmidt, A., Mages, J., Pagel, P., et al. (2009). E-cadherin regulates metastasis of pancreatic cancer in vivo and is suppressed by a snail/hdac1/hdac2 repressor complex. Gastroenterology *137*, 361–371, 371.e1–371.e5.

Wang, M., Estrella, J.S., Katz, M.H., Kim, M., Rashid, A., Lee, J.E., Maitra, A., Wistuba, I.I., Wolff, R.A., Varadhachary, G.R., and Wang, H. (2019). Expression of epithelial-mesenchymal transition markers in treated pancreatic ductal adenocarcinoma. Pancreas *48*, 1367–1372.

Wellner, U., Schubert, J., Burk, U.C., Schmalhofer, O., Zhu, F., Sonntag, A., Waldvogel, B., Vannier, C., Darling, D., zur Hausen, A., et al. (2009). The EMT-activator ZEB1 promotes tumorigenicity by repressing stemness-inhibiting microRNAs. Nat. Cell Biol. *11*, 1487–1495.

Williams, E.D., Gao, D., Redfern, A., and Thompson, E.W. (2019). Controversies around epithelial-mesenchymal plasticity in cancer metastasis. Nat. Rev. Cancer *19*, 716–732.

Yang, J., Antin, P., Berx, G., Blanpain, C., Brabletz, T., Bronner, M., Campbell, K., Cano, A., Casanova, J., Christofori, G., et al.; EMT International Association (TEMTIA) (2020). Guidelines and definitions for research on epithelial-mesenchymal transition. Nat. Rev. Mol. Cell Biol. *21*, 341–352.

Zheng, X., Carstens, J.L., Kim, J., Scheible, M., Kaye, J., Sugimoto, H., Wu, C.C., LeBleu, V.S., and Kalluri, R. (2015). Epithelial-to-mesenchymal transition is dispensable for metastasis but induces chemoresistance in pancreatic cancer. Nature *527*, 525–530.

CellPress
OPEN ACCESS

STAR★METHODS

KEY RESOURCES TABLE

REAGENT or RESOURCE	SOURCE	IDENTIFIER
**Antibodies**		
Rabbit monoclonal anti-Slug	Cell Signaling Technology	Cat# 9585; RRID:AB_2239535
Rabbit polyclonal anti-FSP1	Agilent (originally DAKO)	Cat# A5114; RRID:AB_2335679
Rabbit polyclonal anti-Zeb1	Novus	Cat# NBP1-05987; RRID:AB_2273178
Rat monoclonal anti-cytokeratin 8	DSHB	Cat# TROMA-I; RRID:AB_531826
Chicken polyclonal anti-GFP	Aves	Cat# GFP-1020; RRID:AB_10000240
Mouse monoclonal anti-Twist1	Novus	Cat# NBP2-37364; RRID:AB_2801339
Rabbit monoclonal anti-Vimentin	Cell Signaling Technology	Cat# 5741; RRID:AB_10695459
Rabbit polyclonal anti-Snail + Slug	Abcam	Cat# ab180714; RRID:AB_2728773
Mouse monoclonal anti-aSMA	Agilent (originally DAKO)	Cat# M0851; RRID:AB_2223500
Rabbit monoclonal anti-Ecadherin	Cell Signaling Technology	Cat# 3195; RRID:AB_2291471
Rabbit monoclonal anti-Ki-67	Thermo Scientific	Cat# RM-9106-S; RRID:AB_149707
Rabbit on Rodent HRP Polymer	Biocare Medical	Cat# RMR622
Mouse-on-Mouse HRP-Polymer	Biocare Medical	Cat# MM620
Polink-1 HRP Rat-NM for DAB Bulk Kit	GBI labs	Cat# D35-110
Polink-2 Plus HRP Chicken IgY for DAB Kit	GBI labs	Cat# D84-60
Goat polyclonal anti-Rabbit Alexa Fluor 488	Invitrogen	Cat# A-11008; RRID: AB_143165
Goat polyclonal anti-Chicken Alexa Fluor 594	Invitrogen	Cat# A-11042; RRID: AB_2534099
Goat polyclonal anti-Rat Alexa Fluor 647	Invitrogen	Cat# A-21247; RRID: AB_141778
Goat polyclonal anti-Rabbit, Biotinylated	Vector Laboratories	Cat# BA-1000; RRID:AB_2313606
**Bacterial and virus strains**		
Ad-CMV-iCre	Vector Biolabs	Cat# 1045
**Biological samples**		
mouse model pancreatic tumor and mixed organ FFPE blocks	This paper	N/A
**Critical commercial assays**		
Opal 7 color IHC Kit	Akoya Biosciences	NEL811001KT
TSA Coumarin System	Akoya Biosciences	NEL703001KT
VECTASTAIN Elite® ABC System	Vector Labs	PK-6100
Power SYBR Green PCR Master Mix	Thermo Fisher	4367659
Fluoroshield mounting media	Sigma Aldrich	F618
Vectashield Mounting Media	Vector Laboratories	H1000
**Deposited data**		
Raw and analyzed microarray data	This paper	GEO: GSE164612
Raw and analyzed scRNA-seq data	This paper	GEO: GSE165534
GDC TCGA Pancreatic Cancer (PAAD)	TCGA	https://xenabrowser.net/datapages/
Patient scRNA-seq data	Peng et al., 2019	N/A
Source Data	N/A	Mendeley Data: https://dx.doi.org/10.17632/sxc5zk4m9w.1
**Experimental models: Cell lines**		
Control Line 978U; Pdx1-Cre; Kras^{G12D}; p53$^{R172H/+;}$ Rosa26-LSL-EYFP$^{F/F}$; Female	This paper	N/A
Control Line H444; Pdx1-Cre; Kras^{G12D}; p53$^{R172H/+}$; Rosa26-LSL-EYFP$^{F/+}$;Female	This paper	N/A

(Continued on next page)

Continued

REAGENT or RESOURCE	SOURCE	IDENTIFIER
Control Line O739; Pdx1-Cre; $Kras^{G12D}$; $p53^{R172H/+}$; $Rosa26\text{-}LSL\text{-}EYFP^{F/+}$;Male	This paper	N/A
KPC-ST Line 309P; Pdx1-Cre; $Kras^{G12D}$; $p53^{R172H/+}$; $Snai1^{F/F}$; $Twist1^{F/F}$; $Rosa26\text{-}LSL\text{-}EYFP^{F/+}$; αSMA-RFP; Female	This paper	N/A
KPC-ST Line 404P; Pdx1-Cre; $Kras^{G12D}$; $p53^{R172H/+}$; $Snai1^{F/F}$; $Twist1^{F/F}$; $Rosa26\text{-}LSL\text{-}EYFP^{F/+}$; αSMA-RFP; Female	This paper	N/A
KPC-ST Line 407P; Pdx1-Cre; $Kras^{G12D}$; $p53^{R172H/+}$; $Snai1^{F/F}$; $Twist1^{F/F}$; $Rosa26\text{-}LSL\text{-}EYFP^{F/F}$; αSMA-RFP; Female	This paper	N/A
KPC Ecad KO Line 28B; Adenoviral-Cre; $Kras^{G12D}$; $p53^{R172H/+}$; $Cdh1^{F/F}$; $Rosa26\text{-}LSL\text{-}EYFP^{F/F}$; Female	This paper	N/A
Experimental models: Organisms/strains		
KPC; Pdx1-Cre; $Kras^{G12D}$; $p53^{R172H/+}$ (may contain Rosa26-LSL-EYFP or αSMA-RFP)	This paper	N/A
KPC;ST; Pdx1-Cre; $Kras^{G12D}$; $p53^{R172H/+}$; $Snai1^{F/F}$; $Twist1^{F/F}$ (may contain Rosa26-LSL-EYFP or αSMA-RFP)	This paper	N/A
KPPC; P48-Cre; $Kras^{G12D}$; $p53^{F/F}$ (may contain Rosa26-LSL-EYFP)	This paper; Dr. Rhim	N/A
KPPC;Z^{F+}; P48-Cre; $Kras^{G12D}$; $p53^{F/F}$; $Zeb1^{F/+}$ (may contain Rosa26-LSL-EYFP)	This paper; Dr. Rhim	N/A
KPPC;Z^{cKO} $^{+}$; P48-Cre; $Kras^{G12D}$; $p53^{F/F}$; $Zeb1^{F/F}$ (may contain Rosa26-LSL-EYFP)	This paper; Dr. Rhim	N/A
KP;E^{FF}-AdCre $^{+}$; Adenoviral-Cre; $Kras^{G12D}$; $p53^{R172H/+}$; $Cdh1^{F/F}$ (may contain Rosa26-LSL-EYFP or αSMA-RFP)	This paper	N/A
Oligonucleotides		
mouse Snai1 Forward; CACACGCTGCCTTGTGTCT	Lovisa et al., 2015	N/A
mouse Snai1 Reverse; GGTCAGCAAAAGCACGGTT	Lovisa et al., 2015	N/A
mouse Twist1 Forward; CTGCCCTCGGACAAGCTGAG	Lovisa et al., 2015	N/A
mouse Twist Reverse; CTAGTGGGACGCGGACATGG	Lovisa et al., 2015	N/A
mouse Zeb1 Forward; GCTGGCAAGACAACGTGAAAG	Harvard Primer Bank	N/A
mouse Zeb1 Reverse; GCCTCAGGATAAATGACGGC	Harvard Primer Bank	N/A
mouse Cdh1 Forward; GTCAACACCTACAACGCTGCC	Lovisa et al., 2015	N/A
mouse Cdh1 Reverse; GTTGTGCTCAAGCCTTCAC	Lovisa et al., 2015	N/A
mouse Vim Forward; CTTGAACGGAAAGTGGA ATCCT	Lovisa et al., 2015	N/A
mouse Vim Reverse; GTCAGGCTTGGAAAC GTCC	Lovisa et al., 2015	N/A
mouse Col1a1 Forward; CTCCTCTTAGGGGCCACT	Lovisa et al., 2015	N/A
mouse Col1a1 Reverse; CCACGTCTCACCATTGGGG	Lovisa et al., 2015	N/A
mouse 18s Forward; GTAACCCGTTGAACCCCATT	Lovisa et al., 2015	N/A
mouse 18s Reverse; CCATCCAATCGGTAGTAGCG	Lovisa et al., 2015	N/A
mouse Rhoa Forward; AGCTTGTGGTAAGACAT GCTTG	Harvard Primer Bank	N/A
mouse Rhoa Reverse; GTGTCCCATAAAGCCAACT CTAC	Harvard Primer Bank	N/A
mouse Rock1 Forward; GACTGGGGACAGTTTTGAGAC	Harvard Primer Bank	N/A
mouse Rock1Reverse; GGGCATCCAATCCATCCAGC	Harvard Primer Bank	N/A
mouse Rac1 Forward; GAGACGGAGCTGTTGGTAAAA	Harvard Primer Bank	N/A
mouse Rac1Reverse; ATAGGCCCAGATTCACTGGTT	Harvard Primer Bank	N/A
mouse Sdc1 Forward; GAGACGGAGCTGTTGGTAAAA	Harvard Primer Bank	N/A
mouse Sdc1 Reverse; AACGGGCCTCAACAGTCAG	Harvard Primer Bank	N/A
mouse Ctsb Forward; TCCTTGATCCTTCTTTCTTGCC	Harvard Primer Bank	N/A

(*Continued on next page*)

Continued

REAGENT or RESOURCE	SOURCE	IDENTIFIER
mouse Ctsb Reverse; ACAGTGCCACACAGCTTCTTC	Harvard Primer Bank	N/A
mouse Lamp1 Forward; CAGCACTCTTTGAGGTGAAAAAC	Harvard Primer Bank	N/A
mouse Lamp1 Reverse; ACGATCTGAGAACCATTCGCA	Harvard Primer Bank	N/A
mouse Max Forward; ACCATAATGCACTGGAACGAAA	Harvard Primer Bank	N/A
mouse Max Reverse; GTCCCGCAAACTGTGAAAGC	Harvard Primer Bank	N/A
mouse Jun Forward; CCTTCTACGACGATGCCCTC	Harvard Primer Bank	N/A
mouse Jun Reverse; GGTTCAAGGTCATGCTCTGTTT	Harvard Primer Bank	N/A
mouse Sec24b Forward; GGTCCAGCACAGAGTCCAATG	Harvard Primer Bank	N/A
mouse Sec24b Reverse; GGAGTCCCCGAATTTTGTGTT	Harvard Primer Bank	N/A
mouse Ndufb11 Forward; CCTCCAGGGCTGTAATCGC	Harvard Primer Bank	N/A
mouse Ndufb11 Reverse; GGTTCTTCGCGTAGACGTTTTC	Harvard Primer Bank	N/A
mouse Apoe Forward; CTGACAGGATGCCTAGCCG	Harvard Primer Bank	N/A
mouse Apoe Reverse; CGCAGGTAATCCCAGAAGC	Harvard Primer Bank	N/A
Software and algorithms		
Prism 8	GraphPad	N/A
InForm v2.4.6	Akoya Biosciences	N/A
Vis 6.2019	Visiopharm	N/A
UCSC Xena browser	Goldman et al., 2020	N/A
Seurat (version 3.6.1)	Butler et al., 2018; Stuart et al., 2019	N/A
library Matrix and library Rmagic	van Dijk et al., 2018	N/A
Monocle 3	Haghverdi et al., 2018	N/A
L-Function	Carstens et al., 2017	N/A
FIJI with Phantast plugin	NIH; Jaccard et al., 2014	N/A
QuPath	Bankhead et al., 2017	N/A
SPSS Statistics	IBM	N/A

RESOURCE AVAILABILITY

Lead contact

Further information and requests for resources and reagents should be directed to and will be fulfilled by the Lead Contact, Raghu Kalluri, MD, PhD (rkalluri@mdanderson.org).

Materials availability

Zeb1-floxed allele reported in this study was generated from the Knockout Mouse Project (KOMP) plasmid (tm1a(KOMP)Wtsi).

There are restrictions to the availability of the mouse lines and generated cell lines from these mouse models due to institutional property rights and require an MTA for transfer. Please contact the Lead author for more information.

Data and code availability

The accession number for the murine single-cell RNA-sequencing data reported in this paper is GEO: GSE165534. The accession number for the murine YFP sorted cancer cell microarray is GEO: GSE164612.

Original data have been deposited into Mendeley Data: https://dx.doi.org/10.17632/sxc5zk4m9w.1.

EXPERIMENTAL MODEL AND SUBJECT DETAILS

Mouse studies

Characterization of disease progression and genotyping for the *Pdx1-Cre; LSL-Kras*^{G12D}*; P53*$^{R172H/+}$ *; R26-LSL-EYFP* (herein referred to as KPC), *Pdx1-Cre; LSL-Kras*^{G12D}*; P53*$^{R172H/+}$; *Snai1*$^{L/L}$*; R26-LSL-EYFP* (herein referred to as KPC; Snail^{cKO}), *Pdx1-Cre; LSL-Kras*^{G12D}*; P53*$^{R172H/+}$; *Twist1*$^{L/L}$*; R26-LSL-EYFP* (herein referred to as KPC; Twist^{cKO}) mice were previously described (Zheng et al., 2015). The KPC; Snai1^{cKO} and KPC; Twist^{cKO} were bred to generate the KPC; Snail^{cKO}; Twist^{cKO} dual conditional knockout line (herein referred to as KPC;ST). The KPC;ST mice were maintained on a mixed background, bred according to

Mendelian ratios, and developed normally (data not shown). KPC;Snail^{F+};Twist^{F+} mice showed no differences in all assays investigated from the KPC;Snail^{++};Twist^{++} mice and were included in the KPC control group. *p48-Cre; LSL-Kras^{G12D}; P53$^{L/L}$* (herein referred to as KPPC) mice were previously described (Rhim et al., 2012; Guerra and Barbacid, 2013) . The *Zeb1$^{L/+}$* allele was generously provided by Dr. Andrew Rhim and is based on the KOMP tm1a construct post Flpase recombination. Mice were maintained on a mixed background, bred according to Mendelian ratios, and developed normally (data not shown).

All mice were housed under standard housing conditions at MD Anderson Cancer Center (MDACC) animal facilities, and all animal procedures were reviewed and approved by the MDACC Institutional Animal Care and Use Committee. Investigators were not blinded for group allocation, but were blinded for the assessment of the phenotypic outcome assessed by histological analyses. Males and females were utilized equally. No randomization method was used and all mice of the desired genotype were enrolled in the studies. Age, disease progression and gender are reported for each mouse in Table S2.

Primary tissue sorting and cell line generation

Primary cancer cell lines were generated similar to a previous publication (Zheng et al., 2015). In brief, tumor tissue was minced with scissors in a 1.5 mL tube, and incubated at 37°C in 4 mg/mL Collagenase IV (GIBCO, 17104019) and 4 mg/mL Dispase II (GIBCO, 17105041) in serum-free RPMI in a 6-well dish for 1 hour. Cells were resuspended in 10 mL of complete media (RPMI with 20% FBS and 1% PSA) and strained through a 70 um strainer and pelleted to removed digestion media. For cell line generation, the cells were then resuspended in 2 mL of complete media and plated on 6-well Collagen IV coated plates (Corning BioCoat, 62405-636), after reaching approximately 80% confluency the cells were then passaged onto standard T25 tissue culturing flasks. For single cell sorting, the pelleted cells were washed twice in PBS, resuspended in FACS buffer (PBS with 2% FBS) and sorted for YFP positivity by flow cytometry.

The stabilized mesenchymal KPC; E-cadherin-KO control line was isolated from the primary tumor generated by the adenoviral-Cre mediated transformation of the pancreas in a KP;E-cadherin^{FF} mouse. In brief, the previously published *Cdh1$^{L/+}$* allele (Derksen et al., 2006) was crossed into the KPC background. Adenoviral vector injections were roughly based upon a previous publication (Gierut et al., 2014). Cre negative KP;E-cadherin^{FF} mice were injected in the tail of the pancreas with 5x10^{6} pfu in 25 μL (5 μL of 1x10^{9} pfu virus + 23 μL of MEM + 2 M $CaCl_2$) of adenoviral Cre (Vector BioLabs 1045) per mouse. Tumor tissue was collected at necropsy and a cell line was isolated as described, above.

All cell lines used in the study were validated for genotype, recombination purity (to exclude stromal cell contamination) and EMT expression status through qPCR. Details for genotype and sex of each cell line are provided in the Key resources table.

METHOD DETAILS

Single-cell RNA-sequencing

Patient scRNA-seq data were mined from Peng et al. (2019). Whole pancreatic tumor tissue from six KPC (3 early and 3 late) and six (1 early and 5 late) KPC;ST mice and liver tissue from 3 KPC late and 4 KPC;ST late mice were processed to obtain a single-cell suspension for each tissue. Tumor tissue was minced and digested with Collagenase IV (4 mg/mL) and Dispase (4 mg/mL) in serum-free DMEM, shaking at 37°C for 30 minutes. The suspension was then passed through a 70 um and 40 um filter and washed twice with DMEM containing 20% FBS. Cells were stained for 30 minutes on ice in 1:100 of eFluor 780 fixable viability dye (eBioscience 65-0865-14). Cells were sorted on a BD FACS Aria for all live or live/endogenous YFP^{+} as appropriate. scRNA-seq on these samples was conducted at the MDACC Advanced Technology Genomics Core. Single-cell Gel Bead-In-Emulsions (GEMs) generation and barcoding, post GEM-RT cleanup and cDNA amplification, library construction and Illumina-ready sequencing library generation were prepared following the manufacturer's guidelines. High Sensitivity dsDNA Qubit kit was used to estimate the cDNA and library concentration. HS DNA Bioanalyzer was used for the quantification of cDNA. DNA 1000 Bioanalyzer was used for the quantification of libraries. The "c-loupe" files were generated by using Cell Ranger software pipelines following the manufacturer's guidelines. Cells from the unfractionated tumor were encapsulated using 10X Genomics' Chromium controller and Single-Cell 3′ Reagent Kits v2. Following capture and lysis, cDNA was synthesized and amplified to construct Illumina sequencing libraries. The libraries from approximately 1,000- 4,000 cells per sample were sequenced with Illumina Nextseq 500 or HiSeq 3000. The run format was 26 cycles for read 1, 8 cycles index 1, and 124 cycles for read 2. scRNA-seq data were processed by the Advanced Technology Genomics Core at MDACC.

Library Seurat (version 3.6.1)(Butler et al., 2018; Stuart et al., 2019), dplyr and cowplot were loaded into R (version 3.6.1) to explore QC metrics, filter cells, normalize data, cluster cells, and identify cluster biomarkers. To filter out low-quality cells, a threshold with a minimum of 200 and a maximum of 4000-7000 genes per cell was used. Cells with more than 10% of the mitochondrial genome were also removed from further analysis. To remove the influence of technical characteristics from downstream analyses, "sctransform" package was used for normalization. "RunUMAP" function was used for clustering the cells. "FindAllMarkers" function was used to identify the specific markers for each cell cluster as well as downstream analysis on gene expression. "DoHeatmap" function was used to show the top genes in each cluster. "VlnPlot" function was used to show expression probability distributions across cell clusters of the genes selected to assign the cell type identity and the genes of interest.

The MAGIC algorithm utilizes nearest neighbor graphing and a diffusion operator to restore or "smooth" missing transcripts from the single-cell expression data based on the expression of similar cells (van Dijk et al., 2018). MAGIC smoothing of the cancer cell cluster was run using the library Matrix and library Rmagic (van Dijk et al., 2018) based off of a 315 EMT gene signature (Tan

et al., 2014). The exact resolution/cluster numbers were selected based on the consistency of the expression heatmaps within each cluster. To validate our results obtained by MAGIC processing using the 315 Thiery gene signature (305 genes for human and 294 genes for the mouse were discoverable in our dataset), we utilized a second MAGIC based method utilizing principle component analysis (PCA): magic(data, genes = "pca_only"). 3D scatterplots of the results were plotted and color-coded for CDH1, VIM, FN1, and ZEB1.

The post-MAGIC Thiery EMT Signature expression values were used to define cancer cell EMT stage. An "E/M score" was calculated by taking the Log2 of the percentage of positive epithelial genes (from the scaled.data) divided by the percentage of positive mesenchymal genes: Log2(EPI/MES). Log2 values to set general boundaries for clinical correlate analyses and comparison of mouse signatures to the human were determined as follows: Epithelial ≥ 1.5, Partial-EMT < 1.5 and > -1.5, and Mesenchymal ≤ -1.5.

Cancer cells, defined by Seurat, were further analyzed by Monocle 3 (Haghverdi et al., 2018) to construct single-cell trajectories and order the cells in pseudotime. The figures related to pseudotime were generated by "learn_graph," "plot_cells," and "order_cells." The area enriched for both KPC early and KPC;ST early cells was defined as the root nodes for pseudotime. Modules of co-regulated genes were found by "find_gene_modules."

Histology and histopathology

Histology and histopathological scoring were previously described (Zheng et al., 2015). Formalin-fixed tissues were embedded in paraffin and sectioned at 5 μm thickness. Sections were stained with hematoxylin and eosin (H&E). Histopathological measurements were made by scoring H&E stained tumors for relative percentages of each histopathological phenotype: normal (non-neoplastic), PanIN, well-differentiated PDAC, moderately-differentiated PDAC, poorly-differentiated PDAC, or necrosis. The weighted pathology scores were calculated by taking the relative percentages for each phenotype and assigning a weighted score (Normal > 5% = 1; PanIN > 30% = 2; PDAC > 30% = 4; Poorly-differentiation PDAC > 5% = 5; and Necrosis > 5% = 6). These scores were then summed to produce the weighted pathology scores presented in Figure 4. When tumor histology was missing or of poor quality, the mice were excluded from all histological analyses and this was determined blinded from genotype information. Microscopic metastases were observed in H&E stained tissue sections of at least four and three depths, 150 μm apart for the liver and lung, respectively. These data have been presented as a contingency table (Table S3) and represented as the number of positive tissues (one or more lesions in a tissue) out of the number of tissues scored. The "Any" metastasis score is the number of mice positive for a secondary lesion found anywhere throughout the body out of the total number of mice scored. Metastatic lesion numbers and areas were processed by circling every metastatic lesion in each tissue section using scanned slides and the Aperio ImageScope software. Data represented as indicated numbers per tissue depth.

Immunohistochemical staining and analyses

Antigen retrieval conditions, antibody concentration and company details are outlined in Table S4. In brief, tissues were fixed in 10% formalin overnight, dehydrated, embedded in paraffin and 5-μm-thick sections were deparaffinized for subsequent staining. Immunofluorescence was performed by antigen retrieval in heated citric acid buffer (pH 6.0) or Tris-EDTA with 0.1% Tween 20 (pH 9.0) by microwave (EZ Retriever microwave, BioGenex) or pressure cooker as indicated. Unless otherwise indicated, each combination of staining was put through sequential rounds of staining, each including a protein block, followed by a primary antibody and a corresponding secondary antibody directly conjugated to the indicated fluorophore or HRP-conjugated polymer followed by covalent binding of an Opal fluorophore using tyramide signal amplification (Perkin Elmer Opal7 Kit). Sections were either stained with DAPI (1:20,000) and mounted with VectaShield mounting media or mounted with a DAPI containing mounting media (Sigma). For immunohistochemical staining, sections underwent antigen retrieval, incubation in hydrogen peroxide (3%), protein block, primary and secondary antibody incubations with biotinylated conjugated antibody, amplified with streptavidin-HRP conjugation (ABC kit, VectorLabs) and developed by DAB according to the manufacturer's recommendations.

Representative images were taken using a Leica DM1000 light microscope, a Vectra3 Multispectral Imaging System (Akoya Biosciences), or a Zeiss AxioScan.Z1 at 20x magnification and analyzed using cell segmentation and scoring on inForm (v2.4.6; Akoya) or QuPath (Bankhead et al., 2017) (for Ki-67). For EMT quantification, YFP^+ cancer cells were identified by setting a threshold with the inForm scoring algorithms and then hand counted as being EMT^+ (co-expression of any mesenchymal marker and YFP) and presented as the average percentage double positive out of YFP positive cells per image per mouse. Immune population data on a patient TMA was mined from previously published data (Carstens et al., 2017). Spectrally unmixed images were scored for $CK8^+aSMA^+$ cells using inForm phenotyping. Mouse immune population data were unmixed using inForm and analyzed for tissue segmentation, cell segmentation and phenotyping (aka phenomapping) using Vis analysis software from Visiopharm. XY coordinates for each cell extracted from multiplexed images were used to compute the extent of T cell infiltration around cancer cells using the L-function as described (Carstens et al., 2017).

CTC assays

Blood (1,000 μL) was collected at necropsy and incubated with 13 mL of ACK lysis buffer (A1049201, GIBCO) for five minutes at room temperature to lyse red blood cells. Cell pellets were washed twice with PBS and resuspended in 300 μL of PBS containing 2% FBS and analyzed for the number of YFP^+ cells by flow cytometry (BD FACSAria IIIu Cell Sorter).

Quantitative reverse-transcriptase PCR analyses (qPCR)

RNA was extracted from cultured primary PDAC cells using TRIzol (15596026, Life Technologies). cDNA synthesis was performed using the Applied Biosystems cDNA synthesis kit according to the manufacturer's directions. Quantitative PCR was performed to analyze the gene expression profiles of the listed genes using SYBR Green PCR Master Mix in a 7300 Sequence Detector System (Applied Biosystems), and measurements were standardized to the expression of the 18s housekeeping gene. The expression data are presented as fold change (2ˆddCt) with the averaged control group normalized to a fold value of 1. dCt were used to measure statistical significance in observed changes. Genes and primer sequences are in Key resources table.

Microarray analyses

YFP^+ cells were single-cell sorted at the South Campus Flow Cytometry core (BD FACSAria IIIu Cell Sorter). RNA was isolated using either Trizol or PicoPure RNA isolation kit (Applied Biosystems). Total RNA was quantified using an RNA6000 Pico or Nano assays (Aligent), whole transcriptome analysis was performed using the WT Plus Assay (Applied Biosystems) and run using the Mouse Clariom D array (Applied Biosystems) by the MDACC Sequencing and ncRNA Core Program. Gene expression analysis was performed using the TAC4.0 version 2 software. Gene set enrichment analysis (GSEA) was performed for KEGG, biocarta, c5bp, and hallmarks gene sets using GSEA software version 4.0.0 and MSigDB 7.0 (Broad Institute). Gene expression microarray data have been deposited in GEO: GSE164612. Ingenuity pathway analysis (QIAGEN) was run on the expression values of the primary cancer cells using 2.1 fold and $p < 0.05$ as cutoffs.

Migration assays

Primary PDAC cell lines that had been confirmed by qPCR for expression of EMT genes, were plated densely in 3 wells of a 24-well plate for each line. When the cells reached confluency, the media was removed and a vertical scratch was made in the center of each well with a P100 pipette tip. Wells were washed twice in PBS and the media was replaced (RPMI, 20% FBS, 1% PSA). Each well was imaged using a 4x phase objective every 30 minutes for 40 hours using the Olympus BX51 Live Imaging Microscope and Scope software. The experiment was repeated three times for lines 28B and 407B and four times for all others. Exported tif images were processed using FIJI (ImageJ, NIH) and the PHANTAST cell segmentation plugin (Jaccard et al., 2014). The sigma value was kept constant at 1.2 and the epsilon value was adjusted between 0.05 and 0.15 to compensate for lighting changes of each well, but kept constant over the time course for each well. Images were then inverted and particle count and area (confluency) were analyzed.

QUANTIFICATION AND STATISTICAL ANALYSIS

Statistical analyses were performed using unpaired two-tailed t tests, one-way and two-way ANOVA with Tukey's multiple comparisons test using GraphPad Prism version 8 and 9, as stipulated in the figure legends. χ^2 analyses, using SPSS statistical software, were performed comparing control to conditional knock-out groups across multiple histological parameters. Results are outlined in Table S3. χ^2 analyses were performed on scRNA-seq patients comparing each of the defined E/M groups across all provided clinical and histopathological parameters. Results are outlined in Table S1. Analysis of TCGA Pancreatic Cancer (PAAD) was performed using the UCSC Xena browser (Goldman et al., 2020). Kaplan-Meier plots were drawn for survival analysis and the log rank Mantel-Cox test was used to evaluate statistical differences, using GraphPad Prism 8. Data met the assumptions of each statistical test, where variance was not equal (determined by an F-test) Welch's correction for unequal variances was applied. Error bars represent s.e.m. Multiple visual fields were averaged to produce a single value for each animal which was then averaged again to represent the mean bar for the group in each graph. $p < 0.05$ was considered statistically significant.

Cell Reports, Volume *35*

Supplemental information

Stabilized epithelial phenotype of cancer cells in primary tumors leads to increased colonization of liver metastasis in pancreatic cancer

Julienne L. Carstens, Sujuan Yang, Pedro Correa de Sampaio, Xiaofeng Zheng, Souptik Barua, Kathleen M. McAndrews, Arvind Rao, Jared K. Burks, Andrew D. Rhim, and Raghu Kalluri

Figure S1

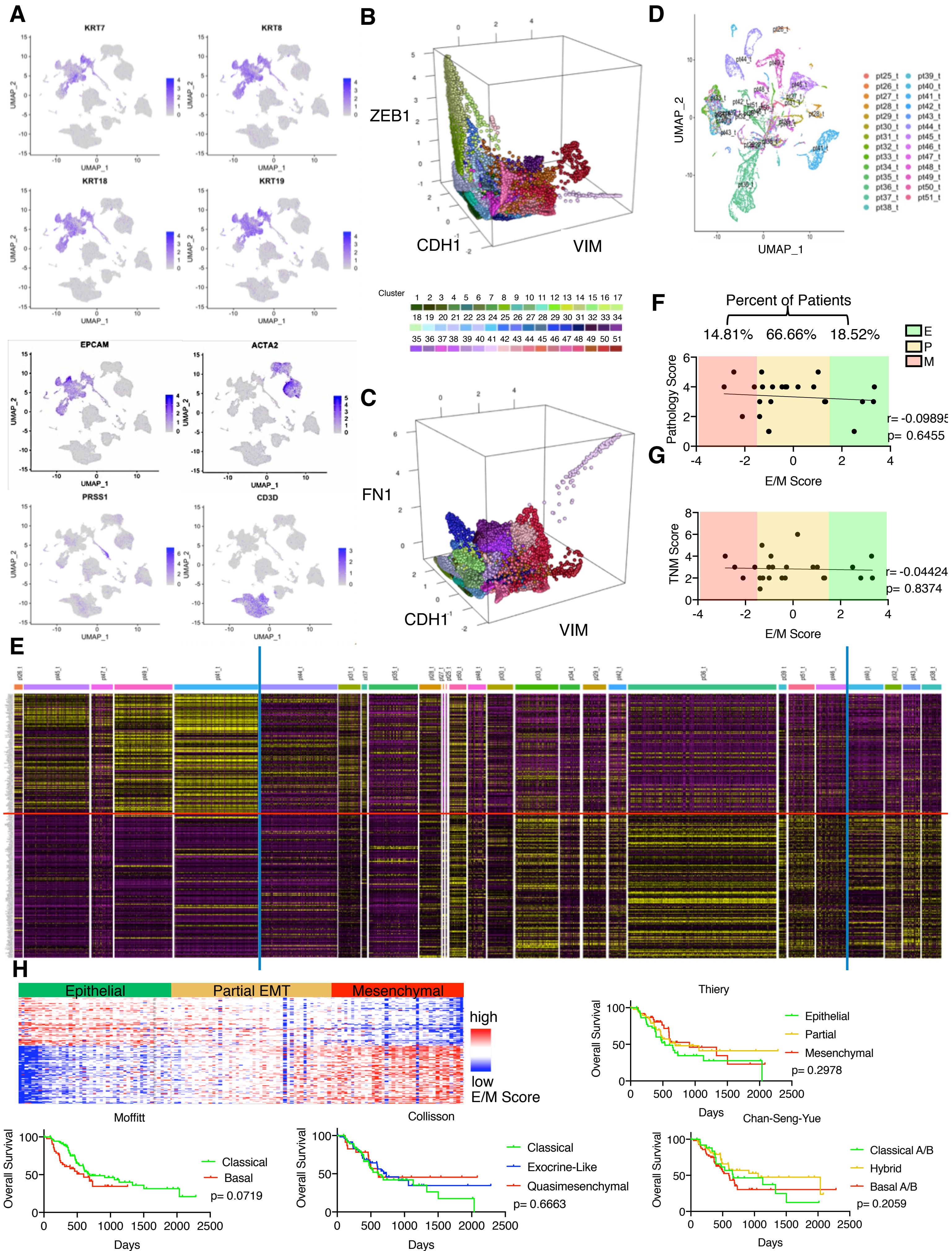
A
KRT7
KRT8
KRT18
KRT19
EPCAM
ACTA2
PRSS1
CD3D
UMAP_1
UMAP_2
B
ZEB1
CDH1
VIM
C
FN1
CDH1
VIM
Cluster
D
UMAP_1
UMAP_2
E
F
Percent of Patients
14.81%
66.66%
18.52%
E
P
M
Pathology Score
E/M Score
r= -0.09895
p= 0.6455
G
TNM Score
E/M Score
r= -0.04424
p= 0.8374
H
Epithelial
Partial EMT
Mesenchymal
high
low
E/M Score
Thiery
Overall Survival
Days
Epithelial
Partial
Mesenchymal
p= 0.2978
Moffitt
Classical
Basal
p= 0.0719
Collisson
Classical
Exocrine-Like
Quasimesenchymal
p= 0.6663
Chan-Seng-Yue
Classical A/B
Hybrid
Basal A/B
p= 0.2059

Figure S1. Single-cell RNA-sequencing of human PDAC reveals 51 different cancer cell phenotypes across the EMT continuum, Related to Figure 1.

A Seurat UMAP clustering of whole tissue single-cell populations with indicated gene expression heatmap overlays to demonstrate the selection of the cancer cell population as being high in *KRT7, KRT8, KRT18, KRT19*, and *EPCAM* but low in *ACTA2, PRSS1* and *CD3D*.

B-C Same clusters as **Figure 1B** plotted for the expression of (**b**) *ZEB1*, *CDH1* and *VIM* or (**c**) *FN1*, *CDH1* and *VIM*.

D UMAP clustering of patient cancer cells post MAGIC using the Thiery EMT signature color coded for each patient.

E Expression heatmap of the Thiery EMT signature for each patient. Epithelial genes are above the red line. Blue line indicates the cutoff to separate epithelial, partial, and mesenchymal phenotypes.

F-G Correlation plots comparing the tumor pathology score (**F**) and the TNM score (**G**) against the E/M score of each patient. The percentage of patients for each E/M grouped phenotype is listed above.

H Gene expression heatmap of the Thiery EMT signature in the TCGA pancreatic cancer cohort and survival plots for each indicated expression classifier.

Figure S2

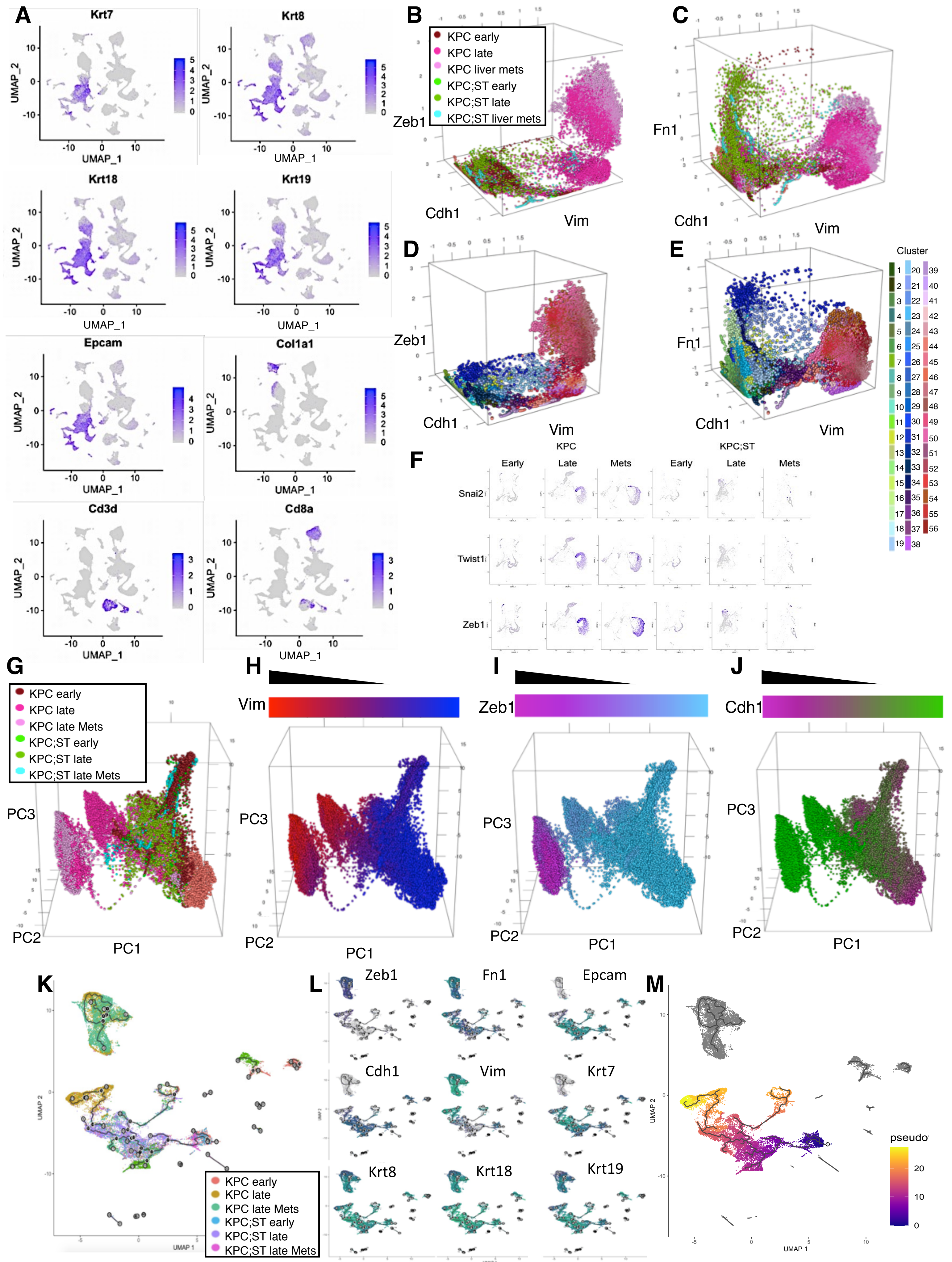
A
Krt7
Krt8
Krt18
Krt19
Epcam
Col1a1
Cd3d
Cd8a
UMAP_1
UMAP_2
B
KPC early
KPC late
KPC liver mets
KPC;ST early
KPC;ST late
KPC;ST liver mets
Zeb1
Cdh1
Vim
C
Fn1
Cdh1
Vim
D
Zeb1
Cdh1
Vim
E
Fn1
Cdh1
Vim
Cluster
F
KPC
KPC;ST
Early
Late
Mets
Snai2
Twist1
Zeb1
G
KPC early
KPC late
KPC late Mets
KPC;ST early
KPC;ST late
KPC;ST late Mets
PC3
PC2
PC1
H
Vim
I
Zeb1
J
Cdh1
K
KPC early
KPC late
KPC late Mets
KPC;ST early
KPC;ST late
KPC;ST late Mets
L
Zeb1
Fn1
Epcam
Cdh1
Vim
Krt7
Krt8
Krt18
Krt19
M
pseudot
UMAP 1
UMAP 2

Figure S2. Single-cell RNA-sequencing of murine PDAC identifies 56 cancer cell phenotypes across the EMT spectrum, Related to Figure 2.

A Seurat UMAP clustering of whole tissue single-cell populations with indicated gene expression heatmap overlays to demonstrate the selection of the cancer cell population as being high in *Krt7, Krt8, Krt18, Krt19*, and *Epcam*, but low in *Col1A1*, *Cd3d* and *Cd8a*.

B-E post-MAGIC clusters plotted for the expression of *Zeb1*, *Cdh1* and *Vim* (**B** and **D**) and *Fn1*, *Cdh1* and *Vim* (**C** and **E**) overlaid with (**B-C**) GEMM cohort or (**D-E**) E/M cluster identification.

F Magic cancer cell clusters separated by group and overlaid with expression heatmaps of EMT transcription factor genes.

G-J Cancer cell PCA analysis overlaid with cohort identifications or heatmaps for *Vim*, *Zeb1* or *Cdh1*, respectively.

K Trajectory analysis on Seurat cancer cell clusters color-coded by group.

L Expression overlays for indicated genes.

M pseudotime analysis.

Figure S3

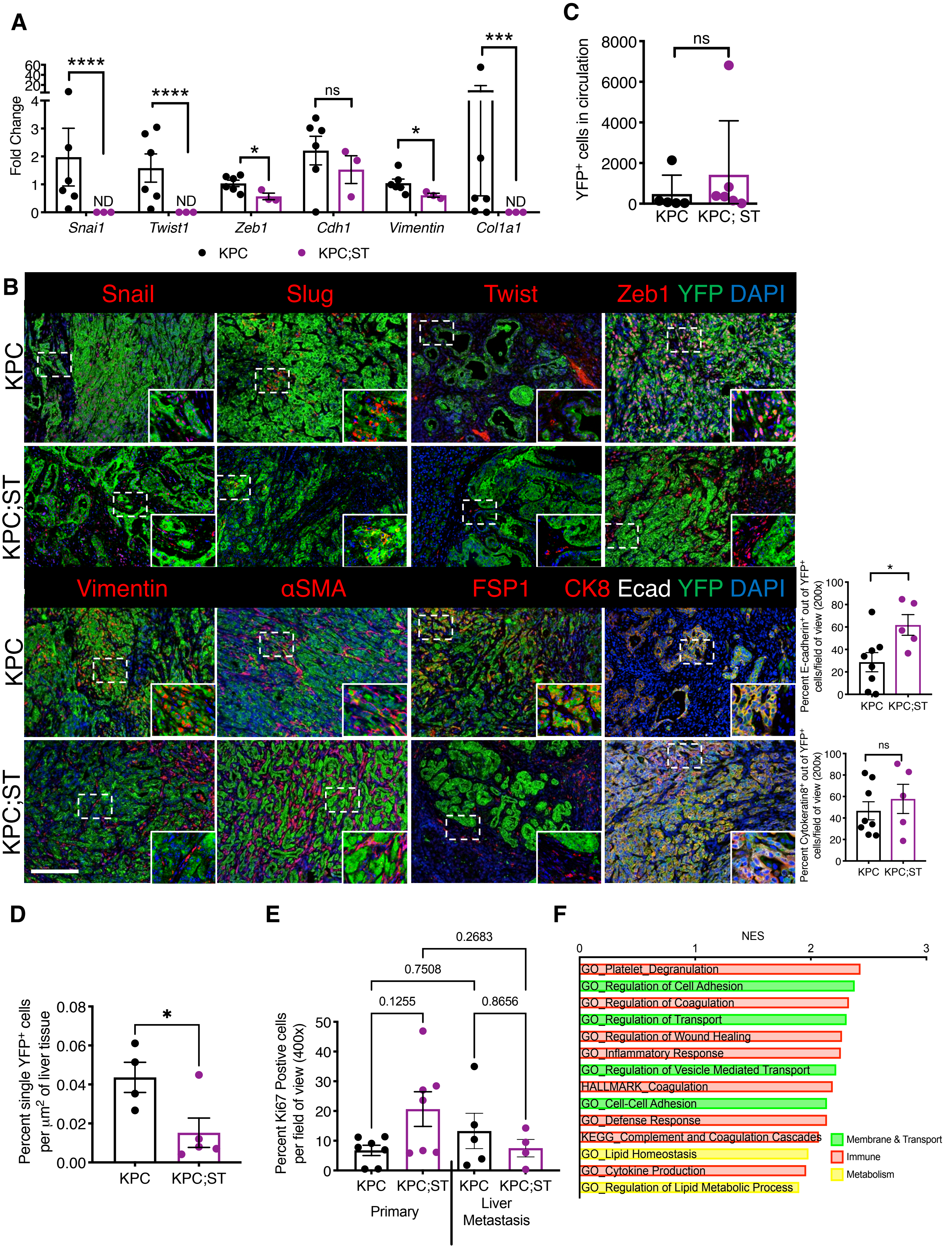
A
Fold Change
Snai1
Twist1
Zeb1
Cdh1
Vimentin
Col1a1
ND
****
***
*
ns
KPC
KPC;ST
C
YFP+ cells in circulation
KPC
KPC; ST
B
Snail
Slug
Twist
Zeb1 YFP DAPI
Vimentin
αSMA
FSP1
CK8 Ecad YFP DAPI
Percent E-cadherin+ out of YFP+ cells/field of view (200x)
Percent Cytokeratin8+ out of YFP+ cells/field of view (200x)
D
Percent single YFP+ cells per μm2 of liver tissue
E
Percent Ki67 Postive cells per field of view (400x)
0.2683
0.7508
0.1255
0.8656
Primary
Liver Metastasis
F
NES
GO_Platelet_Degranulation
GO_Regulation of Cell Adhesion
GO_Regulation of Coagulation
GO_Regulation of Transport
GO_Regulation of Wound Healing
GO_Inflammatory Response
GO_Regulation of Vesicle Mediated Transport
HALLMARK_Coagulation
GO_Cell-Cell Adhesion
GO_Defense Response
KEGG_Complement and Coagulation Cascades
GO_Lipid Homeostasis
GO_Cytokine Production
GO_Regulation of Lipid Metabolic Process
Membrane & Transport
Immune
Metabolism

Figure S3. Stabilized epithelial phenotype of PDAC cells enhances liver metastasis, Related to Figure 3.

A Fold change of qPCR gene expression analysis of *Snai1, Twist1, Zeb1, Cdh1, Vimentin* and *Col1a1*. (n= 6 or 5(*Col1a1*) and 3 YFP sorted individual primary cell lines, respectively).

B Representative immunofluorescence histological micrographs of primary pancreatic tumors for YFP (green) and indicated mesenchymal markers (red): Snail/Slug, Slug, Twist, Zeb1, Vimentin, αSMA, or FSP1 and DAPI (blue) or E-cadherin (white) and Cytokeratin 8 (red) (n=8 and 5 mice, respectively). Data presented as the mean ± s.e.m.

C Number of YFP^+ cells in 1 ml of blood from KPC control (n=5) and KPC; ST (n = 6) mice.

D Percent YFP^+ single cells per um^2 of liver tissue in KPC control (n=4) and KPC; ST (n = 5) mice.

E Quantification of percent Ki-67^+ cells in immunostained histological micrographs. ANOVA.

F Select enriched gene sets comparing Clusters 30, 32 and 34 vs. all other cancer cell clusters, all pathways displayed are significant with a nominal p-value and FDRqvalue <0.05.

Unless otherwise specified, data presented as the means ± SD. and significance was determined by an unpaired two-tailed t-test. Scale bar equals 100 μm. ns = not significant, * p<0.05, *** p<0.001, **** p<0.0001.

Figure S4

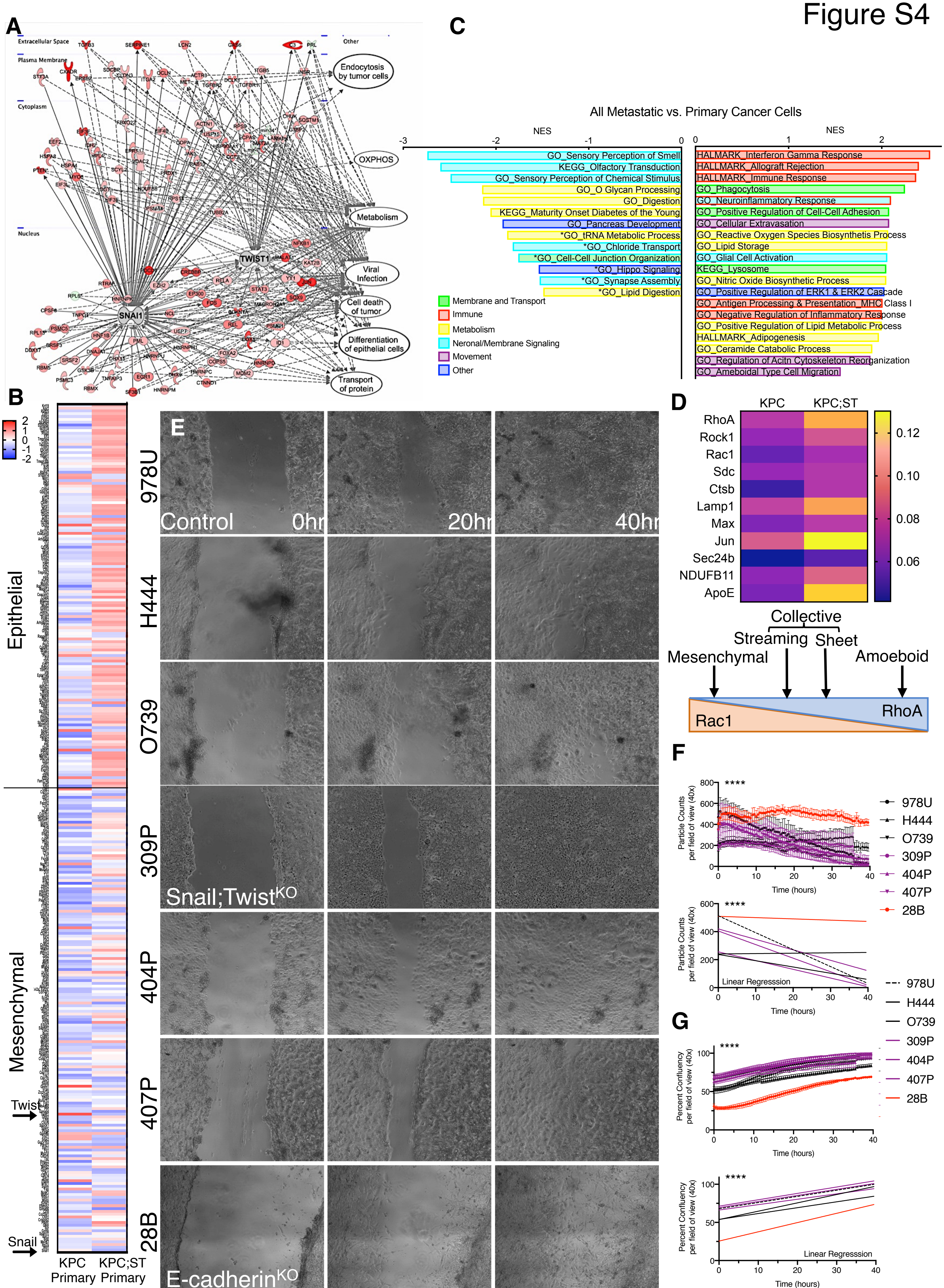
A
Extracellular Space
Plasma Membrane
Cytoplasm
Nucleus
Other
Endocytosis by tumor cells
OXPHOS
Metabolism
Viral Infection
Cell death of tumor
Differentiation of epithelial cells
Transport of protein
TWIST1
SNAI1
B
Epithelial
Mesenchymal
Twist
Snail
KPC Primary
KPC;ST Primary
C
All Metastatic vs. Primary Cancer Cells
NES
GO_Sensory Perception of Smell
KEGG_Olfactory Transduction
GO_Sensory Perception of Chemical Stimulus
GO_O Glycan Processing
GO_Digestion
KEGG_Maturity Onset Diabetes of the Young
GO_Pancreas Development
*GO_tRNA Metabolic Process
*GO_Chloride Transport
*GO_Cell-Cell Junction Organization
*GO_Hippo Signaling
*GO_Synapse Assembly
*GO_Lipid Digestion
HALLMARK_Interferon Gamma Response
HALLMARK_Allograft Rejection
HALLMARK_Immune Response
GO_Phagocytosis
GO_Neuroinflammatory Response
GO_Positive Regulation of Cell-Cell Adhesion
GO_Cellular Extravasation
GO_Reactive Oxygen Species Biosynthetis Process
GO_Lipid Storage
GO_Glial Cell Activation
KEGG_Lysosome
GO_Nitric Oxide Biosynthetic Process
GO_Positive Regulation of ERK1 & ERK2 Cascade
GO_Antigen Processing & Presentation_MHC Class I
GO_Negative Regulation of Inflammatory Response
GO_Positive Regulation of Lipid Metabolic Process
HALLMARK_Adipogenesis
GO_Ceramide Catabolic Process
GO_Regulation of Acitn Cytoskeleton Reorganization
GO_Ameboidal Type Cell Migration
Membrane and Transport
Immune
Metabolism
Neronal/Membrane Signaling
Movement
Other
D
KPC
KPC;ST
RhoA
Rock1
Rac1
Sdc
Ctsb
Lamp1
Max
Jun
Sec24b
NDUFB11
ApoE
0.12
0.10
0.08
0.06
Collective
Streaming
Sheet
Mesenchymal
Amoeboid
Rac1
RhoA
E
978U
H444
O739
309P
404P
407P
28B
Control
0hr
20hr
40hr
Snail;TwistKO
E-cadherinKO
F
Particle Counts per field of view (40x)
Time (hours)
978U
H444
O739
309P
404P
407P
28B
Linear Regresssion
G
Percent Confluency per field of view (40x)
Time (hours)
Linear Regresssion
****

Figure S4. Stabilized epithelial PDAC cells migrate by collective cell migration, Related to Figure 4.

A IPA pathway analysis of primary KPC control versus KPC;ST cancer cells.

B Z-score of the Thiery EMT signature demonstrating an overall stabilization of epithelial genes in the KPC;ST primary tumors.

C Select enriched gene sets comparing metastatic cancer cells vs. primary cancer cells, all pathways displayed are significant with a nominal p-value, FDRqvalue and FWER p-value <0.05. *only the nominal p-value <0.05.

D Mean qPCR 1/deltaCT values of YFP+ lineage traced primary cancer cells; schematic of RhoA and Rac1 expression in migration types.

E Representative micrographs (40x) of each primary cell line.

F and **G** Quantifications of the particle counts (**F**) and percent cellular confluency (**G**) with linear regressions (**** p< 0.001) of the grouped values for each cell line represented.

Figure S5

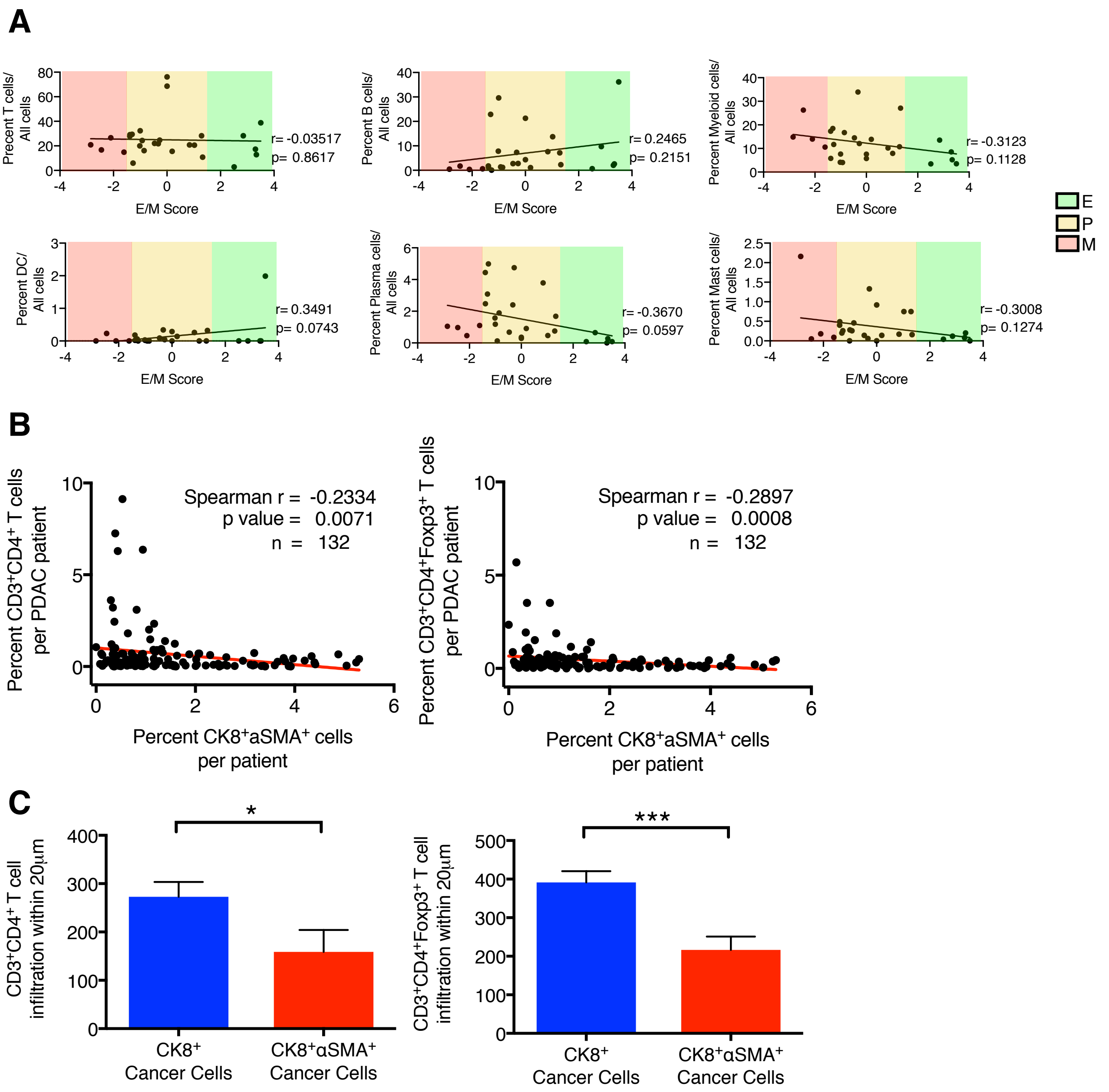
A
Percent T cells/ All cells
r= -0.03517
p= 0.8617
E/M Score
Percent B cells/ All cells
r= 0.2465
p= 0.2151
Percent Myeloid cells/ All cells
r= -0.3123
p= 0.1128
E
P
M
Percent DC/ All cells
r= 0.3491
p= 0.0743
Percent Plasma cells/ All cells
r= -0.3670
p= 0.0597
Percent Mast cells/ All cells
r= -0.3008
p= 0.1274
B
Percent CD3+CD4+ T cells per PDAC patient
Spearman r = -0.2334
p value = 0.0071
n = 132
Percent CK8+aSMA+ cells per patient
Percent CD3+CD4+Foxp3+ T cells per PDAC patient
Spearman r = -0.2897
p value = 0.0008
n = 132
C
CD3+CD4+ T cell infiltration within 20μm
*
CK8+ Cancer Cells
CK8+αSMA+ Cancer Cells
CD3+CD4+Foxp3+ T cell infiltration within 20μm
***

Figure S5. Epithelial PDAC cells associate with more T cells, Related to Figure 5.

A Correlation plots comparing the percent of indicated immune cells to the E/M score of each scRNA-seq patient tumor.

B Inverse correlation of between $CD4^+$ or $CD4^+FOXP3^+$ T cells and $CK8^+\alpha SMA^+$ cancer cells from multiplexed immunohistochemistry stained tissues.

C L-function area under the curve values (reflecting the number of cells) for $CD4^+$ or $CD4^+FOXP3^+$ T cells within 20 um of $CK8^+$ or $CK8^+\alpha SMA^+$ cancer cells.

D Quantification of percent Ki-67^+ cells in immunostained histological micrographs. ANOVA. * $p< 0.05$, *** $p<0.001$.

Figure S6

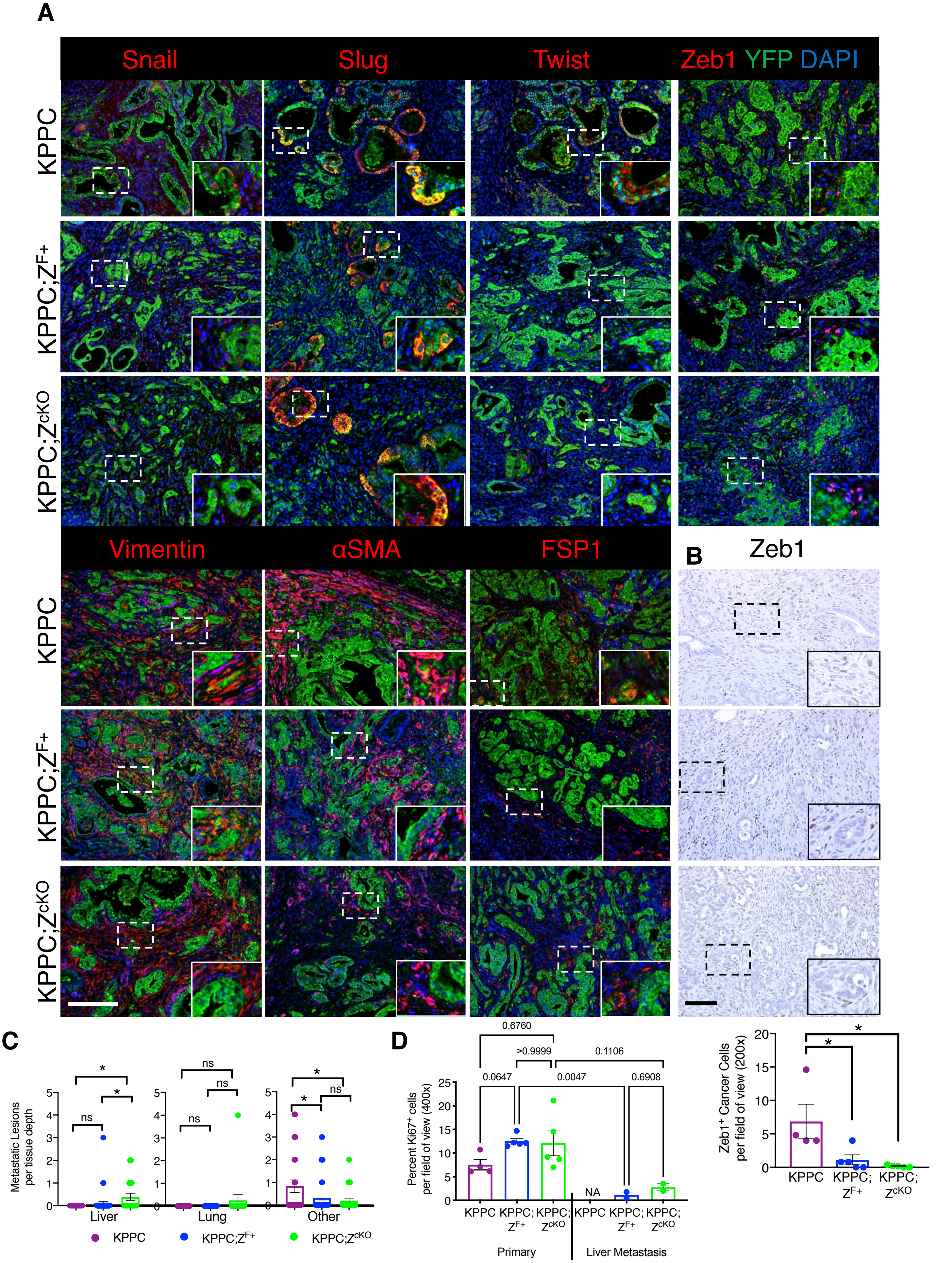

A
Snail
Slug
Twist
Zeb1 YFP DAPI
KPPC
KPPC;ZF+
KPPC;ZcKO
Vimentin
αSMA
FSP1
B
Zeb1
C
Metastatic Lesions per tissue depth
ns
*
Liver
Lung
Other
KPPC
KPPC;ZF+
KPPC;ZcKO
D
Percent Ki67+ cells per field of view (400x)
0.6760
>0.9999
0.1106
0.0647
0.0047
0.6908
NA
Primary
Liver Metastasis
Zeb1+ Cancer Cells per field of view (200x)

Figure S6. Stabilization of epithelial PDAC cells via Zeb1 ablation also enhances liver metastasis, Related to Figure 6.

A Representative histological micrographs of primary pancreatic tumors for YFP (green) and indicated mesenchymal markers (red): Snail/Slug, Slug, Twist, Zeb1, Vimentin, αSMA, or FSP1 and DAPI (blue).

B Immunohistochemical labeling for Zeb1, counterstained with hematoxylin and quantifications of Zeb1 positive cancer cells per image per mouse (n=4, 5, and 5 mice, respectively).

C Quantification of the metastatic leisions per tissue depth for liver (KPPC (n = 16 depths, 4 mice), KPPC;$Z^{F/+}$ (n = 48 depths, 12 mice) and KPPC; Z^{cKO} (n = 20 depths, 5 mice)), lung (KPPC (n = 12 depths, 4 mice), KPPC;$Z^{F/+}$ (n = 36 depths, 12 mice) and KPPC; Z^{cKO} (n = 15 depths, 5 mice)), and other tissues (KPPC (n = 50 depths, 4 mice), KPPC;$Z^{F/+}$ (n = 72 depths, 12 mice) and KPPC; Z^{cKO} (n = 30 depths, 5 mice)).

Unless otherwise specified, data presented as the mean ± s.e.m., scale bar equals 100 μm, and significance determined by a one-way ANOVA or the Kruskal-Wallis test when the data were not normally distributed. ns = not significant, * $p<0.05$.

Table S1. Clinical correlations between PDAC classification subtypes, Related to Figure 1.

Group	Parameter	Pearson Chi-Square
**Thiery EMT – sc-seq**		
EvPvM	Sex	0.350
EvPvM	Diabetes	0.842
EvPvM	Procedure	0.599
EvPvM	Location	0.773
EvPvM	T (TNM Classification)	0.787
EvPvM	N (TNM Classification)	0.583
EvPvM	M (TNM Classification)	NA - all M0
EvPvM	Staging	0.625
EvPvM	Perineural Invasion	0.435
EvPvM	Vascular Invasion	0.535
EvPvM	Peripancreatic Invasion	0.580
**Thiery EMT -TCGA**		
EvPvM	Sex	0.933
EvPvM	Histologic Grade	0.216
EvPvM	Tumor Stage	0.078
EvPvM	T (TNM Classification)	0.461
EvPvM	N (TNM Classification)	0.186
EvPvM	M (TNM Classification)	0.096
EvPvM	Radiation	0.646
**Moffitt -TCGA**		
Classical v Basal	Sex	0.706
Classical v Basal	Histologic Grade	0.096
Classical v Basal	Tumor Stage	0.772
Classical v Basal	T (TNM Classification)	0.810
Classical v Basal	N (TNM Classification)	0.588
Classical v Basal	M (TNM Classification)	0.095
Classical v Basal	Radiation	0.791
**Collisson -TCGA**		
Classical v Exocrine v QM	Sex	0.345
Classical v Exocrine v QM	Histologic Grade	0.225
Classical v Exocrine v QM	Tumor Stage	**0.013** *StageIIb with QM
Classical v Exocrine v QM	T (TNM Classification)	**0.022** *T2 with QM
Classical v Exocrine v QM	N (TNM Classification)	0.406
Classical v Exocrine v QM	M (TNM Classification)	0.153
Classical v Exocrine v QM	Radiation	0.247
**Chang-Seng-Yue-TCGA**		
Classical v Hybrid v Basal	Sex	0.930
Classical v Hybrid v Basal	Histologic Grade	0.061
Classical v Hybrid v Basal	Tumor Stage	0.078
Classical v Hybrid v Basal	T (TNM Classification)	0.783
Classical v Hybrid v Basal	N (TNM Classification)	0.121

Classical v Hybrid v Basal	M (TNM Classification)	0.059
Classical v Hybrid v Basal	Radiation	**0.0022** *less in Classical
**Cross Groups**	**Significant Groups**	
Thiery v Moffitt		0.572
Thiery v Collisson	E with Classical, M with QM	**0.000000007**
Thiery v Chang-Seng-Yue	E with Classical, P&M with Hybrid and Basal A/B	**0.00000008**

P< 0.05

Table S2. Survival and Pathology of murine cohorts, Related to Figure 3 and 6.

Survival and pathology of murine cohorts

						**Presence of Metastasis**				
**ID**	**Sex**	**Age at Palpable Tumor**	**Age at Death**	**PDA**	**Primary Score**	**Liver**	**Lung**	**Other**	**Any**	**Moribund**
**KPC**		**(75.5)**	**(135)**							
1	F	-	150	Y	P	Y	Y	Y	Y	FD
2	M	47	92	Y	P	Y	N	Y	Y	Y
3	F	98	140	Y	M	N	N	N	N	N
4	M	91	106	Y	P	N	N	Y	Y	Y
5	F	-	108	Y	-	-	-	-	-	FD
6	F	-	234	Y	P	N	N	Y	Y	Y
7	F	105	154	Y	P	N	N	Y	Y	N
8	F	73	80	Y	P	Y	N	Y	Y	Y
9	F	78	115	Y	P	N	Y	Y	Y	FD
10	F	98	155	Y	-	-	-	-	-	FD
11	F	91	135	Y	P	N	Y	N	Y	N
12	M	69	157	Y	P	N	N	N	N	Y
13	M	73	97	N	PanIN	N	N	N	N	N
14	M	54	203	Y	W	Y	Y	Y	Y	Y
15	F	69	88	Y	-	Y	N	N	Y	FD
**KPC; Snail$^{F/+}$;Twist$^{F/+}$**		**(88)**	**(153)**							
16	F	142	153	Y	W	N	N	N	N	N
17	M	88	243	Y	P	N	N	Y	Y	FD
18	F	95	101	Y	W	Y	N	N	Y	FD
19	M	82	111	Y	P	Y	Y	Y	Y	Y
20	F	69	228	Y	M	Y	N	Y	Y	Y
21	M	-	163	Y	-	-	-	-	-	FD
22	F	77	127	Y	-	-	-	-	-	FD
Totals			(Median)	14/15		5/13	4/13	8/13	10/13	
%				93.3%		38.5%	30.8%	61.5%	76.9%	
Totals with S$^{F/+}$T$^{F/+}$			(Median)	21/22		8/18	6/18	11/18	14/18	
%				95.5%		44.4%	33.3%	61.1%	77.7%	
**KPC;ST**		**(90)**	**(151)**							
1	M	-	111	Y	P	Y	Y	Y	Y	FD
2	F	102	241	Y	P	Y	Y	Y	Y	Y
3	F	106	155	Y	P	Y	N	Y	Y	N
4	F	95	144	Y	W	N	N	N	N	N
5	M	85	134	Y	W	N	N	N	N	N
6	F	106	157	Y	P	Y	N	Y	Y	Y
7	F	120	157	Y	P	N	N	N	N	N
8	F	-	91	Y	P	N	N	N	N	N
9	M	86	113	Y	P	N	N	N	N	FD
10	M	-	130	N	PanIN	N	N	N	N	N

11	F	57	85	Y	W	N	N	N	N	N
12	M	112	230	Y	-	Y	-	-	-	N
13	M	76	146	Y	W	N	N	N	N	N
14	M	69	146	Y	P	Y	Y	Y	Y	Y
15	M	67	198	Y	M	Y	N	Y	Y	Y
16	M	69	180	Y	P	Y	Y	N	Y	FD
17	M	85	104	Y	P	N	N	N	N	FD
18	F	124	230	Y	P	Y	N	N	Y	Y
19	F	116	185	Y	P	Y	Y	Y	Y	Y
20	M	82	132	Y	-	-	-	-	-	N
21	F	85	149	Y	-	-	-	-	-	FD
22	M	74	176	Y	W	N	N	N	N	N
23	M	60	157	Y	P	N	N	N	N	Y
24	F	138	195	Y	M	Y	N	N	Y	N
25	M	146	195	Y	W	N	N	N	N	N
26	F	54	146	Y	P	N	N	Y	Y	Y
27	F	109	132	Y	W	N	N	N	N	N
28	F	102	147	Y	M	N	Y	N	Y	Y
29	F	110	159	Y	-	-	-	-	-	Y
30	M	67	152	Y	W	Y	Y	Y	Y	Y
31	F	97	104	Y	W	N	N	N	N	N
32	M	90	212	Y	M	Y	Y	N	Y	N
Totals			(Median)	31/32		13/29	8/29	8/29	14/29	
%				96.9%		44.8%	27.6%	27.6%	48.3%	
**KPPC**		**(39)**	**(47)**							
1	F	-	43	Y	P	N	N	Y	Y	Y
2	M	39	47	Y	M	N	N	Y	Y	FD
3	M	39	47	Y	W	N	N	N	N	FD
4	M	41	51	Y	M	N	N	Y	Y	Y
Totals			(Median)	4/4		0/4	0/4	3/4	9/13	
%				100.0%		0.0%	0.0%	75.0%	69.2%	
**KPPC;Z $^{F/+}$**		**(38)**	**(53.5)**							
1	M	39	54	Y	W	N	N	N	N	N
2	M	27	53	Y	P	N	N	N	N	N
3	M	27	53	Y	P	N	N	Y	Y	N
4	M	27	53	Y	P	N	N	Y	Y	Y
5	F	27	53	Y	W	N	N	Y	Y	Y
6	F	35	60	Y	W	N	N	Y	Y	N
7	M	46	64	Y	P	Y	N	Y	Y	Y
8	F	37	73	Y	W	N	N	Y	Y	Y
9	F	41	41	Y	M	N	N	N	N	Y
10	F	41	51	Y	W	Y	N	N	Y	Y
11	F	41	66	Y	P	N	N	N	N	Y

12	M	41	77	Y	P	N	N	Y	Y	N
Totals			(Median)	12/12		2/12	0/12	7/12	8/12	
%				100.0%		16.7%	0.0%	58.3%	66.7%	
**KPPC;Z^{cKO}**		**(39)**	**(65.5)**							
1	F	37	47	Y	M	N	N	Y	Y	Y
2	M	30	66	Y	P	Y	N	Y	Y	Y
3	F	-	35	N	Normal/ PanIn	N	N	N	N	N
4	M	55	65	Y	P	N	N	Y	Y	Y
5	M	39	90	Y	W	Y	Y	Y	Y	Y
6	M	55	80	Y	W	N	N	N	N	Y
Totals		(Median)		5/6		2/5	1/5	4/5	4/5	
%				83.3%		40.0%	20.0%	80.0%	80.0%	

Key: (Y) yes. (N) no, (W) well, (M) moderate, (P) poor, (FD) found dead, (-) no tissue

Table S3.
Disease correlations between murine cohorts, Related to Figure 3 and 6.

Group	Parameter	*P* value or ^Chi-square	
**Microscopic Metastasis**		**all**	**Endpoint**
Control vs. $Snail^{cKO}$;$Twist^{cKO}$	Sex	0.581	1.000
KPPC vs. KPPC;$Z^{F/+}$ vs. KPPC;Z^{cKO}		^0.615	^0.143
Control vs. $Snail^{cKO}$;$Twist^{cKO}$	Liver Metastasis	1.000	1.000
KPPC vs. KPPC;$Z^{F/+}$ vs. KPPC;Z^{cKO}		^0.400	^0.371
Control vs. $Snail^{cKO}$;$Twist^{cKO}$	Lung Metastasis	1.000	0.440
KPPC vs. KPPC;$Z^{F/+}$ vs. KPPC;Z^{cKO}		^0.247	^0.309
Control vs. $Snail^{cKO}$;$Twist^{cKO}$	Other Metastasis	0.072	0.420
KPPC vs. KPPC;$Z^{F/+}$ vs. KPPC;Z^{cKO}		^0.822	^0.668
Control vs. $Snail^{cKO}$;$Twist^{cKO}$	Any Metastasis	0.072	0.596
KPPC vs. KPPC;$Z^{F/+}$ vs. KPPC;Z^{cKO}		^0.949	^0944
**Macroscopic Metastasis**		**all**	**Endpoint**
Control vs. $Snail^{cKO}$;$Twist^{cKO}$	Liver Metastasis	0.762	0.710
KPPC vs. KPPC;$Z^{F/+}$ vs. KPPC;Z^{cKO}		**^0.010**	**^0.017**
Control vs. $Snail^{cKO}$;$Twist^{cKO}$	Lung Metastasis	0.267	0.483
KPPC vs. KPPC;$Z^{F/+}$ vs. KPPC;Z^{cKO}		na (all neg)	na (all neg)
Control vs. $Snail^{cKO}$;$Twist^{cKO}$	Any Metastasis	0.316	0.169
KPPC vs. KPPC;$Z^{F/+}$ vs. KPPC;Z^{cKO}		^0.615	^0.355
Control vs. $Snail^{cKO}$;$Twist^{cKO}$	Ascites	1.000	0.700
KPPC vs. KPPC;$Z^{F/+}$ vs. KPPC;Z^{cKO}		^0.095	^0.202

***P*< 0.05**

Table S4: Antibodies, Related to Figures 3,5 and 6.

Antigen	Antigen Retrieval	Blocking	Primary antibody Concentration	Primary antibody Catalog Number	Secondary antibody	Secondary antibody Concentration	Secondary antibody Company	Opal fluorophore Dye	Opal fluorophore Concentration
Slug	Citrate Buffer 15min Microwave	4% CWFG	1:200	cs9585	Rabbit on Rodent Polymer	stock	BioCare	Opal 540	1:100
FSP1	Citrate Buffer 15min Microwave	1% BSA	1:6,00	A5114	Rabbit on Rodent Polymer	stock	BioCare	Opal 570	1:2,000
Zeb1	Citrate Buffer 30min Microwave	1% BSA	1:2,000	NBP1-05987	Rabbit on Rodent Polymer	stock	BioCare	Opal 520	1:50
Cytokeratin 8	Citrate Buffer 45min Microwave	1% BSA	1:50	TROMA-1	Po-link Rat Polymer	stock	GBI Labs	Opal 690	1:200
YFP	Citrate Buffer 60min Microwave	4% CWFG	1:30,000	GFP-1020	Po-link Chicken Polymer	stock	GBI Labs	Opal 650	1:1,000
Twist1	Citrate Buffer 75min Microwave	MOM Kit blocking - Vector Labs	1:200	NBP2-37364	Mouse on Mouse Polymer	stock	BioCare	Opal 620	1:100
Vimentin	Citrate Buffer 75min Microwave	1% BSA	1:1,000	cs5741	Rabbit on Rodent Polymer	stock	BioCare	Opal 520	1:50
Snail/Slug	TrisEDTA 1hour pressure cooker	4% CWFG	1:20,000	ab180714	Rabbit on Rodent Polymer	stock	BioCare	Opal 570	1:1,000
aSMA	Citrate Buffer 90min Microwave	1% BSA	1:500	M0851	Mouse on Mouse Polymer	stock	BioCare	Coumarin	1:50
E-cadherin	Citrate Buffer 15min Microwave	1% BSA	1:100	CS3195S	Goat-antiRabbit AF-488	1:500	Invitrogen	NA	NA
YFP			1:500	GFP1020	Goat-antiChicken AF-594	1:1,000	Invitrogen		
Cytokeratin 8			1:50	TROMA-1	Goat-antiRat AF-647	1:1,000	Invitrogen		
E-cadherin	Citrate Buffer 15min Microwave	1% BSA	1:100	CS3195S	Goat-antiRabbit Biotin	1:250	Vector Labs	Vector Labs ABC kit	
Ki67	Citrate Buffer 1hour Microwave	4% CWFG	1:400	Thermo RM9106S1	Goat-antiRabbit Biotin	1:250	Vector Labs	Vector Labs ABC kit	
Zeb1	Citrate Buffer 15min Microwave	5% Goat Serum	1:500	NBP1-05987	Goat-antiRabbit Biotin	1:250	Vector Labs	Vector Labs ABC kit	
CD8	Citrate Buffer 15min Microwave	10% Goat Serum	1:500	Biorbyt orb10325	Rabbit on Rodent Polymer	stock	BioCare	Opal 540	1:100
CD4	Citrate Buffer 30min Microwave	0.1% Triton, 1% goat serum, 1% BSA	1:10,000	SinoBiolo 50134-R001	Rabbit on Rodent Polymer	stock	BioCare	Opal 650	1:1000
Collagen I	Citrate Buffer 45min Microwave	1% BSA	1:500	AbDSerotec 131001	Po-link Goat Polymer	stock	GBI Labs	Coumarin	1:50
Cytokeratin 8	Citrate Buffer 60min Microwave	1% BSA	1:50	TROMA-1	Po-link Rat Polymer	stock	GBI Labs	Opal 520	1:500
aSMA	Citrate Buffer 75min Microwave	1% BSA	1:2,000	DAKO M0851	Mouse on Mouse Polymer	stock	BioCare	Opal 690	1:50
CD3	Citrate Buffer 90min Microwave	1% BSA	1:1,000	DAKO A0452	Rabbit on Rodent Polymer	stock	BioCare	Opal 620	1:500
Foxp3	Citrate Buffer 105min Microwave	1% BSA	1:2,000	eBioscience 14-4771-80	Po-link Rat Polymer	stock	GBI Labs	Opal 570	1:100
